# Refoldable Foldamers: Global Conformational Switching by Deletion or Insertion of a Single Hydrogen Bond

**DOI:** 10.1002/anie.201510605

**Published:** 2016-01-14

**Authors:** Bryden A. F. Le Bailly, Liam Byrne, Jonathan Clayden

**Affiliations:** ^1^ School of ChemistryUniversity of BristolCantock's CloseBristolBS8 1TSUK; ^2^ School of ChemistryUniversity of ManchesterOxford RoadManchesterM13 9PLUK

**Keywords:** biocatalysis, conformation, foldamers, peptides, photoswitches

## Abstract

Small changes in the structure of a foldamer may lead to gross changes in conformational preference. We show that the simple insertion or deletion of a single hydrogen bond by changes in pH or by photochemical deprotection is sufficient to refold a helical oligomer, interconverting M and P screw‐sense preference. As a consequence of the switch, information may be transmitted to a remote catalytic site, selectively directing the formation of either of two enantiomeric products by a reaction involving 1,22‐remote intermolecular asymmetric induction.

The higher order structure of proteins and peptides is generally remarkably tolerant of modifications of primary structure:^1^ the serine proteases, for example, display similar tertiary structures and have near‐identical functions across a wide range of organisms, despite variations in their primary sequence of up to 50 %.^2^ Indeed, it can be argued that the tolerance of variation in protein structure is what allows the process of random mutation and natural selection to proceed.^3^ Nonetheless, there are some proteins in which a small conformational change, such as the *cis*/*trans* isomerisation of a proline residue, is sufficient to modulate function.^4^, ^5^

Foldamers^6^ are synthetic analogues of biomolecules^7^ into which conformational features are programmed to allow biomimetic function.^8^, ^9^, ^10^ Never having been subjected to the process of evolution, it is to be expected that the designed structures of foldamers contain less redundancy than natural proteins, allowing small modifications to result in gross changes in structural features. Foldamers have thus been designed that undergo conformational switches as a result of changes in solvent, temperature, non‐covalent ligand binding or modifications to the foldamer backbone.^11^, ^12^, ^13^, ^14^

Here we show that even a change as minimal as insertion or deletion of a single hydrogen bond, initiated by a photochemical switch or a change in pH, may lead to a global conformational switch in a group of peptide‐like foldamers. The foldamers in question were designed to favour strongly a global helical conformation by building them from the powerfully helicogenic quaternary amino acid Aib. Homo‐oligomers of Aib of greater than three residues are essentially entirely 3_10_ helical,^15^, ^16^, ^17^, ^18^, ^19^, ^20^ especially in non‐polar solvents.^21^ Oligomers of Aib diverge subtly from Gellman's original definition of a foldamer,^6^ since they populate not one but two principal conformations of opposite screw sense: in an entirely achiral oligomer these rapidly interconverting conformers^22^ are enantiomeric and therefore necessarily isoenergetic.

The two screw‐sense conformers of an Aib oligomer may be desymmetrised by ligation to a chiral terminus,^23^, ^24^, ^25^, ^26^, ^27^, ^28^, ^29^, ^30^, ^31^, ^32^, ^33^ which leads to an imbalance in the population of the two interconverting screw‐sense conformers.^34^,^35^ This imbalance may be quantified by NMR,^27^ and a C terminal AlaNHR residue, for example, may induce a 99:1 preference for right‐handed screw sense in an attached Aib oligomer chain.^25^ The ability of a terminal residue to induce a screw‐sense preference is allied to the way in which it organises the final β turn of the 3_10_ helix, and we set out to devise systems in which the simple addition or deletion of a hydrogen bond was sufficient to lead to a fundamental change in the structure of this structure and hence a global modification of conformation.

Oligomer **4** bearing Ala as a C terminal inducer of screw‐sense preference was synthesised as shown in Figure 1. The C terminal hydrogen bond of the Ala residue was “erased” chemically by functionalisation as a tertiary amide derivative using the photosensitive 5‐bromo‐7‐nitroindoline (Bni) **1**,^36^ which was ligated to l‐alanine without racemisation^37^, ^38^ to give **2** as a coupling partner. Alaninamide **2** was coupled to an Aib pentamer **3** in which the N terminal residue was enantioselectively isotopically enriched with ^13^C in its pro‐*R* and pro‐*S* methyl groups in a 75:25 ratio,^39^ giving the helical^25^ oligomer **4**.

**Figure 1:**
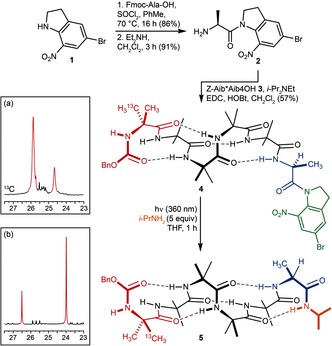
Photochemically‐induced refolding of a left‐handed into a right‐handed helix. Insets: a) portion of ^13^C NMR spectrum of **4**; b) portion of ^13^C DEPT NMR spectrum of **5** in [D_8_]THF.

VT ^13^C NMR studies^27^, ^33^ of **4** showed that it displayed a 72:28 preference^40^ for a left‐handed^41^ screw sense at 25 °C in THF (Figure 1 a). The left‐handed helical oligomer **4** was irradiated at 360 nm for 1 h in [D_8_]THF in the presence of an excess of isopropylamine. Under these conditions, the tertiary amide fragments to a reactive *O*‐acyl nitronate,^36^ which is trapped by the amine to form secondary amide **5** in 97 % yield. The exchange in position of the major and minor signals in the ^13^C NMR spectrum of **5** (Figure 1 b) indicated that the oligomer has entirely refolded from a left‐ to a right‐handed helix as a result of this photochemically‐induced switch of a tertiary for a secondary amide. **5** adopts a *P* screw sense^41^ with a 99:1 helical ratio.^41^ The helical inversion presumably results from the introduction of a new hydrogen bond donor at the C terminus of the oligomer,^25^ inducing the formation of new β‐turn (as shown for **5**) in place of the extended structure at the C terminus of **4**, and thus the global reversal of screw‐sense preference.

The selective deletion or re‐formation of a terminal hydrogen bond could alternatively be achieved by removal or addition of a single proton by treatment with base or acid. Achieving the regioselectivity necessary for selective deprotonation/reprotonation of a compound containing numerous N−H bonds requires the incorporation of a structure that induces a substantial decrease in p*K*_a_ of a specific N−H group. Metal ions such as Ni^2+^ or Cu^2+^ coordinate amino acid residues in peptides through their amide N atoms, acidifying the amide group and lead to spontaneous deprotonation.^42^, ^43^ A metal binding site was thus ligated to the C‐terminus of foldamer **8** with the aim of localising binding of a metal to the C terminal amide linkage and promoting deprotonation of the C‐terminal N−H (Figure 2, shown in orange). To give well‐defined monomeric structures, the tridentate tren structure **6** was chosen as a binding site to satisfy three of the metal ion's coordination sites. Tetramine **6** was selectively doubly Boc protected at its primary amino groups^44^ and ligated to l‐alanine to yield **7**, which was coupled to labelled foldamer **3** to give **8**.

**Figure 2:**
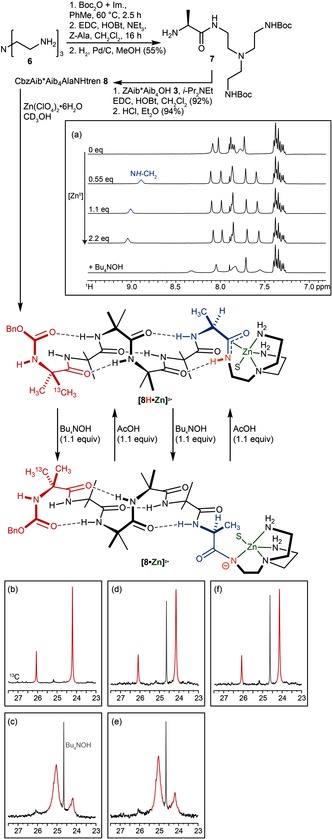
pH‐induced refolding of a left‐handed into a right‐handed helix. S=solvent. Insets: a) Change in ^1^H NMR spectrum on addition of 0–2.2 equiv Zn(ClO_4_)_2_ to **8**. b–f) Portions of ^13^C NMR spectrum of **8** (b) after complexation with Zn^2+^; c) after adding Bu_4_NOH (1.1 equiv); d) after adding AcOH (1.1 equiv); e) after adding further Bu_4_NOH (1.1 equiv); f) after adding further AcOH (1.1 equiv).

In order to allow analysis by NMR, the C terminal amide NH bond of **8** was acidified with Zn^2+^ (rather than Cu or Ni) cations.^43^ Zn(ClO_4_)_2_⋅6 H_2_O was added in portions to a solution of the oligomer **8** in CD_3_OH. ^1^H NMR (Figure 2 a) showed a significant change in chemical shift of only one NH, identified as the C terminal N*H*CH_2_ of the helix by its triplet multiplicity. The signal migrated from δ 7.78 to 9.02 ppm on addition of 1 equiv of Zn(ClO_4_)_2_, but shifted very little on further addition of the salt, confirming the 1:1 metal binding stoichiometry. The ^13^C spectrum of the complex (Figure 2 b) showed that the oligomer retained its right‐handed screw sense.

The downfield shift suggests acidification of the NH group by coordination of the terminal amide to Zn, but not spontaneous deprotonation. The complex formed from **8** and 1.1 equiv Zn(ClO_4_)_2_⋅6 H_2_O was therefore treated with Bu_4_NOH (1.1 equiv), a base with a non‐coordinating counterion. As a result, the N*H*CH_2_ group disappeared from the ^1^H NMR spectrum (Figure 2 a). The ^13^C NMR spectrum also showed a change in the position of the major and minor ^13^C labels,^14^ indicating a switch from in screw sense from *P* in **8**H⋅Zn^2+^ (Figure 2 b, with anisochronicity 1966 ppb, corresponding to 9:91 screw‐sense preference) to *M* in **8**⋅Zn^+^ (Figure 2 c, anisochronicity 860 ppb, corresponding to 68:32 screw‐sense preference).^25^ Neutralisation of the base with acetic acid (1.1 equiv) gave a ^13^C NMR spectrum (Figure 2 d) similar to that of **8**H⋅Zn^2+^, indicating complete restoration of the original screw‐sense preference. A second addition of base (Figure 2 e) and then acid (Figure 2 f) confirmed the repeatability of the pH‐directed refolding process, allowing interconversion of the two conformers.

Natural conformationally switchable proteins such as rhodopsin and other G‐protein coupled receptors function by setting in progress a chemical transformation as a result of a conformational change.^45^, ^46^ In order to allow synthetic analogues related to **4** and **8** the potential to translate a conformational change into a detectable change in chemical reactivity, we devised a range of achiral catalytic sites based on modifications of Takemoto's aminothiourea catalysts,^47^ and appended them to the N terminus of a right‐handed helical structure bearing a C terminal alanine residue. The resulting foldamers **9 a**–**f** contain a catalytically active site with no local chirality, whose conformation may nonetheless be induced to be asymmetric by a remote stereogenic centre two helical turns, or 13–16 bonds, away. These foldamers were used to catalyse the addition of dimethyl or diethyl malonate to β‐nitrostyrene (Figure 3 and Table 1). Only the less sterically hindered catalysts **9 e** and **9 f** were successful (entries 5–7); conversion with **9 a**–**9 d** was low (entries 1–4), irrespective of an anticipated beneficial Thorpe–Ingold effect.^48^ Despite the spatial separation of the catalytic site from the source of asymmetry, enantiomeric ratios of ca. 75:25 were obtained in favour of the *R* enantiomer of the product **10**, rising to 82:18 with dimethylmalonate as the nucleophile (entry 7). A control experiment (entry 8) in which the catalytic site and the asymmetric centre were located in different molecules demonstrated that the asymmetric induction was the result of intramolecular conformational induction: a 1:1 mixture of thiourea **9 g** and CbzGlyAib_4_AlaNHtBu **9 h** catalysed the formation of **10** in 68 % yield in essentially racemic form.

**Figure 3:**
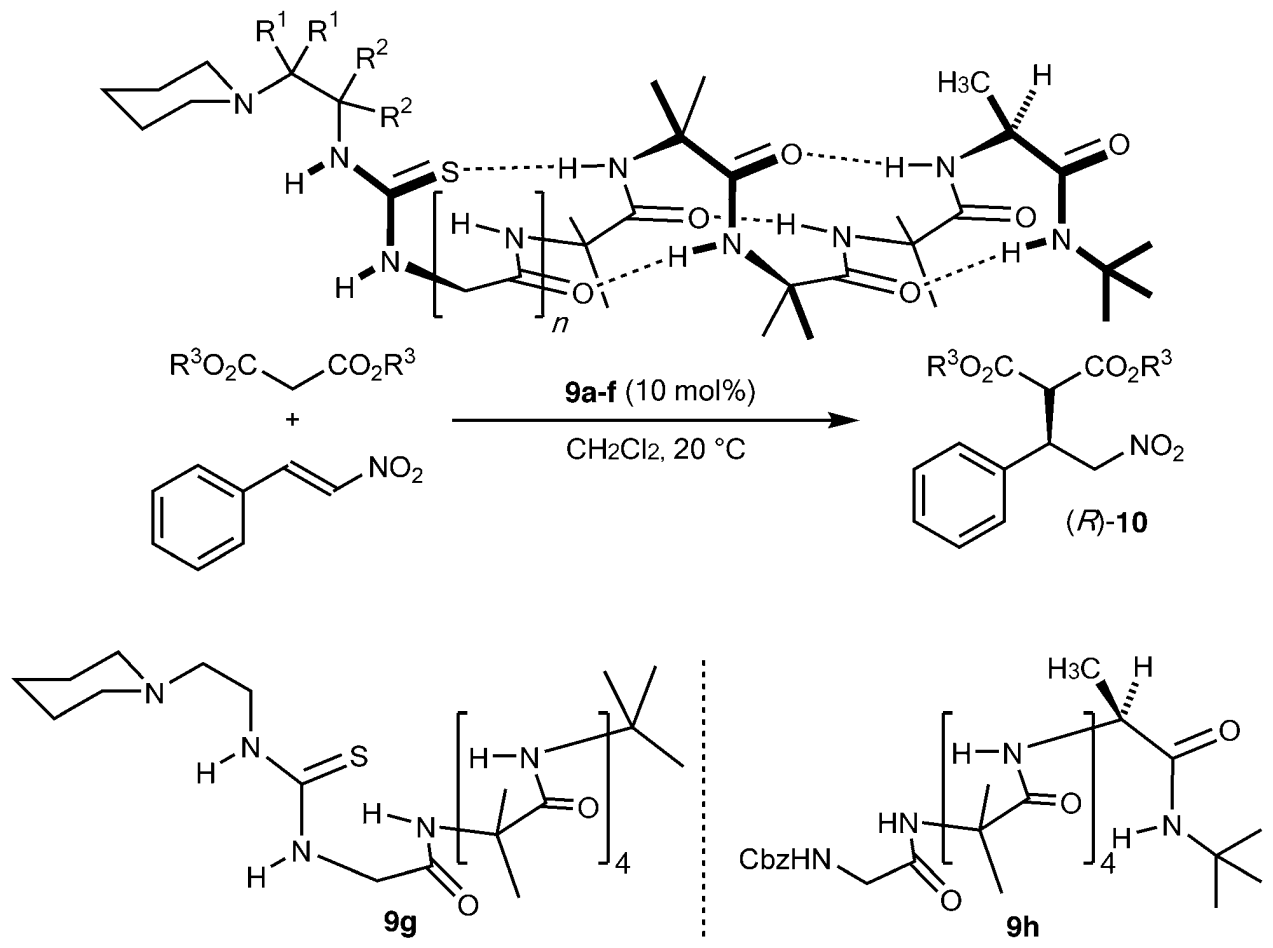
Catalytic foldamers with a remotely inducible catalytic site.

**Table 1:** Optimisation of a remotely inducible catalytic site: **9** as a catalyst for malonate addition to nitrostyrene (Figure 3).

Entry	Cat. **9**	R^1^	R^2^	R^3^	*n*=	% **10** ^[a]^	er^[b]^ **10**
1	**9 a**	H	Me	Et	0	<5^[c]^	–
2	**9 b**	H	Me	Et	1	<5^[c]^	–
3	**9 c**	Me	H	Et	0	<5	–
4	**9 d**	Me	H	Et	1	5, 12^[d]^	74:26
5	**9 e**	H	H	Et	0	33, 70^[e]^	71:29
6	**9 f**	H	H	Et	1	64	75:25
7	**9 f**	H	H	Me	1	85	82:18
8^[f]^	**9 g + 9 h**	–	–	–	–	68	52:48

[a] Conversion by NMR after 18 h. [b] Determined by HPLC on a chiral stationary phase. [c] After 48 h. [d] After 120 h. [e] After 96 h. [f] Intermolecular control experiment.

We propose that the asymmetric addition to nitrostyrene proceeds through a transition state approximating to that illustrated in Figure 4.^49^ Hydrogen bonding between the acidic malonate and the piperidine function at the N terminus of the *P* helix allows attack on the lower face of the thiourea‐bound nitrostyrene with the malonate ester substituents and nitrostyrene phenyl group avoiding the steric clash that would arise from attack on the other face of the nitrostyrene were inverted. The preferential formation of (*R*)‐**10** involves organocatalysis proceeding with unprecedentedly remote^33^, ^50^, ^51^ intermolecular 1,22 asymmetric induction.

**Figure 4:**
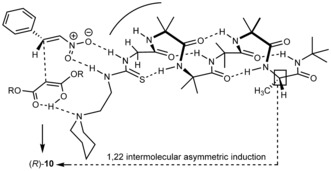
Proposed model for remote asymmetric induction.

The optimal catalytic site of **9 f** was coupled to an oligomer carrying at its C terminus the photoswitchable AlaBni residue, giving foldamer catalyst **11**. Addition of this catalyst to a mixture of dimethylmalonate and nitrostyrene in dichloromethane gave, after 18 h, (*S*)‐**10** in 37:63 er (Figure 5). The catalyst was then irradiated at 360 nm in the presence of isopropylamine, converting the tertiary amide into a secondary amide and inducing the helix to refold into a left‐handed conformation. The catalyst was again added to a mixture of dimethylmalonate and nitrostyrene in dichloromethane to give, after 72 h, a 78 % yield of (*R*)‐**10** in 77:23 er. Asymmetric organocatalysts with stereoselectivity switchable^52^ by light,^53^ by solvent^54^ or electrochemically^55^ have been reported, as have catalysts based on helical polymers with invertible screw sense.^56^, ^57^ In this “allosteric” example, the switchable source of asymmetric induction is remote from the site of catalysis and its sterochemical influence is relayed unidirectionally through a helical chain. The induced remote reversal of enantioselectivity demonstrates that insertion of a single hydrogen bond at a remote site in a refoldable molecule is sufficient to induce a global conformational switch that translates information about a small change in structure into a chemically significant, detectable result.

**Figure 5:**
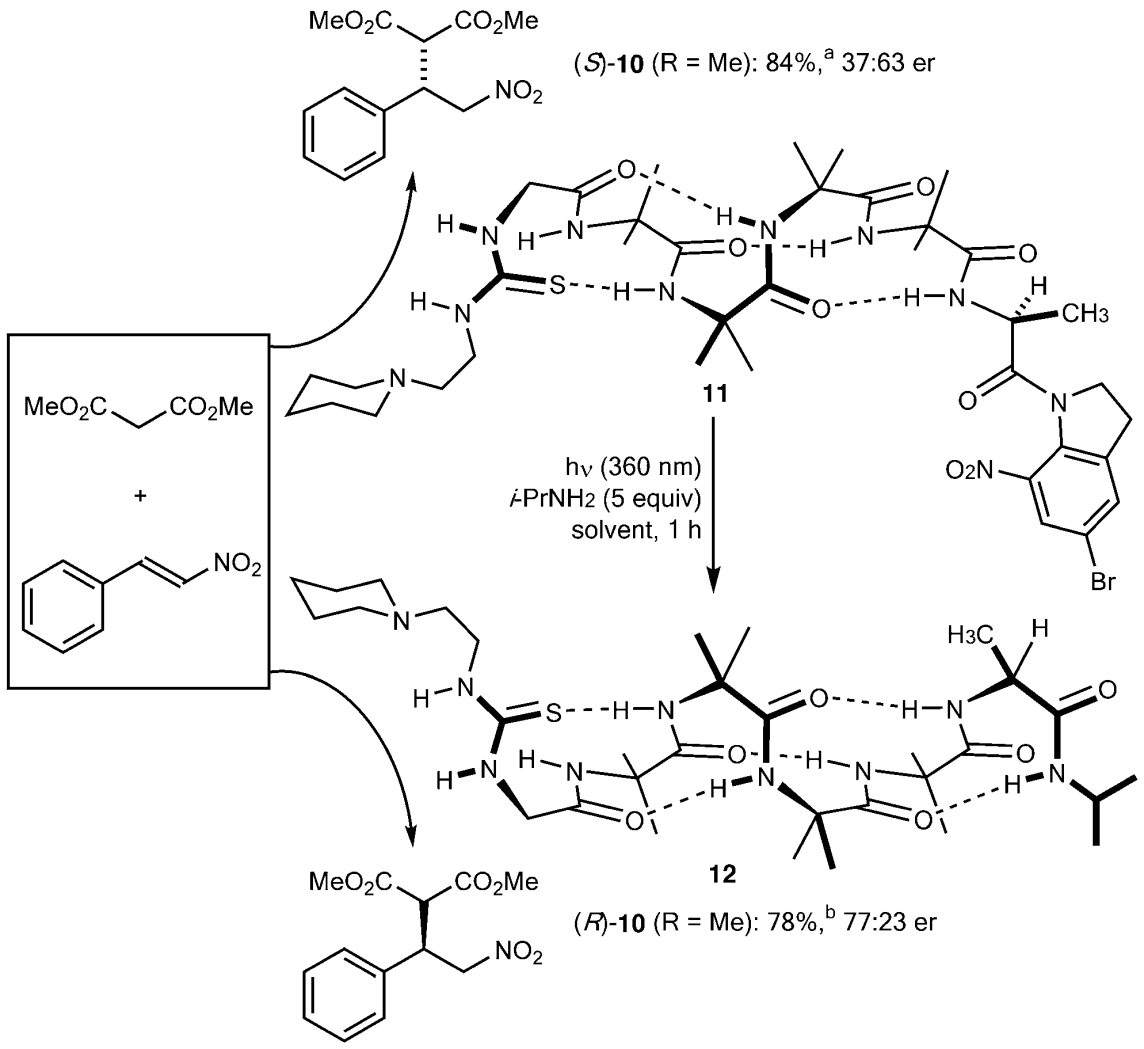
Photochemical refolding of a catalytic foldamer with consequent inversion of the sense of asymmetric induction.

## Supporting information

As a service to our authors and readers, this journal provides supporting information supplied by the authors. Such materials are peer reviewed and may be re‐organized for online delivery, but are not copy‐edited or typeset. Technical support issues arising from supporting information (other than missing files) should be addressed to the authors.

Supplementary
