# Supplementary material for: Refoldable Foldamers: Global Conformational Switching by Deletion or Insertion of a Single Hydrogen Bond
Source: Angew Chem Int Ed Engl. 2016 Jan 14;55(6):2132–6. doi: 10.1002/anie.201510605 (PMC4755161; doi:10.1002/anie.201510605)
Supplement: Supplementary file 1 — Supplementary [file ANIE-55-2132-s001.pdf]

Angewandte Chemie
Eine Zeitschrift der Gesellschaft Deutscher Chemiker

Supporting Information

Refoldable Foldamers: Global Conformational Switching by Deletion or Insertion of a Single Hydrogen Bond

*Bryden A. F. Le Bailly, Liam Byrne, and Jonathan Clayden**

anie_201510605_sm_miscellaneous_information.pdf

Supporting Information

Table of Contents

General Experimental and Materials	S2
Instrumentation	S2
Experimental Procedures	S3
^{1}H and ^{13}C NMR Spectra	S17
HPLC Traces	S39
References	S41

General Experimental and Materials

All reactions were carried out in oven-dried glassware under an atmosphere of nitrogen using standard anhydrous techniques. All reagents were obtained from commercially available sources and used without further purification, or where indicated prepared internally. Air- and moisture-sensitive liquids and solutions were transferred *via* syringe or stainless steel cannula. Reactions performed at 0 °C were done so using an ice bath. Anhydrous dichloromethane and tetrahydrofuran were obtained by distillation from calcium hydride, and sodium wire with a benzophenone indicator, respectively. Other anhydrous reaction solvents were obtained from standard anhydrous solvent engineering system. Petrol (or PE) refers to the fraction of light petroleum ether boiling between 40 and 65 °C. Triethylamine was stored over potassium hydroxide. All products were dried on a rotary evaporator followed by connection to a high vacuum system to remove any residual solvent. Flash chromatography was performed on silica gel (Merck 60H, 40-60 nm, 230-300 mesh). Analytical thin layer chromatography was performed on aluminium backed silica (60 F_{254}) plates.

Instrumentation

All ^{1}H and ^{13}C nuclear magnetic resonance spectra were obtained using Bruker AVANCE 400, 500 or 800 MHz spectrometers. Chemical shifts are quoted in parts per million (ppm), and coupling constants (*J*) are quoted in Hz to the nearest 0.5 Hz. ^{1}H-NMR spectra were referenced to the residual deuterated solvent peak ($CDCl_3$ 7.27; CD_3OD 3.31; THF-d_8 1.73 ppm) and ^{13}C-NMR were referenced to the carbon resonance of the solvent ($CDCl_3$ 77.00; CD_3OD 49.05; THF-d_8 25.37 ppm). Multiplicities are denoted as s (singlet), d (doublet), t (triplet), q (quartet), spt (septet) and m (multiplet) or denoted as br (broad), or some combination of these, where appropriate. Where ^{1}H-NMR spectra were run in CD_3OD exchangeable protons (NH, OH) are reported only where observed. Assignments were made using DEPT-135, 2D ^{1}H-COSY and HMQC experiments.

Infra-red spectra were recorded on an ATi Perkin Elmer Spectrum RX1 FT-IR spectrometer. Only absorption maxima (λ_{max}) of interest are reported and quoted in wavenumbers (cm^{-1}). Low and high resolution mass spectra were recorded by staff at the University of Manchester. Electrospray (ES) spectra were recorded on a Waters Platform II and high resolution mass spectra (HRMS) were recorded on a Thermo Finnigan MAT95XP and are accurate to ±0.001 Da. Melting points were determined on a GallenKamp apparatus and are uncorrected. Optical rotation measurements were taken on an AA-100 polarimeter at 20 °C with the solvent and concentration (g/100 mL) stated. Circular Dichroism (CD) measurements were performed at 20 °C on a JASCO J-815 spectropolarimeter, using a 1 mm cell with the solvent and concentration stated, where applicable. Analytical HPLC analysis was performed on a Hewlett-Packard series 1050 system, using the column and eluent stated, with UV detection at 254, 230 and 210 nm.

Experimental Procedures

Methods for the synthesis of N_3Aib_4OH, $HAib_4OtBu$, Z-GlyAib_4O^tBu,[1] Z-Aib*Aib_4OH and Z-L-AlaNH^{t}Bu[2] have been previously reported.

Abbreviations

DIPEA, N,N-diisopropylethylamine; EDC, N-(3-dimethylaminopropyl)-N′-ethylcarbodiimide; HOAt, 1-Hydroxy-7-azabenzotriazole**;** HOBt, 1-hydroxybenzotriazole; IBCF, isobutyl chloroformate; NMM, 4-methylmorpholine; Z, carboxybenzyl.

5-Bromo-7-nitroindoline (Bni) 1

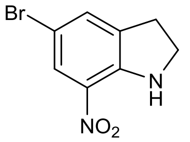

5-Bromo-7-nitroindoline **1** was prepared according to the method reported by Hiroshi *et al.*.[3] The pure product (757 mg, 89%) was isolated as an orange solid by column chromatography (4:1 PE:EtOAc). **^{1}H-NMR** (400 MHz, $CDCl_3$) δ_H 7.95 (1H, m, *o*-NO_2-ArCH), 7.28 (1H, m, *p*-NO_2-ArCH), 6.80 (1H, br s, NH), 3.91 (2H, m, C*H*$_2$-NH), 3.20 (2H, m, CH_2-Ar). **^{13}C-NMR** (101 MHz, $CDCl_3$) δ_C 148.1 (ArC), 136.0 (ArC), 132.6 (*p*-NO_2-ArCH), 129.1 (ArC), 124.4 (*o*-NO_2-ArCH), 107.1 (ArC), 46.9 (CH_2-NH), 28.0 (CH_2-Ar). **IR** (neat) ν_{max}/cm^{-1} = 3377, 3088, 2890, 2362, 1624, 1585, 1508, 1485, 1434, 1412. **HRMS** (ES$^+$, MeOH) Calc. for $C_8H_7N_2O_2{}^{79}BrNa$ ([M+Na]$^+$) 264.9589, found 264.9593; Calc. for $C_8H_7N_2O_2{}^{81}BrNa$ ([M+Na]$^+$) 266.9569, found 266.9572. **Mp** 131-133 °C.

Fmoc-L-AlaBni

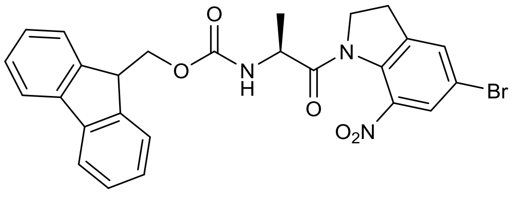

1 (243 mg, 1 mmol) and Fmoc-L-Ala-OH (311 mg, 1 mmol) were dissolved in toluene and thionyl chloride (0.15 mL, 2 mmol) was added dropwise and the reaction stirred at 70 °C for 16 h. EtOAc (20 mL) was added and the organic phase washed with HCl (1 M, 2 x 10 mL), $NaHCO_3$ (sat., 2 x 10 mL), brine (10 mL), dried ($MgSO_4$), filtered and concentrated. The crude residue was purified by column chromatography (1:9 to 1:1 EtOAc:PE) to give the pure product (463 mg, 86%) as a yellow solid. **^{1}H-NMR** (400 MHz, $CDCl_3$) δ_H 7.84 (1H, s, *o*-NO_2-ArCH), 7.76 (2H, d, *J*=7.5, ArCH x2 (Fmoc)), 7.58 (3H, m, *p*-NO_2-ArCH and ArCH x2 (Fmoc)), 7.40 (2H, ddd, *J* 7.5, 7.5, 2.5, ArCH x2 (Fmoc)), 7.31 (2H, m, ArCH x2 (Fmoc)), 5.61 (1H, d, *J*=8.0, NH), 4.74 (1H, dq, *J*= 7.0, 7.0, C*H*-CH_3), 4.58 (1H, ddd, *J* 10.0, 10.0, 3.5, C*H*$_2$-CH_2N), 4.38 (1H, dd, *J*=10.5, 7.5, H^A of ABX system, C*H*$_2$-CH), 4.34 (1H, dd, *J*=10.5, 7.5, H^B of ABX system, C*H*$_2$-CH), 4.22 (2H, m, C*H*-CH_2 and CH_2N), 3.38 (1H, ddd, *J*=17.5, 9.5, 9.5, CH_2-Ar), 3.18 (1H, ddd, 16.5, 9.5, 3.5, CH_2-Ar), 1.50 (3H, d, *J*=7.0, CH_3). **^{13}C-NMR** (101 MHz, $CDCl_3$) δ_C 171.6 (CO), 155.9 (CO (Fmoc)), 143.7 (ArC), 141.2 (ArC), 140.8 (ArC), 138.7 (ArC), 133.8 (ArC), 131.9 (*p*-NO_2-ArCH), 127.7 (ArCH (Fmoc)), 127.1 (ArCH (Fmoc)), 125.5 (*o*-NO_2-ArCH), 125.1 (ArCH (Fmoc)), 120.0 (ArCH

(Fmoc)), 117.0 (ArC), 67.2 (*C*H_2-CH), 49.8 (CH_2N), 49.1 (*C*H-CH_3), 47.0 (*C*H-CH_2), 29.1 (CH_2Ar), 18.3 (CH_3). **IR** (neat) ν_{max}/cm^{-1} = 3312, 2951, 2360, 2341, 1679, 1537, 1460, 1450. **HRMS** (ES^+, MeOH) Calc. for $C_{26}H_{26}N_4O_5{}^{79}Br$ ($[M+NH_4]^+$) 553.1081, found 553.1074; Calc. for $C_{26}H_{26}N_4O_5{}^{81}Br$ ($[M+NH_4]^+$) 555.1061, found 555.1053. **Mp** 115-117 °C. $[\alpha]_D^{20}$ −92.0 (*c* 0.5, CH_2Cl_2).

H-L-AlaBni 2

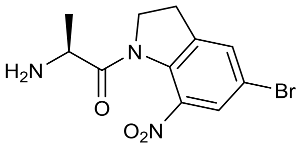

Fmoc-L-AlaBni (391 mg, 0.73 mmol) was dissolved in CH_2Cl_2 (4 mL) and diethylamine (1 mL) was added. The solution was stirred for 5 h and then concentrated. The crude residue was purified by column chromatography (2-10% MeOH in CH_2Cl_2) to give the pure product (236 mg, 91%) as a yellow waxy solid. **^{1}H-NMR** (400 MHz, $CDCl_3$) δ_H 7.80 (1H, s, *o*-NO_2-ArCH), 7.56 (1H, s, *p*-NO_2-ArCH), 4.37 (1H, ddd, *J* 10.0, 10.0, 5.0, CH_2N), 4.20 (1H, m, CH_2N), 3.84 (1H, q, *J*=7.0, C*H*-CH_3), 3.32 (1H, ddd, *J* 17.5, 9.0, 9.0, CH_2Ar), 3.18 (1H, ddd, *J*=9.5, 9.5, 5.0, CH_2Ar), 1.38 (3H, d, *J*=7.0, CH_3). **^{13}C-NMR** (126 MHz, $CDCl_3$) δ_C 174.7 (CO), 140.8 (ArC), 138.5 (ArCH), 134.1 (ArC), 131.7 (*p*-NO_2-ArCH), 125.4 (*o*-NO_2-ArCH), 116.5 (ArC), 49.7 (CH), 49.3 (CH_2N), 29.1 (CH_2Ar), 20.8 (CH_3). The enantiopurity of the product was checked by coupling to Z-PheOH (d.r. ≥95:5 by ^{1}H-NMR, see below).

Z-L-Phe-L-AlaBni

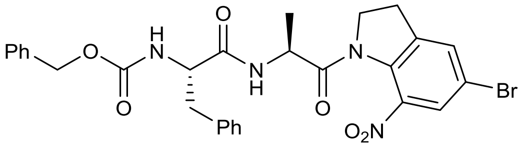

Z-L-PheOH (45 mg, 0.15 mmol) and HOBt hydrate (28 mg, 0.16 mmol) were dissolved in CH_2Cl_2 (5 mL). EDC hydrochloride (161 mg, 0.84 mmol) and DIPEA (44 μL, 0.25 mmol) were added and the reaction mixture stirred until it was homogenous. Then, **2** (31 mg, 0.1 mmol) was added and the reaction mixture was stirred for 48 h. EtOAc (10 mL) was added and the organic phase washed with $KHSO_4$ (5%, 2 x 5 mL), $NaHCO_3$ (sat., 2 x 5 mL), brine (5 mL), dried ($MgSO_4$), filtered and concentrated. The crude residue was purified by column chromatography (1:3 to 1:0 EtOAc:PE) to give the pure product (41 mg, 71%) as a yellow solid. **^{1}H-NMR** (400 MHz, $CDCl_3$) δ_H 7.83 (1H, s, *o*-NO_2-ArCH), 7.58 (1H, s, *p*-NO_2-ArCH), 7.14 – 7.34 (10H, m, ArCH x10), 6.75 (1H, br d, *J*=6.5, NH(Ala)), 5.16 (1H, br d, *J*=7.5, NH (Phe)), 5.04 (2H, s, CH_2O), 4.89 (1H, dq, *J*=7.0, 7.0, C*H*-CH_3), 4.51 (1H, ddd, *J*=10.0, 10.0, 3.5, CH_2N), 4.46 (1H, br dd, *J*=7.0, 7.0, C*H*-CH_2), 4.18 (1H, ddd, *J*=9.5, 9.5, 9.5, CH_2N), 3.34 (1H, m, CH_2Ar), 3.15 (1H, ddd, *J*=9.5, 9.5, 4.0, CH_2Ar), 3.08 (2H, d, *J*=6.0, C*H_2*-CH (Phe)), 1.40 (3H, d, *J*=6.5, CH_3). **^{13}C-NMR** (101 MHz, $CDCl_3$) δ_C 170.82 (CO), 170.75 (CO), 155.8 (CO (Cbz)), 140.8 (ArC), 138.9 (ArC), 136.0 (ArC), 135.9 (ArC), 133.8 (ArC), 131.9 (*p*-NO_2-ArCH), 129.3 (ArCH), 128.7 (ArCH), 128.5 (ArCH), 128.2 (ArCH), 128.0 (ArCH), 127.1 (ArCH), 125.4 (*o*-NO_2-ArCH), 116.9 (ArC), 67.1 (CH_2O), 55.8 (*C*H-CH_2 (Phe)), 49.8 (CH_2N), 47.5 (*C*H-CH_3 (Ala)), 38.0 (*C*H_2-CH (Phe)), 29.1 (CH_2Ar), 17.8 (CH_3). **IR** (neat) ν_{max}/cm^{-1} = 3304, 3064, 2360, 1683, 1638, 1563, 1535, 1497, 1459. **HRMS** (ES^+, MeOH) Calc. for $C_{28}H_{31}N_5O_6{}^{79}Br$ ($[M+NH_4]^+$) 612.1452, found 612.1440; Calc. for $C_{28}H_{31}N_5O_6{}^{81}Br$ ($[M+NH_4]^+$) 614.1432, found 614.1421. **Mp** 96-98 °C. $[\alpha]_D^{20}$ −136.0 (*c* 0.5, CH_2Cl_2).

Z-(*R*)-Aib*-Aib_4-L-AlaBni 4

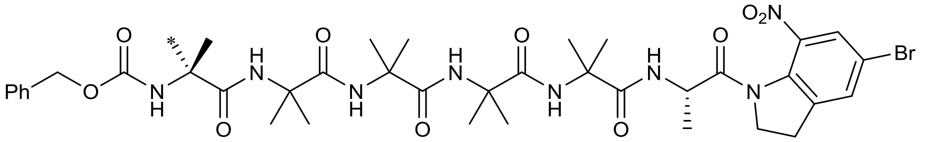

Z-(*R*)-Aib*-Aib_4-OH **3** (15 mg, 0.026 mmol) and HOBt hydrate (6 mg, 0.034 mmol) were dissolved in CH_2Cl_2 (2 mL). EDC hydrochloride (5.5 mg, 0.029 mmol) and triethylamine (9 μL, 0.065 mmol) were added and the reaction mixture stirred until it was homogenous. Then, **2** (20 mg, 0.065 mmol) was added and the reaction mixture was stirred for 48 h. EtOAc (10 mL) was added and the organic phase washed with $KHSO_4$ (5%, 2 x 3 mL), $NaHCO_3$ (sat., 2 x 3 mL), brine (3 mL), dried ($MgSO_4$), filtered and concentrated. The crude residue was purified by column chromatography (0.5-5% MeOH in CH_2Cl_2) to give the pure peptide (13 mg, 57%) as a yellow solid. **^{1}H-NMR** (400 MHz, $CDCl_3$) δ_H 7.76 (1H, s, *o*-NO_2-ArCH), 7.72 (1H, br d, *J*=5.0, N*H*-CH), 7.50 (2H, m, *p*-NO_2-ArCH and NH), 7.48 (1H, br s, NH), 7.43 (1H, br s, NH), 7.35 (5H, m, ArCH x5), 6.54 (1H, br s, NH), 5.73 (1H, br s, NH), 5.12 (1H, d, *J*=12.5, CH_2O, H^A of AB system), 5.05 (1H, d, *J*=12.5, CH_2O, H^B of AB system), 4.96 (1H, ddd, *J*=10.0, 10.0, 4.0, CH_2N), 4.66 (1H, dq, *J*=6.5, 6.5, $^{\alpha}$CH), 4.19 (1H, ddd, *J*=9.5, 9.5, 9.5, CH_2N), 3.41 (1H, m, CH_2Ar), 3.08 (1H, ddd, *J*=10.0, 10.0, 4.5, CH_2Ar), 1.52 (3H, s, CH_3), 1.51 (3H, d, *J*= 7.0, C*H*$_3$-CH), 1.49 (3H, s, CH_3), 1.47 (3H, s, CH_3), 1.46 (0.7H, d, *J*=129.0, *CH_3 minor), 1.46 (2.3H, m, CH_3 major), 1.43 (3H, s, CH_3), 1.42 (2.3H, d, *J* 129.5, *CH_3 major), 1.42 (3.7H, m, CH_3 and CH_3 minor), 1.40 (3H, s, CH_3), 1.34 (3H, s, CH_3), 1.24 (3H, s, CH_3). **^{13}C-NMR** (101 MHz, $CDCl_3$) δ_C 176.0 (CO), 175.1 (CO), 174.7 (CO), 174.1 (CO), 173.8 (CO), 172.2 (CO), 155.8 (CO (Cbz)), 140.6 (ArC), 139.8 (ArC), 136.1 (ArC), 135.0 (ArC), 131.6 (*p*-NO_2-ArCH), 128.6 (ArCH), 128.5 (ArCH), 127.9 (ArCH), 125.1 (*o*-NO_2-ArCH), 115.7 (ArC), 67.2 (CH_2O), 57.0 (d, *J*=36.5, $^{\alpha}$C-*CH_3), 56.70 ($^{\alpha}$C), 56.67 ($^{\alpha}$C), 56.5 ($^{\alpha}$C), 56.4 ($^{\alpha}$C), 50.0 (CH_2N), 48.5 ($^{\alpha}$CH), 29.2 (CH_2Ar), 26.5 (CH_3), 25.8 (*CH_3 major), 25.5 – 26.0 (CH_3 x2), 25.0 (CH_3), 24.8 (CH_3), 24.1 (*CH_3 minor), 23.8 – 24.3 (CH_3 x2), 23.2 (CH_3), 16.6 (*C*H_3-CH). **IR** (neat) ν_{max}/cm^{-1} =3308, 2984, 2938, 1658, 1530, 1457. **HRMS** (ES$^+$, MeOH) Calc. for $C_{38}{}^{13}CH_{57}N_9O_{10}{}^{79}Br$ ($[M+NH_4]^+$) 891.3440, found 891.3434; Calc. for $C_{38}{}^{13}CH_{57}N_9O_{10}{}^{81}Br$ ($[M+NH_4]^+$) 893.3419, found 894.3417. **Mp** 141-143 °C. $[\boldsymbol{\alpha}]_{\boldsymbol{D}}^{\mathbf{20}}$ −236.0 (*c* 0.5, CH_2Cl_2).

Z-Aib*-Aib_4-L-AlaNH^{i}Pr 5

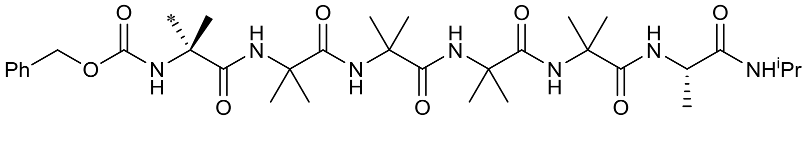

Z-Aib*-Aib_4-L-AlaBni **4** (6.5 mg, 0.0074 mmol) and $^iPrNH_2$ (3.2 μL, 0.037 mmol) were dissolved in THF-d_8 (1.5 mL) and the solution irradiated at 360 nm (Atlas Photonics LUMOS 43 UV-LED) for 1 h. The solution was concentrated *in vacuo* and the crude residue purified by column chromatography (1-5% MeOH in CH_2Cl_2) to give the pure peptide (5 mg, 97%) as a white solid. **^{1}H-NMR** (400 MHz, THF-d_8) δ_H 7.93 (1H, br s, NH), 7.77 (1H, br s, NH), 7.71 (1H, br s, NH), 7.63 (1H, br s, NH), 7.55 (1H, br d, *J*=8.0, N*H*-CH), 7.33 (6H, m, ArCH x5 and NH), 7.13 (1H, d, *J*=7.5, N*H*-CH), 5.20 (1H, d, *J*=12.5, CH_2O, H^A of AB system), 5.05 (1H, d, *J*=12.5, CH_2O, H^B of AB system), 4.14 (1H, dq, *J*= 7.5. 7.5, $^{\alpha}$CH), 3.93 (1H, m, CH), 1.49 (3H, s, CH_3), 1.46 (3H, s, CH_3), 1.46 (2.3H, d, *J*=128.5, *CH_3 major), 1.46 (0.7H, m, CH_3 minor), 1.45 (3H, s, CH_3), 1.44 (3H, s, CH_3), 1.43 (6H, s, CH_3 x2), 1.41 (3H, s, CH_3), 1.39 (2.3H, d,

J=4.0, CH_3 major), 1.39 (0.7H, d, J=129.0, *CH_3 minor), 1.36 (3H, d, J=7.5, CH_3-CH), 1.25 (3H, s, CH_3), 1.13 (3H, d, J= 6.5, CH_3-CH), 1.11 (3H, d, J=6.5, CH_3-CH). **^{13}C-NMR** (101 MHz, THF-d_8) δ_C 176.6 (CO), 176.34 (CO), 176.26 (CO), 175.9 (CO), 174.7 (CO), 172.3 (CO), 157.6 (CO (Cbz)), 138.2 (ArC), 129.5 (ArCH), 129.14 (ArCH), 129.07 (ArCH), 67.6 (CH_2O), 57.8 ($^{\alpha}$C), 57.7 ($^{\alpha}$C), 57.59 ($^{\alpha}$C), 57.57 (d, J=38.5, $^{\alpha}C$-*CH_3), 57.5 ($^{\alpha}$C), 50.6 ($^{\alpha}$CH), 41.8 (CH), 28.2 (CH_3), 27.6 (CH_3), 27.4 (CH_3), 27.2 (CH_3), 26.6 (*CH_3 minor), 24.1 (*CH_3 major), 23.9 (CH_3), 23.8 (CH_3), 23.5 (CH_3), 23.3 (CH_3), 23.2 (CH_3-CH), 22.8 (CH_3-CH), 17.9 (CH_3-CH(Ala)). **IR** (neat) ν_{max}/cm^{-1} = 3298, 2982, 2937, 1703, 1651, 1528, 1455. **HRMS** (ES$^+$, MeOH) Calc. for $C_{33}{}^{13}CH_{56}N_7O_8$ ([M+H]$^+$) 691.4218, found 691.4209. **Mp** 130-132 °C. $[\alpha]_D^{20}$ = +32.0 (c 0.5, CH_2Cl_2).

Z-L-Ala-NH-(CH_2)$_2$-N((CH_2)$_2$NHBoc)$_2$

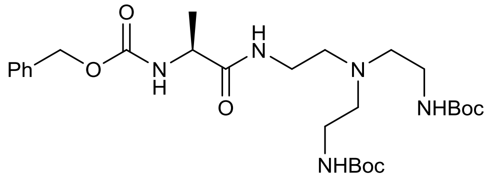

Di-*tert*-butyl(((2-aminoethyl)azanediyl)bis(ethane-2,1-diyl))dicarbamate was prepared according to a modified procedure by Ramdahl.[4] Di-*tert*-butyl dicarbonate (469 mg, 2.15 mmol) in CH_2Cl_2 (1 mL) was added dropwise to a solution of imidazole in CH_2Cl_2 (1 mL) and the resulting solution stirred for 1 h. This solution was then washed with H_2O (2 x 2 mL) and the organic phase dried (Na_2SO_4), filtered and concentrated. The resulting residue was dissolved in toluene (1 mL) with tris(2-aminoethyl)amine **6** (0.15 mL, 1 mmol) and the solution heated to 60 °C for 2.5 h. After cooling to room temperature, the toluene was removed *in vacuo* and CH_2Cl_2 (10 mL) was added. The organic phase was washed with H_2O (4 x 5 mL) and was then dried (Na_2SO_4), filtered and concentrated to give the crude amine (244 mg, 70%). A portion of this (94 mg, 0.27 mmol) was then dissolved with Z-L-Ala-OH (56 mg, 0.25 mmol), EDC hydrochloride (48 mg, 0.25 mmol), HOAt hydrate (34 mg, 0.25 mmol) and NMM (82 μL, 0.75 mmol) in CH_2Cl_2 (2 mL) and the resulting mixture stirred for 16 h. EtOAc (9 mL) was added and the organic phase washed with H_2O (2 x 3 mL), $NaHCO_3$ (sat., 2 x 3 mL), NH_4Cl (sat., 2 x 3 mL), brine (3 mL), dried (Na_2SO_4), filtered and concentrated. The crude residue was purified by column chromatography (1-5% MeOH in CH_2Cl_2) to give the pure product (108 mg, 78%) as a colourless oil. **^{1}H-NMR** (400 MHz, $CDCl_3$) δ_H 7.32 (6H, m, ArCH x5 and NH), 5.71 (1H, br s, NH), 5.31 (2H, br s, NH x2), 5.11 (2H, m, CH_2O), 4.34 (1H, m, CH), 3.26 (2H, m, CH_2-NH), 3.10 (4H, m, CH_2-NHBoc x2), 2.55 (2H, m, CH_2N), 2.49 (4H, m, CH_2N x2), 1.42 (21H, m, C(CH_3)$_3$ x2 and CH_3). **^{13}C-NMR** (101 MHz, $CDCl_3$) δ_C 172.5 (CO), 156.5 (CO (Cbz)), 136.2 (ArC), 128.4 (ArCH), 128.1 (ArCH), 128.0 (ArCH), 79.4 (CMe_3), 66.8 (CH_2O), 55.0 (CH_2N), 54.1 (CH_2N), 50.3 (CH), 39.0 (CH_2-NHBoc), 37.5 (CH_2NH), 28.4 (C(CH_3)$_3$), 18.9 (CH_3). **IR** (neat) ν_{max}/cm^{-1} = 3316, 2977, 1689, 1665, 1515, 1454. **HRMS** (ES$^+$, MeOH) Calc. for $C_{27}{}^{13}CH_{45}N_5O_7Na$ ([M+Na]$^+$) 574.3217, found 574.3193. $[\alpha]_D^{20}$ = −17.6 (c 0.5, CH_2Cl_2).

Z-(*R*)-Aib*-Aib$_4$-L-AlaNH-$(CH_2)_2$-N($(CH_2)_2$NHBoc)$_2$

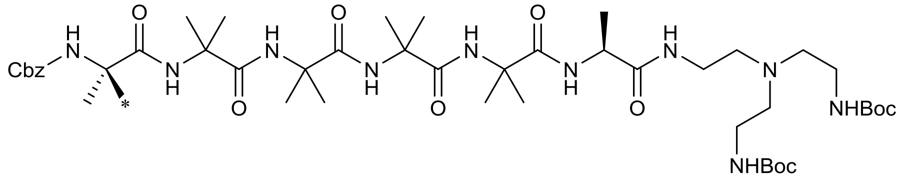

Z-(*R*)-Aib*-Aib$_4$-OH **3** (97 mg, 0.17 mmol) and HOBt hydrate (38 mg, 0.22 mmol) were dissolved in CH_2Cl_2 (5 mL). EDC hydrochloride (35 mg, 0.19 mmol) and triethylamine (60 μL, 0.42 mmol) were added and the reaction mixture stirred until it was homogenous. Then, **7** (prepared by the quantitative hydrogenation of Z-L-Ala-NH-$(CH_2)_2$-N($(CH_2)_2$NHBoc)$_2$, 79 mg, 0.19 mmol) was added and the reaction mixture was stirred for 48 h. CH_2Cl_2 (10 mL) was added and the organic phase washed with $KHSO_4$ (5%, 2 x 3 mL), $NaHCO_3$ (sat., 2 x 3 mL), brine (3 mL), dried (Na_2SO_4), filtered and concentrated. The crude residue was purified by column chromatography (1-8% MeOH in CH_2Cl_2) to give the pure peptide (153 mg, 92%) as a white solid. **^{1}H-NMR** (400 MHz, $CDCl_3$) δ_H 7.70 (1H, br s, NH), 7.62 (2H, m, NH and N*H*-CH), 7.54 (1H, br s, NH), 7.51 (1H, br s, NH), 7.35 (5H, m, ArCH x5), 6.57 (1H, br s, NH), 5.76 (1H, br s, NH), 5.70 (2H, br s, NH x2), 5.21 (1H, d, *J*=12.5, CH_2O, H^A of AB system), 5.04 (1H, d, *J*=12.5, CH_2O, H^B of AB system), 4.45 (1H, dq, *J*=7.5, 7.5, CH), 3.32 (2H, m, C*H*$_2$-NH), 3.15 (4H, m, C*H*$_2$-NHBoc x2), 2.63 (6H, m, CH_2-N x3), 1.56 (3H, s, CH_3), 1.51 (2.2H, d, *J*=129.0, *CH_3 major), 1.51 (0.8H, m, CH_3 minor), 1.51 (3H, s, CH_3), 1.49 (3H, s, CH_3), 1.48 (3H, d, *J*=7.5, C*H*$_3$-CH), 1.46 (3H, s, CH_3), 1.45 (0.8H, d, *J*=129.0, *CH_3 minor), 1.45 (2.2H, m, CH_3 major), 1.44 (21H, s, CH_3 and $C(CH_3)_3$ x2), 1.42 (3H, s, CH_3), 1.39 (3H, s, CH_3), 1.20 (3H, s, CH_3). **^{13}C-NMR** (101 MHz, $CDCl_3$) δ_C 176.2 (CO), 175.6 (CO), 175.3 (CO), 174.23 (CO), 174.16 (CO), 173.4 (CO), 156.5 (CO), 156.0 (CO (Cbz)), 136.1 (ArC), 128.7 (ArCH), 128.6 (ArCH), 128.0 (ArCH), 78.6 (CMe_3), 67.4 (CH_2O), 57.2 (d, *J*=39.5, $^{\alpha}C$-*CH_3), 57.0 ($^{\alpha}$C), 56.7 ($^{\alpha}$C), 56.6 ($^{\alpha}$C), 56.4 ($^{\alpha}$C), 54.1 (CH_2N), 53.9 (CH_2N), 49.5 (CH), 38.7 (CH_2NHBoc), 38.0 (CH_2NH), 28.5 (C(*C*H$_3$)$_3$), 27.5 (CH_3), 27.03 (CH_3), 26.98 (CH_3), 26.8 (*CH_3 minor and CH_3), 23.2 (*CH_3 major and CH_3), 22.8 (CH_3), 22.72 (CH_3), 22.67 (CH_3), 17.1 (CH_3). **IR** (neat) ν_{max}/cm^{-1} = 3299, 2980, 2936, 1702, 1652, 1527, 1455. **HRMS** (ES$^+$, MeOH) Calc. for $C_{46}{}^{13}CH_{81}N_{10}O_{12}$ ([M+H]$^+$) 978.6069, found 978.6036. **Mp** 108-110 °C. $[\alpha]_D^{20}$ = +28.8 (*c* 0.5, CH_2Cl_2).

Z-(*R*)-Aib*-Aib$_4$-L-AlaNH-$(CH_2)_2$-N($(CH_2)_2NH_2$)$_2$ 8

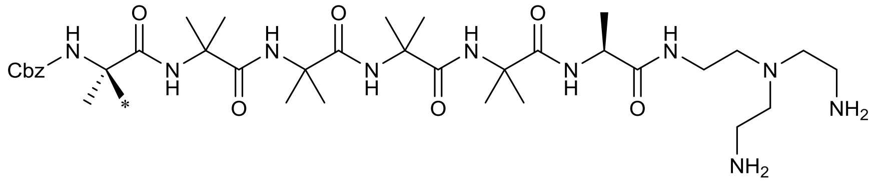

Z-(*R*)-Aib*-Aib$_4$-L-AlaNH-$(CH_2)_2$-N($(CH_2)_2$NHBoc)$_2$ (145 mg, 0.15 mmol) was dissolved in CH_2Cl_2 (5 mL), then HCl (2 M in Et_2O, 3 mmol) was added dropwise and the resulting solution stirred for 24 h. The solvent was removed *in vacuo* and Et_2O was then added. This process was repeated twice more and the resulting precipitate was filtered and dried *in vacuo*. The hydrochloride salts were converted to the free amines by addition of $NaHCO_3$ (sat., 5 mL) and extraction with $CHCl_3$:IPA (3:1, 2 x 10 mL), giving the pure peptide (118 mg, 94%) as a white solid. **^{1}H-NMR** (400 MHz, CD_3OD) δ_H 8.01 (1H, br s, NH), 7.74 (1H, br s, NH), 7.34 (5H, m, ArCH x5), 5.20 (1H, d, *J*=13.0, CH_2O, H^A of AB system), 5.07 (1H, d, *J*=13.0, H^B of AB system), 4.23 (1H, q, *J*=7.5, CH), 3.81 (1H, m, H^A of C*H*$_2$NH), 3.12-3.39 (11H, m, H^B

of CH_2-NH, CH_2-NH_2 x2 and CH_2-N x3), 1.52 (3H, s, CH_3), 1.50 (3H, s, CH_3), 1.49 (3H, s, CH_3), 1.48 (3H, m, CH_3-CH), 1.45 (3H, s, CH_3), 1.44 (0.8H, d, *J*=3.5, CH_3 minor), 1.44 (2.2H, d, *J*=128.0, *CH_3 major), 1.40 (0.8H, d, *J*=129.0, *CH_3 minor), 1.40 (2.2H, m, CH_3 major), 1.39 (3H, s, CH_3), 1.37 (3H, s, CH_3), 1.35 (3H, s, CH_3), 1.28 (3H, s, CH_3). **^{13}C-NMR** (101 MHz, CD_3OD) δ_C 178.6 (CO), 178.4 (CO), 178.1 (CO), 177.3 (CO), 177.1 (CO), 176.6 (CO), 158.0 (CO (Cbz)), 138.7 (ArC), 129.6 (ArCH), 129.0 (ArCH), 128.5 (ArCH), 67.6 (CH_2O), 57.9 ($^{\alpha}$C), 57.7 ($^{\alpha}$C), 57.6 (d, *J*=39.0, $^{\alpha}$*C*-*CH_3), 57.6 ($^{\alpha}$C), 57.5 ($^{\alpha}$C), 54.5 (CH_2N), 52.5 (CH_2N), 51.8 (CH), 36.9 (CH_2N), 27.4 (CH_3), 26.9 (CH_3), 26.7 (CH_3), 26.6 (CH_3), 26.1 (*CH_3 minor), 25.2 (CH_3), 24.4 (*CH_3 major), 23.7 (CH_3), 23.7 (CH_3 x2), 17.2 (*C*H_3-CH). **IR** (neat) ν_{max}/cm^{-1} = 3296, 2983, 2937, 2818, 1702, 1650, 1529, 1466, 1454. **HRMS** (ES$^+$, MeOH) Calc. for $C_{36}{}^{13}CH_{65}N_{10}O_8$ ([M+H]$^+$) 778.5020, found 778.4998. **Mp** 102-104 °C. $[\alpha]_D^{20}$ = +24.0 (*c* 0.5, CH_2Cl_2).

<u>Procedure for acid/base switch experiment ([**8**H.Zn]$^{2+}$ ↔ [**8**.Zn]$^+$)</u>

Peptide **8** (5.8 mg, 0.0075 mmol) was dissolved in CD_3OH (500 μL) in an NMR tube such that the [**8**] = 15 mM. Stock solutions of $Zn(ClO_4)_2.6H_2O$ (0.165 M, 1.1 eq. = 50 μL), $Bu_4NOH.30H_2O$ (0.4125 M, 1.1 eq. = 20 μL) and acetic acid (0.825 M, 1.1 = 10 μL) were prepared. Aliquots (0.55, 1.1, 2.2 eq.) of the solution of $Zn(ClO_4)_2.6H_2O$ were added to the NMR tube to monitor the change in shift of the amide protons of **8** by ^{1}H-NMR. An aliquot of base (Bu_4NOH, 1.1 eq.) was added and the change in helical screw-sense preference monitored by ^{13}C-NMR. This process was repeated with acid (AcOH, 1.1 eq.), then base, then acid again (see paper for NMR spectra regions of interest).

N_3Aib$_4$-L-AlaNH^{t}Bu

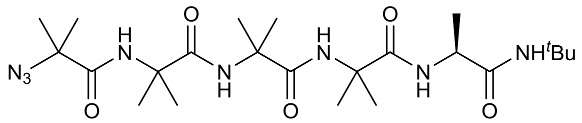

To a stirred solution of N_3Aib$_4$OH (554 mg, 1.44 mmol), HOBt hydrate (265 mg, 1.73 mmol) and EDC hydrochloride (303 mg, 1.59 mmol) in anhydrous CH_2Cl_2 (14 mL) was added DIPEA (630 μL, 3.61 mmol) and the mixture stirred at room temperature for 30 min. H-L-AlaNH^{t}Bu (270 mg, 1.88 mmol; prepared by the quantitative hydrogenolysis of Z-L-AlaNH^{t}Bu) was added in a single portion and the reaction stirred at room temperature for 2 d. The solution was diluted with CH_2Cl_2 (25 mL) and washed with $KHSO_4$ (5%, 2 x 20 mL), $NaHCO_3$ (sat., 2 x 20 mL) and brine (20 mL), then dried ($MgSO_4$) and concentrated *in vacuo*. The crude residue was purified by flash column chromatography (1-5% MeOH in CH_2Cl_2) to give the title compound (696 mg, 95%) as a white solid. **^{1}H-NMR** (400 MHz, $CDCl_3$) δ_H 7.43 (1H, d, *J* = 7.6), 7.26 (1H, s), 6.87 (1H, s), 6.74 (1H, s), 6.13 (1H, s), 4.31 (1H, dq, *J* = 7.6), 1.55 (3H, s), 1.53 (3H, s), 1.51 (3H, s), 1.46 (3H, s), 1.45 (3H, s), 1.43 (6H, s), 1.39 (3H, d, *J* = 7.6), 1.35 (9H, s). **^{13}C-NMR** (100 MHz, $CDCl_3$) δ_C 174.6 (CO), 173.8 (CO), 173.3 (CO), 173.2 (CO), 172.4 (CO), 64.2 ($^{\alpha}$C), 57.3 ($^{\alpha}$C), 57.1 ($^{\alpha}$C), 57.0 ($^{\alpha}$C), 51.2 (CMe_3), 50.1 ($^{\alpha}$CH), 28.9 (3 x CH_3), 27.6 (CH_3), 27.5 (CH_3), 26.5 (CH_3), 24.7 (CH_3), 24.4 (CH_3), 23.2 (CH_3), 23.4 (2 x CH_3), 17.4 (CH_3). **IR** (neat) ν_{max}/cm^{-1} = 3291, 2976, 2936, 2109, 1648, 1529, 1455, 1380, 1361, 1219. **HRMS** (ES$^+$; MeOH) Calc. for $C_{23}H_{43}N_8O_5$ = 511.3356, found 511.3358. **Mp** 221-222 °C. $[\alpha]_D^{20}$ = +14.0 (*c* 1.0, MeOH).

Z-GlyAib$_4$-L-AlaNH^{t}Bu

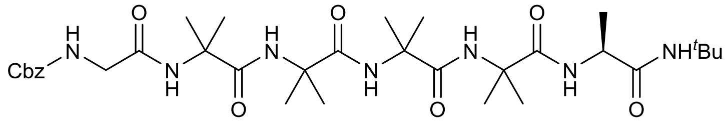

To a stirred solution of Z-GlyOH (95 mg, 0.46 mmol) and NMM (50 mL, 0.46 mmol) in anhydrous THF (2 mL) at –20 °C was added IBCF (63 mL, 0.49 mmol) dropwise and the reaction stirred at –20 °C for 20 min. A solution of HAib$_4$-L-AlaNH^{t}Bu (147 mg, 0.30 mmol; prepared by the quantitative hydrogenolysis of N_3Aib$_4$-L-AlaNH*t*Bu) and NMM (100 mL, 0.91 mmol) in THF (3 mL) was added, the cooling bath removed and the reaction stirred at room temperature for 2 d. The solution was diluted with EtOAc (20 mL) and washed with $KHSO_4$ (5%, 2 x 15 mL), $NaHCO_3$ (sat., 2 x 15 mL) and brine (20 mL), then dried ($MgSO_4$) and concentrated *in vacuo*. The residue was purified by flash column chromatography (1-6% MeOH in CH_2Cl_2) to give the title compound (172 mg, 84%) as a white solid. **^{1}H-NMR** (400 MHz, $CDCl_3$) δ_H 7.76 (1H, d, *J* = 7.5), 7.61 (1H, s), 7.52 (1H, s), 7.38-7.31 (5H, m), 7.27 (1H, s), 7.18 (1H, s), 6.90 (1H, s), 6.54 (1H, t, *J* = 5.0), 5.20 (1H, d, *J* = 12.5, H^A of AB), 5.07 (1H, d, *J* = 12.5, H^B of AB), 4.17 (1H, dq, *J* = 7.5, 7.5), 3.76 (2H, d, *J* = 5.0), 1.56 (3H, s), 1.47-1.43 (15H, m), 1.40 (3H, s), 1.38 (12H, s), 1.31 (3H, s). **^{13}C-NMR** (125 MHz, MeOD) δ_C 175.7 (CO), 175.6 (CO), 175.5 (CO), 174.4 (CO), 173.6 (CO), 170.5 (CO), 157.8 (CO, Cbz), 136.5 (ArC), 128.8 (ArCH), 128.5 (ArCH), 128.0 (ArCH), 67.4 (CH_2, Cbz), 56.94 ($^{\alpha}$C), 56.93 ($^{\alpha}$C), 56.83 ($^{\alpha}$C), 56.79 ($^{\alpha}$C), 51.3 (CMe_3), 51.0 ($^{\alpha}$CH), 46.0 (CH_2, Gly), 28.8 ($(CH_3)_3$), 27.6 (CH_3), 27.0 (CH_3), 26.9 (2 x CH_3), 23.6 (CH_3), 23.3 (CH_3), 23.2 (CH_3), 23.0 (CH_3), 17.5 (CH_3, Ala). **IR** (neat) ν_{max}/cm^{-1} = 3304, 2983, 2937, 1651, 1525, 1455, 1384, 1362, 1267, 1227, 1169. **HRMS** (ES$^+$; MeOH) Calc. for $C_{33}H_{54}N_7O_8$ = 676.4034, found 676.4039. **Mp** 124-126 °C. $[\alpha]_D^{20}$ = +28.9 (*c* 1.0, $CHCl_3$).

General procedure A: synthesis of thiourea catalysts from non-commercially available isothiocyanates

To a vigorously stirred biphasic mixture of primary amine and $CaCO_3$ (3 equiv.) in CH_2Cl_2/H_2O (1:1, 50 mL/mmol) at 0 °C was added thiophosgene dropwise and the mixture stirred at 0 °C for 2 h. The layers were separated and the aqueous layer extracted with CH_2Cl_2 (2 x 10 mL). The combined organics were washed with $NaHCO_3$ (sat., 15 mL) and brine (15 mL), then dried (Na_2SO_4) and concentrated. The residue was purified by flash column chromatography and the corresponding isothiocyanate added as a solution in THF (10 mL/mmol) to a flame-dried RB flask charged with HAib$_4$-L-AlaNH^{t}Bu or HGlyAib$_4$-L-AlaNH^{t}Bu (1.0 equiv.). The reaction was heated at reflux for 3-5 d, the solvents removed and the residue purified directly by flash column chromatography.

General procedure B: synthesis of thiourea catalysts from non-commercially available isothiocyanates

To a stirred solution of primary amine in anhydrous THF (10 mL/mmol) was added sequentially thiophosgene (1.2 equiv.) and DIPEA (2.4 equiv.) and the mixture stirred at room temperature for 2.5 h. The solvents were removed and the residue purified by flash column chromatography. The corresponding isothiocyanate was added as a solution in THF (20 mL/mmol) to a flame-dried RB flask charged with HAib$_4$-L-AlaNH^{t}Bu or HGlyAib$_4$-L-AlaNH^{t}Bu (1.0 equiv.). The reaction was heated at reflux for 3-5 d, the solvents removed and the residue purified directly by flash column chromatography.

General procedure C: synthesis of thiourea catalysts from commercially available isothiocyanates

To a stirred solution of HAib$_4$-L-AlaNH^{t}Bu or HGlyAib$_4$-L-AlaNH^{t}Bu in anhydrous THF (20 mL/mmol) was added isothiocyanate (1.2 equiv.) and the reaction heated at reflux for 3-5 d. The solvents were removed and the residue purified directly by flash column chromatography.

1-(2-methyl-1-(piperidin-1-yl)propan-2-yl)thiourea-Aib$_4$-L-AlaNH^{t}Bu 9a

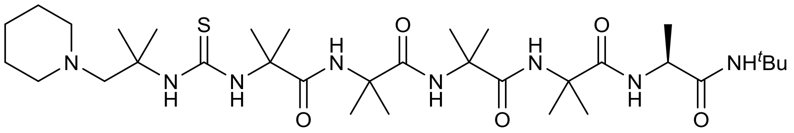

Prepared using general procedure A from 2-methyl-1-(piperidin-1-yl)propan-2-amine (31 mg, 0.20 mmol), $CaCO_3$ (60 mg, 0.60 mmol) and thiophosgene (23 μL, 0.30 mmol). Purification by flash column chromatography (Hexane:EtOAc; 50:50) gave 1-(2-isothiocyanato-2-methylpropyl)piperidine (22 mg, 0.11 mmol) as a yellow oil, which was added as a solution in THF (1.5 mL) to a flame-dried flask charged with H-Aib$_4$-L-AlaNH^{t}Bu (54 mg, 0.011 mmol). The reaction was heated at reflux for 5 d. Purification by flash column chromatography (5-20% MeOH in CH_2Cl_2) gave the title compound (21 mg, 28%) as an off-white solid. **^{1}H-NMR** (400 MHz, CD_2Cl_2) δ_H 10.86 (1H, br s), 8.16 (1H, br s), 7.58 (1H, s), 7.53 (1H, d, *J* = 7.0), 6.80 (1H, s), 6.35 (1H, br s), 6.04 (1H, br s), 4.10 (1H, m), 2.67 (2H, br s), 2.51 (2H, br s), 1.64 (6H, br s), 1.61 (6H, br s), 1.50 (3H, s), 1.44 (3H, s), 1.42 (6H, m), 1.39 (6H, m), 1.35 (6H, m), 1.34 (9H, s). **^{13}C-NMR** (100 MHz, MeOD) δ_C 182.4 (CS), 175.4 (CO), 175.3 (CO), 175.1 (CO), 174.4 (CO), 173.1 (CO), 60.4 (br, CH_2), 58.7 ($^\alpha$C), 57.6 ($^\alpha$C), 57.3 ($^\alpha$C), 57.1 ($^\alpha$C), 56.9 (br, CH_2), 51.2 (CMe_3), 50.8 ($^\alpha$CH), 29.0 ($(CH_3)_3$), 27.7 (CH_3), 27.6 (CH_3), 27.5 (CH_3), 27.3 (CH_3), 25.9 (br, CH_2),

24.6 (br, CH_2), 23.6 (3 x CH_3), 23.4 (CH_3), 17.7 (CH_3). **IR** (neat) ν_{max}/cm^{-1} = 3306, 2976, 2933, 1651, 1526, 1454, 1381, 1361, 1295, 1223, 1169, 1105. **HRMS** (ES^+; MeOH) Calc. for $C_{33}H_{63}N_8O_5S$ = 683.4642, found 683.4622. **Mp** 141-143 °C. $[\alpha]_D^{20}$ = –8.9 (*c* 1.0, MeOH).

1-(2-methyl-1-(piperidin-1-yl)propan-2-yl)thiourea-GlyAib$_4$-L-AlaNH^{t}Bu 9b

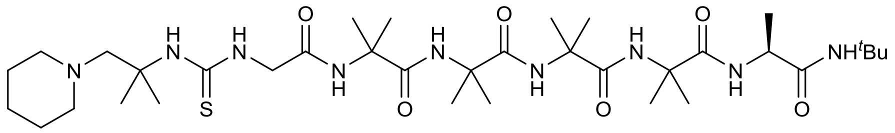

Prepared using general procedure B from 2-methyl-1-(piperidin-1-yl)propan-2-amine (13 mg, 0.080 mmol), thiophosgene (8.0 μL, 0.096 mmol) and DIPEA (33 μL, 0.19 mmol). Purification by flash column chromatography (Hexane:EtOAc, 50:50) gave 1-(2-isothiocyanato-2-methylpropyl)piperidine as a yellow oil (9.0 mg, 0.046 mmol), which was added as a solution in THF (1 mL) to a flame-dried flask charged with HGlyAib$_4$-L-AlaNH^{t}Bu (25 mg, 0.046 mmol). The reaction was heated at reflux for 3 d. Purification by flash column chromatography (5-20% MeOH in CH_2Cl_2) gave the title compound (18 mg, 54%) as an off-white solid. **^{1}H-NMR** (500 MHz, MeOD-d$_4$) δ_H 7.88 (2H, s), 7.79 (1H, s), 7.72 (1H, s), 7.10 (1H, s), 4.35 (1H, d, *J* = 16.0, H^A of AB), 4.10 (1H, m, $^\alpha$CH), 4.02 (1H, d, *J* = 16.0, H^B of AB), 2.62 (4H, br s), 2.49 (2H, br s), 1.57 (6H, br s), 1.50 (3H, s), 1.47-1.42 (24H, m), 1.38-1.35 (15H, m). **^{13}C-NMR** (125 MHz, MeOD) δ_C 184.2 (CS), 177.5 (CO), 177.4 (CO) 177.3 (CO), 176.7 (CO), 174.8 (CO), 171.5 (CO), 70.9 (br, CH_2), 58.9 (br, CH_2), 57.9 (C), 57.8 (C), 57.7 (C), 57.6 (C), 57.4(C), 52.3 (CMe$_3$), 51.9 ($^\alpha$CH), 48.4 (CH_2, Gly), 27.6 (br, CH_2), 27.3 (CH_3), 27.1 (CH_3), 26.9 (CH_3), 26.5 (CH_3), 26.0 (CH_3), 24.4 (br, CH_2), 24.1 (CH_3), 23.9 (CH_3), 23.84 (CH_3), 23.78 (CH_3), 17.7 (CH_3).

Synthesis of 1-(2-methyl-2-(piperidin-1-yl)propyl)thiourea-Aib$_4$-L-AlaNH^{t}Bu 9c

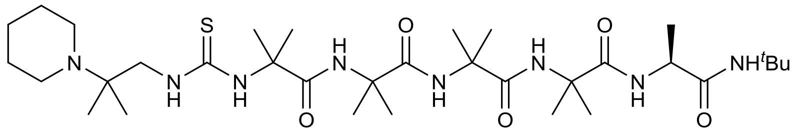

Prepared using general procedure B from 2-methyl-2-(piperidin-1-yl)propan-1-amine (78 mg, 0.50 mmol), thiophosgene (46 μL, 0.60 mmol) and DIPEA (209 μL, 1.20 mmol). Purification by flash column chromatography (Hexane:EtOAc, 50:50) gave 1-(1-isothiocyanato-2-methylpropan-2-yl)piperidine (81 mg, 0.41 mmol) as a yellow oil, a portion of which (22 mg, 0.10 mmol) was added as a solution in THF (1.5 mL) to a flame-dried flask charged with HAib$_4$-L-AlaNH^{t}Bu (48 mg, 0.11 mmol). The reaction was heated at reflux for 5 d. Purification by flash column chromatography (5-20% MeOH in CH_2Cl_2) gave the title compound (59 mg, 79%) as an off-white solid. **^{1}H-NMR** (500 MHz, MeOD-d$_4$) δ_H 8.13 (1H, br s), 7.90 (1H, s), 7.85 (1H, d, *J* = 7.0), 7.70 (1H, s), 7.07 (1H, s), 4.11 (1H, dq, *J* = 7.0), 3.68 (2H, br s), 2.70 (3H, br s), 1.72 (3H, br s), 1.66 (3H, s), 1.52 (3H, s), 1.51 (3H, s), 1.48 (3H, s), 1.44-1.40 (15H, m) 1.37 (9H, s), 1.21 (6H, br s); 2 x CH (piperidine) not observed due to line broadening. **^{13}C-NMR** (125 MHz, MeOD) δ_C 182.3 (CS), 177.5 (CO), 177.4 (CO), 177.3 (CO), 174.9 (CO), 59.9 (br, CH_2), 58.1 (2 x $^\alpha$C), 57.9 (2 x $^\alpha$C), 57.8 (C), 52.4 (CMe$_3$), 52.3 (CH_2), 51.9 ($^\alpha$CH), 29.0 ((CH$_3$)$_3$), 28.0 (CH_3), 27.5 (CH_3), 27.3 (CH_3), 26.9 (CH_3), 24.3 (CH_3), 24.0 (CH_3), 23.9 (CH_3), 23.6 (CH_3),

21.4 (br, CH_2), 17.7 (CH_3). **IR** (neat) ν_{max}/cm^{-1} = 3292, 2978, 2934, 1651, 1526, 1454, 1361, 1281, 1224, 1170. **HRMS** (ES^+; MeOH) Calc. for $C_{33}H_{63}N_8O_5S$ = 683.4642, found 683.4639. **Mp** 158-160 °C. $[\alpha]_D^{20}$ = −10.0 (*c* 1.0, MeOH).

1-(2-methyl-2-(piperidin-1-yl)propyl)thiourea-GlyAib$_4$-L-AlaNH^{t}Bu 9d

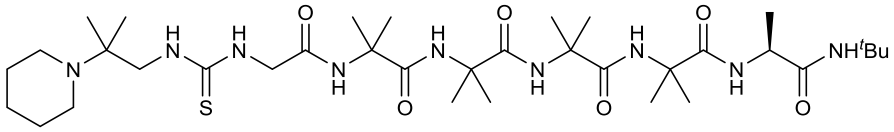

Prepared using general procedure B from 2-methyl-2-(piperidin-1-yl)propan-1-amine (78 mg, 0.50 mmol), thiophosgene (46 μL, 0.60 mmol) and DIPEA (209 μL, 1.20 mmol). Purification by flash column chromatography (Hexane:EtOAc, 50:50) gave 1-(1-isothiocyanato-2-methylpropan-2-yl)piperidine (91 mg, 0.46 mmol) as a yellow oil, a portion of which (22 mg, 0.11 mmol) was added as a solution in THF (1.5 mL) to a flame-dried flask charged with H-GlyAib$_4$-L-AlaNH^{t}Bu (54 mg, 0.10 mmol). The reaction was heated at reflux for 3 d. Purification by flash column chromatography (5-20% MeOH in CH_2Cl_2) gave the title compound (50 mg, 68%) as an off-white solid. **^{1}H-NMR** (500 MHz, MeOD-d$_4$) δ_H 7.89 (1H, d, *J* = 7.0), 7.84 (1H, s), 7.75 (1H, s), 7.10 (1H, s), 4.27 (1H, d, *J* = 17.0, H^A of AB), 4.16 (1H, br s), 4.11 (2H, m, $^{\alpha}$H + H^B of AB), 3.84 (1H, br s), 3.74 (1H, br s), 3.06 (2H, br s), 1.96 (2H, br s), 1.86 (3H, br s), 1.51 (3H, s), 1.47 (3H, s), 1.46 (3H, s), 1.443 (3H, s), 1.440 (3H, s), 1.43-1.42 (12H, m), 1.37 (9H, s); 2 x CH (piperidine) not observed due to line broadening. **^{13}C-NMR** (125 MHz, MeOD) δ_C 183.4 (CS), 177.5 (CO), 177.43 (CO), 177.39 (CO), 177.3 (CO), 176.6 (CO), 174.9 (CO), 58.02 ($^{\alpha}$C), 57.97 ($^{\alpha}$C), 57.9 ($^{\alpha}$C), 57.8 ($^{\alpha}$C), 57.7 (C), 52.42 (CMe_3), 51.9 ($^{\alpha}$CH), 48.2 (CH_2, Gly), 28.7 ($(CH_3)_3$), 27.3 (CH_3), 26.8 (CH_3), 26.6 (CH_3), 26.0 (CH_3), 24.7 (br, CH_2), 23.8 (CH_3), 23.7 (CH_3), 23.6 (CH_3), 23.5 (CH_3), 21.2 (CH_3), 21.0 (CH_3), 17.4 (CH_3); 2 x CH_2 (piperidine) not observed due to line broadening. **IR** (neat) ν_{max}/cm^{-1} = 3295, 2982, 2937, 1651, 1526, 1454, 1383, 1361, 1287, 1226, 1171. **HRMS** (ES^+; MeOH) Calc. for $C_{35}H_{66}N_9O_6S$ = 740.4857, found 740.4839. **Mp** 176-178 °C. $[\alpha]_D^{20}$ = +23.7 (*c* 1.0, MeOH).

1-(2-(piperidin-1-yl)ethyl)thiourea-Aib$_4$-L-AlaNH^{t}Bu 9e

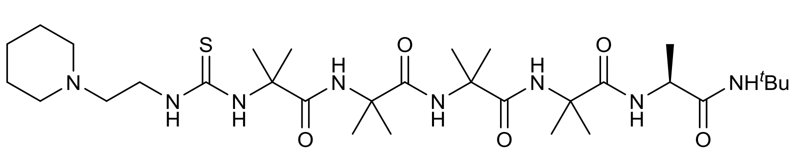

Prepared using general procedure C from HAib$_4$-L-AlaNH^{t}Bu (41 mg, 0.085 mmol) and 2-piperidinoethyl isothiocyanate (17 μL, 0.10 mmol). The reaction was heated at reflux for 5 d. Purification by flash column chromatography (5-20% MeOH in CH_2Cl_2) gave the title compound (42 mg, 76%) as an off-white solid. **^{1}H-NMR** (400 MHz, MeOD-d$_4$) δ_H 8.16 (1H, s), 7.98 (1H, s), 7.91 (1H, d, *J* = 7.0), 7.12 (1H, s), 4.10 (1H, dq, *J* = 7.0, 7.0), 3.90 (1H, br s), 3.83 (1H, br s), 2.98 (6H, br s), 1.79 (4H, br m), 1.66 (3H, s), 1.61 (2H, br s), 1.52 (3H, s), 1.49 (3H, s), 1.48 (3H, s), 1.44-1.41 (12H, m), 1.40 (3H, s), 1.38 (9H, s). **^{13}C-NMR** (100 MHz, MeOD) δ_C 183.2 (CS), 177.5 (2 x CO), 177.3 (CO), 176.4 (CO), 174.9 (CO), 59.9 ($^{\alpha}$C), 58.1 ($^{\alpha}$C), 58.0 (CH_2), 57.92 ($^{\alpha}$C), 57.8 ($^{\alpha}$C), 55.2 (CH_2), 52.4 (CMe_3), 51.9 ($^{\alpha}$CH), 29.0 ($(CH_3)_3$), 28.1 (CH_3), 27.6 (CH_3), 27.3 (CH_3), 27.0 (CH_3), 25.3 (br, CH_2), 24.2 (CH_3), 24.0 (CH_3), 23.8 (CH_3), 23.6 (CH_3), 17.7 (CH_3). **IR** (neat) ν_{max}/cm^{-1} = 3284, 2981, 2936, 1650, 1526, 1454, 1382, 1361,

1293, 1223, 1169. **HRMS** (ES^+; MeOH) Calc. for $C_{31}H_{59}N_8O_5S$ = 655.4329, found 655.4300. **Mp** 169-170 °C. $[\alpha]_D^{20}$ = −17.6 (*c* 1.0, MeOH).

1-(2-(piperidin-1-yl)ethyl)thiourea-GlyAib$_4$-L-AlaNH^{t}Bu 9f

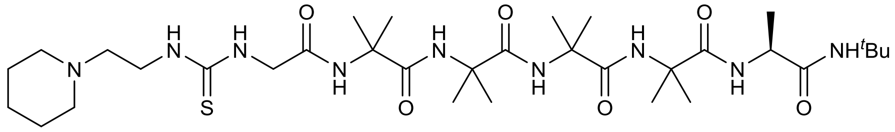

Prepared using general procedure C from HGlyAib$_4$-L-AlaNH^{t}Bu (29 mg, 0.054 mmol) and 2-piperidinoethyl isothiocyanate (11 μL, 0.064 mmol). The reaction was heated at reflux for 3 d. Purification by flash column chromatography (5-20% MeOH in CH_2Cl_2) gave the title compound (32 mg, 84%) as an off-white solid. **^{1}H-NMR** (500 MHz, MeOD-d$_4$) δ_H 7.70 (1H, s), 4.22 (1H, br d, *J* = 16.5, H^A of AB), 4.10 (1H, q, *J* = 7.5, $^\alpha$H), 3.99 (1H, d, *J* = 16.5, H^B of AB), 3.70 (2H, br m), 2.62 (6H, br m), 1.65 (4H, br m), 1.51 (3H, s), 1.48 (3H, s), 1.45-1.42 (18H, m), 1.41 (3H, s), 1.38 (9H, s); 2 x CH (piperidine) not observed due to line broadening. **^{13}C-NMR** (125 MHz, MeOD) δ_C 182.2 (CS), 177.44 (CO), 177.41 (CO), 177.38 (CO), 176.7 (CO), 174.9 (CO), 174.8 (CO), 58.7 (br, CH_2), 57.9 ($^\alpha$C), 57.81 ($^\alpha$C), 57.76 ($^\alpha$C), 57.6 ($^\alpha$C), 55.5 (CH_2), 52.4 (CMe_3), 51.9 ($^\alpha$CH), 48.2 (CH_2, Gly), 29.0 ($(CH_3)_3$), 27.6 (CH_3), 27.0 (CH_3), 26.9 (CH_3), 26.4 (CH_3), 24.8 (CH_2), 24.1 (CH_3), 23.9 (2 x CH_3), 23.8 (CH_3), 17.7 (CH_3). **IR** (neat) ν_{max}/cm^{-1} = 3296, 2982, 2935, 1651, 1526, 1454, 1383, 1361, 1288, 1226, 1117. **HRMS** (ES^+; MeOH) Calc. for $C_{33}H_{62}N_9O_6S$ = 712.4544, found 712.4547. **Mp** 165-166 °C. $[\alpha]_D^{20}$ = 18.3 (*c* 1.0, MeOH).

1-(2-(piperidin-1-yl)ethyl)thiourea-Aib$_4$O^{t}Bu 9g

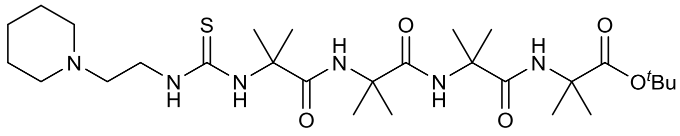

Prepared using general procedure C from H-Aib$_4$O^{t}Bu (41 mg, 0.10 mmol) and 2-piperidinoethyl isothiocyanate (18 μL, 0.11 mmol). The reaction was heated at reflux for 5 d. Purification by flash column chromatography (5-20% MeOH in CH_2Cl_2) gave the title compound (45 mg, 77%) as an off-white solid. **^{1}H-NMR** (500 MHz, MeOD-d$_4$) δ_H 7.84 (1H, s), 3.79 (2H, br s), 2.80 (6H, br s), 1.72 (4H, br s), 1.57 (8H, m), 1.452 (6H, s), 1.446 (6H, s), 1.43 (9H, s), 1.39 (6H, s). **^{13}C-NMR** (125 MHz, MeOD) δ_C 181.8 (br, CS), 175.09 (CO), 174.95 (CO), 174.8 (CO), 174.2 (CO), 80.1 (CMe_3), 58.5 (br, CH_2), 57.1 (br, CH_2), 56.7 ($^\alpha$C), 56.6 ($^\alpha$C), 56.1 ($^\alpha$C), 54.0 ($^\alpha$C), 40.0 (CH_2), 26.8 ($(CH_3)_3$), 24.9 (CH_3), 24.6 (CH_3), 24.0 (CH_3), 23.8 (CH_3), 23.0 (CH_2). **IR** (neat) ν_{max}/cm^{-1} = 3344, 3278, 2981, 2937, 1720, 1671, 1650, 1537, 1512, 1469, 1456, 1381, 1361, 1312, 1277, 1260, 1226, 1101. **HRMS** (ES^+; MeOH) Calc. for $C_{28}H_{53}N_6O_5S$ = 585.3798, found 585.3797, **Mp** 203-205 °C.

1-(2-(piperidin-1-yl)ethyl)thiourea-GlyAib$_4$-L-AlaBni 11

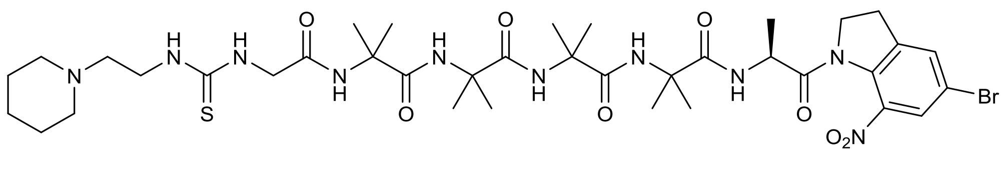

To a stirred solution of H-GlyAib$_4$O^{t}Bu (236 mg, 0.50 mmol) in anhydrous THF (20 mL/mmol) was added 2-piperidinoethyl isothiocyanate (99 μL, 0.60 mmol) and the reaction heated at reflux for 3 d. The solvents were removed and the residue purified directly by flash column chromatography (5-20% MeOH in CH_2Cl_2) giving the corresponding thiourea (264 mg, 0.412 mmol) as an off-white solid. The solid was dissolved in anhydrous CH_2Cl_2 (5 mL), the solution cooled to 0 °C and trifluoroacetic acid (520 μL, 6.96 mmol) added dropwise. The cooling bath was removed and the mixture stirred at room temperature for 2 h. The solvents were removed *in vacuo* and the residual oily solid triturated with Et_2O giving a white solid (261 mg). The carboxylic acid was contaminated with an inseparable unknown impurity (approx. 10% by ^{1}H NMR) and was therefore coupled directly to H-L-AlaBni. To a stirred solution of the carboxylic acid (148 mg, 0.24 mmol), HOBt hydrate (48 mg, 0.31 mmol) and EDC hydrochloride (55 mg, 0.29 mmol) in anhydrous CH_2Cl_2 (3 mL) was added DIPEA (83 μL, 0.48 mmol) and the mixture stirred at room temperature for 30 min. H-L-AlaBni (50 mg, 0.16 mmol) was added in a single portion and the reaction stirred at room temperature for 2 d. The solution was diluted with CH_2Cl_2 (15 mL) and washed with $KHSO_4$ (5%, 2 x 10 mL), $NaHCO_3$ (sat., 2 x 10 mL) and brine (15 mL), then dried ($MgSO_4$) and concentrated *in vacuo*. The residue was purified by flash column chromatography (5-20% MeOH in CH_2Cl_2) giving the title compound (55 mg, 38%) as a yellow solid. **^{1}H-NMR** (500 MHz, MeOD-d$_4$) δ_H 7.85 (1H, d, *J* = 7.0), 7.81 (1H, s, ArH), 7.73 (1H, m, ArH), 4.76 (1H, td, *J* = 10.0, 4.0), 4.70 (1H, q, *J* = 7.0, αCH), 4.23 (1H, dd, *J* = 19.5, 9.5), 4.17 (1H, d, *J* = 17.0, H^A of AB), 4.05 (1H, d, *J* = 17.0, H^B of AB), 3.77 (2H, br s), 3.41 (1H, m), 3.20 (1H, m), 2.76 (6H, br s), 1.69 (4H, br s), 1.48-1.47 (9H, m), 1.45-1.43 (9H, m), 1.42-1.41 (9H, m); 2 x CH (piperidine) not observed due to line broadening. **^{13}C-NMR** (125 MHz, MeOD) δ_C 182.6 (CS), 177.7 (CO), 177.0 (2 x CO), 176.6 (CO), 173.3 (CO), 142.2 (C), 141.8 (C), 135.6 (C), 133.1 (ArCH), 126.0 (ArCH), 117.3 (C), 57.92 ($^{\alpha}$C), 57.85 ($^{\alpha}$C), 57.8 ($^{\alpha}$C), 57.6 ($^{\alpha}$C), 55.4 (CH_2), 51.4 (CH_2Ar), 49.9 ($^{\alpha}$CH), 48.3 (CH_2, Gly), 41.3 (br, CH_2), 30.1 (CH_2N), 27.0 (CH_3), 26.6 (CH_3), 26.2 (CH_3), 25.9 (CH_3), 25.6 (br, CH_2), 24.6 (2 x CH_3), 24.5 (CH_3), 24.4 (CH_3), 16.8 (CH_3). **IR** (neat) ν_{max}/cm^{-1} =3297, 2983, 2935, 1656, 1531, 1457, 1383, 1362, 1276, 1214, 1170. **HRMS** (ES$^+$; MeOH) Calc. for $C_{37}H_{57}N_{10}O_8NaSBr^{79}$ = 903.3163, found 903.3174; Calc. for $C_{37}H_{57}N_{10}O_8NaSBr^{81}$ = 905.3149, found 905.3171. **Mp** 177-179 °C. $[\alpha]_D^{20}$ = −103.2 (*c* 1.0, MeOH).

General procedure D: thiourea-catalysed Michael addition of dialkyl malonate to *trans*-β-nitrostyrene

A flame-dried microwave vial was charged with catalyst (0.010 mmol) and *trans*-β-nitrostyrene (15 mg, 0.10 mmol). The vial was sealed, purged with N_2 and anhydrous CH_2Cl_2 (0.4 mL) added. Dialkyl malonate (0.20 mmol) was added and the reaction stirred at room temperature for 18-21 h. The solvents were removed and the residue purified by column chromatography.

Diethyl 2-(2-nitro-1-phenylethyl)malonate 10 (R = Et)

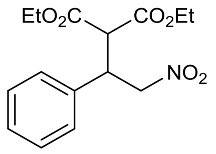

Prepared by general procedure D using **9f** (7.1 mg, 0.010 mmol) and dimethyl malonate (23 μL, 0.20 mmol). The reaction was stirred at room temperature for 18 h (65% conversion by ^{1}H-NMR). Flash column chromatography (Hexane:EtOAc; 80:20) gave the title compound (20 mg, 64%, 50% ee) as a

colourless oil. **¹H-NMR** (500 MHz, $CDCl_3$) δ_H 7.33-7.27 (3H, m), 7.24-7.23 (2H, m), 4.92 (1H, dd, *J* = 13.0, 5.0), 4.86 (1H, dd, *J* = 13.0, 9.0), 4.27-4.18 (3H, m) 4.01 (2H, q, *J* = 7.0), 3.82 (1H, d, *J* = 9.0), 1.26 (3H, t, *J* = 7.0), 1.05 (3H, t, *J* = 7.0). **¹³C-NMR** (125 MHz, $CDCl_3$) δ_C 167.6 (CO), 167.0 (CO), 136.4 (ArC), 129.1 (ArCH), 128.5 (ArCH), 128.2 (ArCH), 77.8 (CH_2), 62.3 (CH_2), 62.0 (CH_2), 55.1 (CH), 43.1 (CH), 14.1 (CH_3), 13.9 (CH_3). **MS** (ES^+, MeOH) m/z = 310 ($[M+H]^+$, 80%), 332 ($[M+Na]^+$, 100%). $[\boldsymbol{\alpha}]_{\boldsymbol{D}}^{\mathbf{20}}$ = −3.2 (*c* 1.0, $CHCl_3$).

The enantiomeric ratio (75:25) was determined by analytical HPLC with a Chiralpak AD-H (5 μm particle size; 250 x 4.6 mm; Daicel) column (hexane:2-propanol; 80:20; 1 mL min^{-1}, 210 nm): t_r (major) = 9.2 min, t_r (minor) = 21.7 min. The major enantiomer was assigned as (*R*) by comparison with literature data: $[\alpha]_D^{20}$ = −5.2 (*c* 1.0, $CHCl_3$), (*R*)-isomer (93% ee).[5]

Dimethyl 2-(2-nitro-1-phenylethyl)malonate (R = Me)

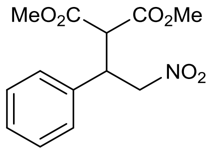

Prepared by general procedure D using **9f** (7.1 mg, 0.010 mmol) and dimethyl malonate (23 μL, 0.20 mmol). The reaction was stirred at room temperature for 21 h (85% conversion by ¹H NMR). Flash column chromatography (Hexane:EtOAc; 80:20) gave the title compound (22 mg, 78%, 64% *ee*) as a colourless oil. **¹H-NMR** (500 MHz, $CDCl_3$) δ_H 7.34-7.27 (3H, m), 7.24-7.22 (2H, m), 4.93 (1H, dd, *J* = 13.0, 5.0), 4.88 (1H, dd, *J* = 13.0, 9.0), 4.25 (1H, td, *J* = 9.0, 5.0), 3.87 (1H, d, *J* = 9.0), 3.77 (3H, s), 3.57 (3H, s). **¹³C-NMR** (125 MHz, MeOD) δ_C 168.0 (CO), 167.4 (CO), 136.3 (ArC), 129.2 (ArCH), 128.6 (ArCH), 128.0 (ArCH), 77.5 (CH_2), 54.9 (CH), 53.1 (CH_3), 53.0 (CH_3), 43.1 (CH). **MS** (ES^+, MeOH) m/z = 282 ($[M+H]^+$, 30%), 299 ($[M+NH_4]^+$, 70%), 304 ($[M+Na]^+$, 100%). $[\boldsymbol{\alpha}]_{\boldsymbol{D}}^{\mathbf{20}}$ = −3.4 (*c* 1.0, $CHCl_3$).

The enantiomeric ratio (82:18) was determined by analytical HPLC with a Chiralpak AD-H (5 μm particle size; 250 x 4.6 mm; Daicel) column (hexane:2-propanol; 80:20; 1 mL min^{-1}, 210 nm): t_r (major) = 9.8 min, t_r (minor) = 14.2 min. The major enantiomer was assigned as (*R*) by comparison with literature data: $[\alpha]_D^{20}$ = −7.8 (*c* 0.8, $CHCl_3$), (*R*)-isomer (97% ee).[6]

Control experiment:

A flame-dried 5 mL microwave vial was charged with 1-(2-(piperidin-1-yl)ethyl)thiourea-Aib_4O^tBu (**9g**, 5.8 mg, 0.010 mmol), Z-GlyAib$_4$-L-AlaNH^{t}Bu (**9h**, 6.8 mg, 0.010 mmol) and *trans*-β-nitrostyrene (15 mg, 0.10 mmol). The vial was sealed, purged with N_2 and anhydrous CH_2Cl_2 (0.4 mL) added. Diethyl malonate (30 μL, 0.20 mmol) was added and the reaction stirred at room temperature for 72 h (66% conversion by ¹H-NMR). The solvents were removed *in vacuo* and the residue purified by column chromatography (hexane:EtOAc; 80:20) giving diethyl 2-(2-nitro-1-phenylethyl)malonate (21 mg, 68%, 4% *ee*) as a colourless oil.

The enantiomeric ratio (52:48) of the product was determined by analytical HPLC with a Chiralpak AD-H (5 μm particle size; 250 x 4.6 mm; Daicel) column (hexane:2-propanol; 80:20; 1 mL min^{-1}, 210 nm): t_r (major) = 8.7 min, t_r (minor) = 20.8 min.

Photoswitch experiment:

A flame-dried 5 mL microwave vial was charged with 1-(2-(piperidin-1-yl)ethyl)thiourea-GlyAib$_4$-L-AlaBni (**11**, 8.8 mg, 0.010 mmol) and *trans*-β-nitrostyrene (15 mg, 0.10 mmol). The vial was sealed, purged with N_2 and anhydrous CH_2Cl_2 (0.4 mL) added. Dimethyl malonate (23 μL, 0.20 mmol) was added and the reaction stirred at room temperature for 18 h (84% conversion by ^{1}H-NMR).

The enantiomeric ratio (37:63) of the crude product was determined by analytical HPLC with a Chiralpak AD-H (5 μm particle size; 250 x 4.6 mm; Daicel) column (hexane:2-propanol; 85:15; 1 mL min^{-1}, 210 nm): t_r (minor) = 11.3 min, t_r (major) = 17.3 min.

The catalyst was recovered by flash column chromatography (SiO_2, 1-40% MeOH in CH_2Cl_2) and dissolved in CD_2Cl_2 (1.5 mL). The solution was transferred into a Hellma 3500 μL quartz cuvette (1 cm path length), ^{i}PrNH$_2$ (8.5 μL, 0.10 mmol) added and the mixture irradiated at 365 nm for 1 h (>95% conversion by ^{1}H-NMR). The solvents were removed *in vacuo* and the residual red solid dissolved in anhydrous CH_2Cl_2 (0.4 mL). The solution was added to a flame-dried 5 mL microwave vial charged with *trans*-β-nitrostyrene (15 mg, 0.10 mmol). Dimethyl malonate (23 μL, 0.20 mmol) was added and the reaction stirred at room temperature for 72 h (>95% conversion by ^{1}H-NMR).

The enantiomeric ratio (77:23) of the crude product was determined by analytical HPLC with a Chiralpak AD-H (5 μm particle size; 250 x 4.6 mm; Daicel) column (hexane:2-propanol; 85:15; 1 mL min$^-$1, 210 nm): t_r (major) = 11.4 min, t_r (minor) = 17.4 min. The solvents were removed and the residue purified by column chromatography (Hexane:EtOAc; 80:20) giving dimethyl 2-(2-nitro-1-phenylethyl)malonate as a yellow oil (22 mg, 0.078 mmol, 78%).

^{1}H-NMR of 5-Bromo-7-nitroindoline (Bni) 1

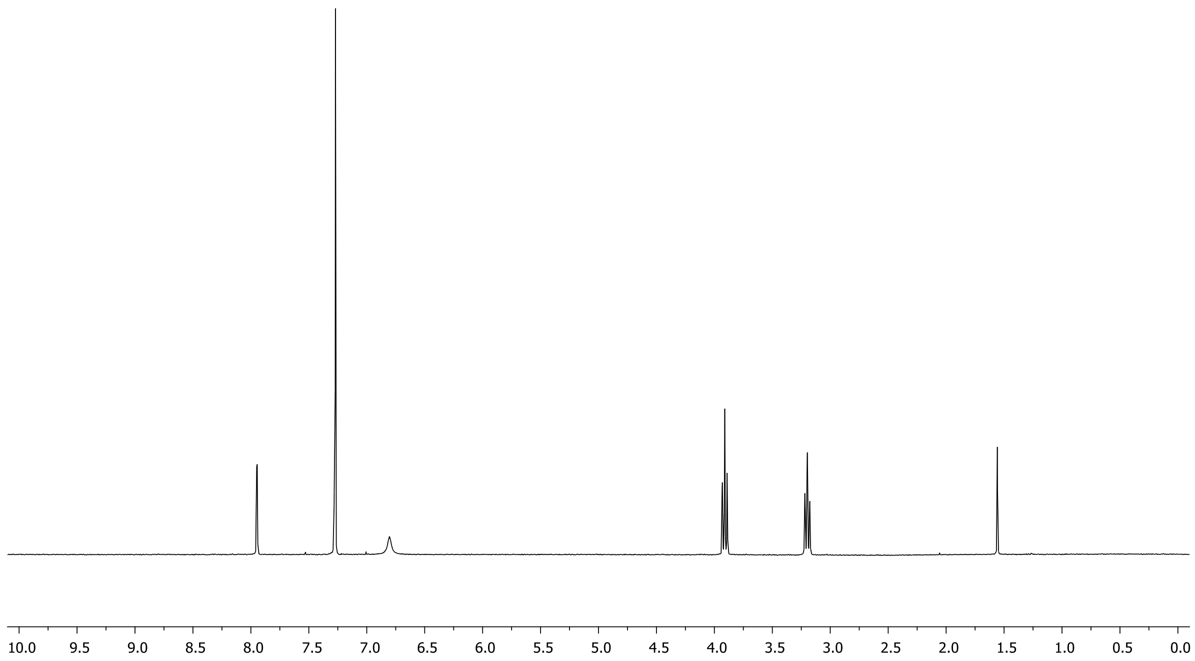

^{13}C-NMR of 5-Bromo-7-nitroindoline (Bni) 1

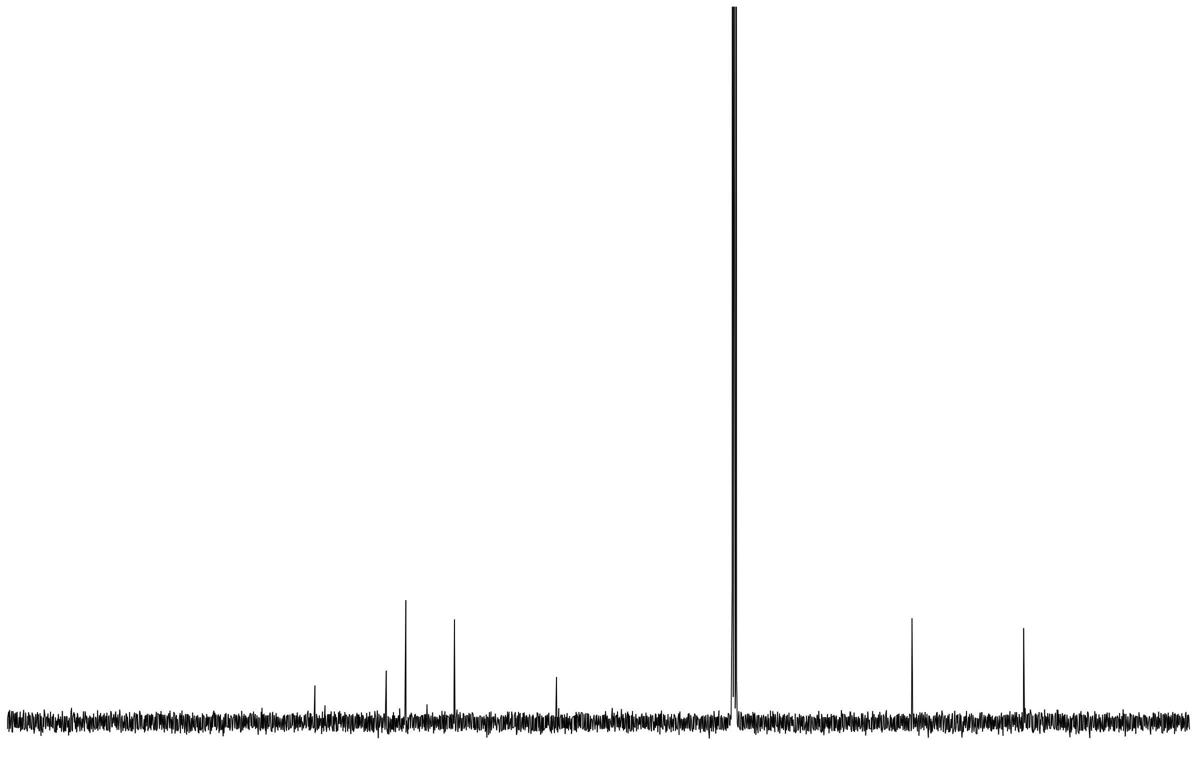

^{1}H-NMR of Fmoc-L-AlaBni

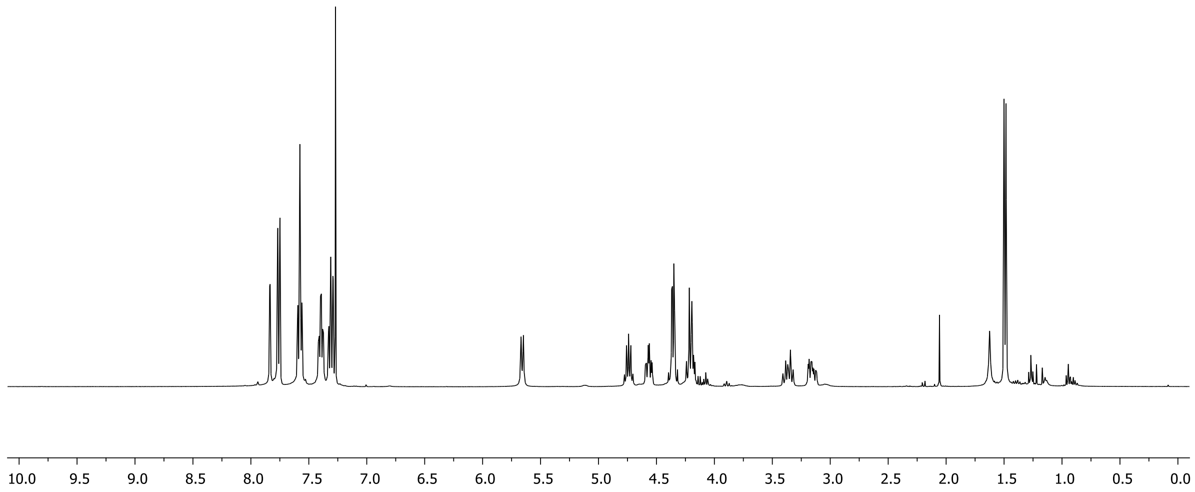

^{13}C-NMR of Fmoc-L-AlaBni

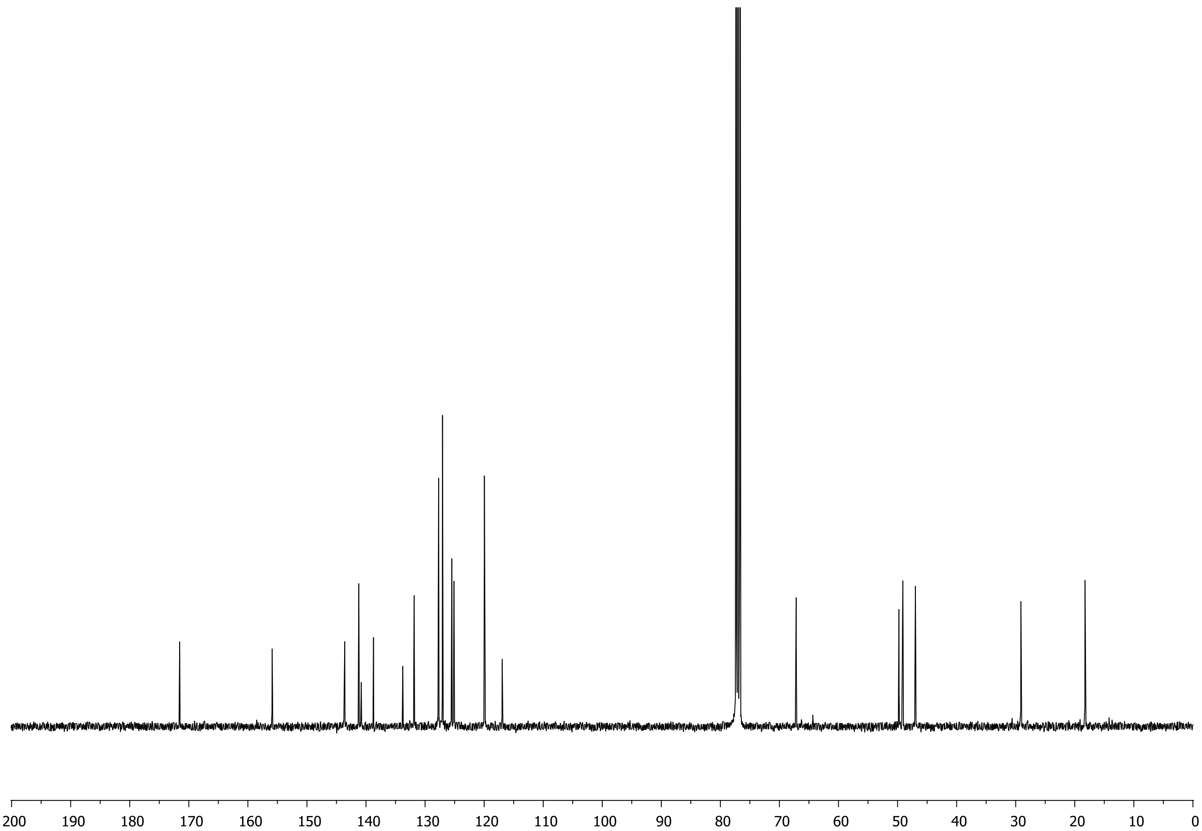

^{1}H-NMR of H-L-AlaBni 2

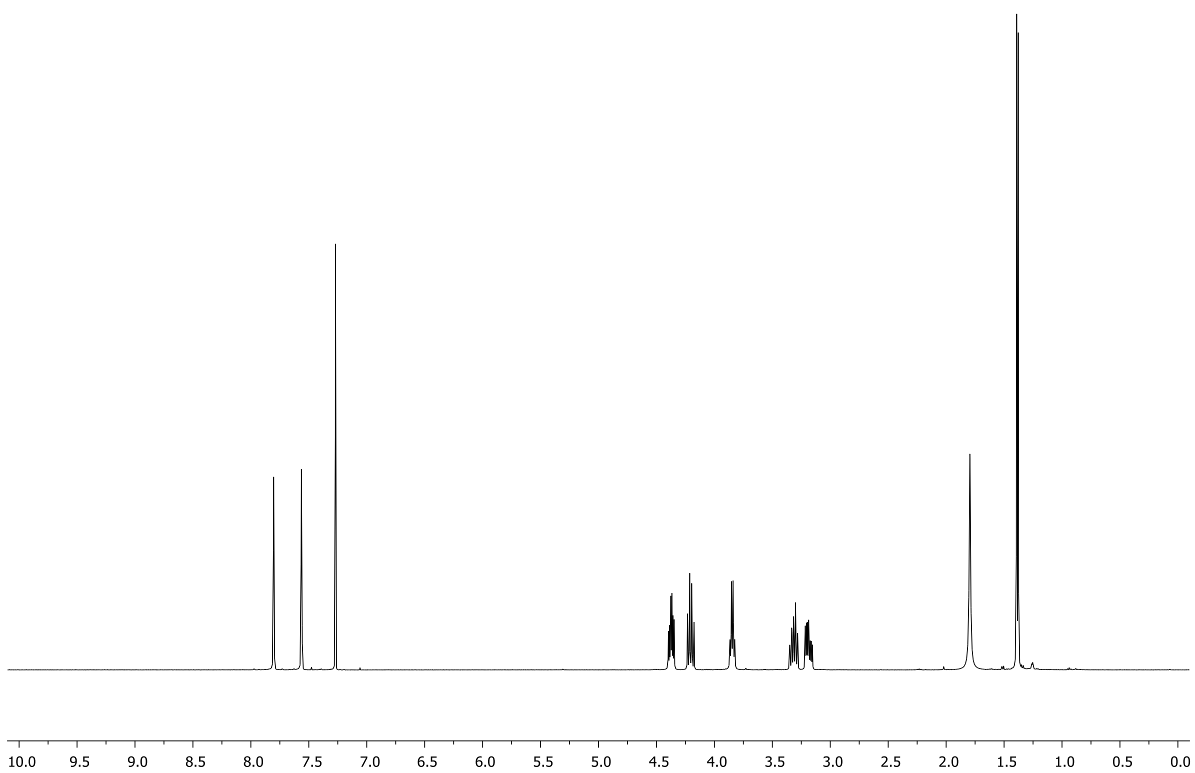

^{13}C-NMR of H-L-AlaBni 2

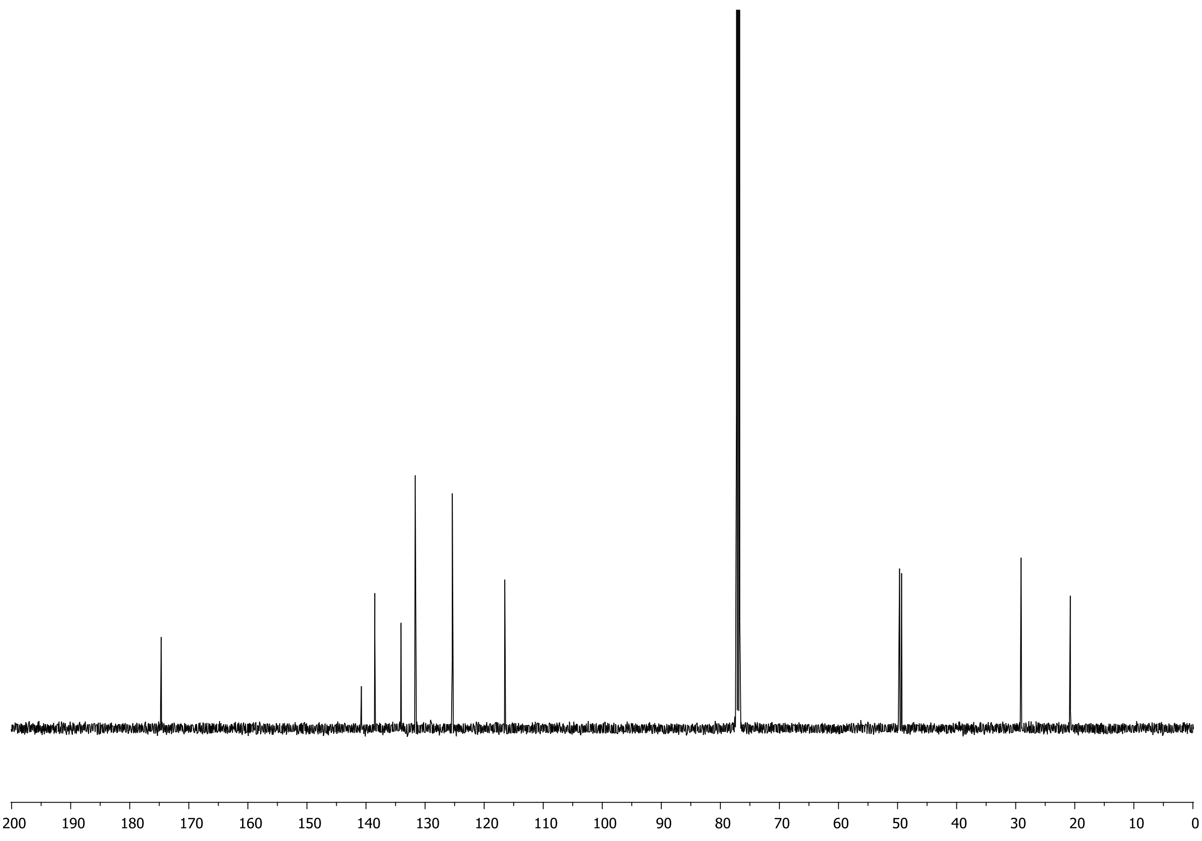

^{1}H-NMR of Z-L-Phe-L-AlaBni

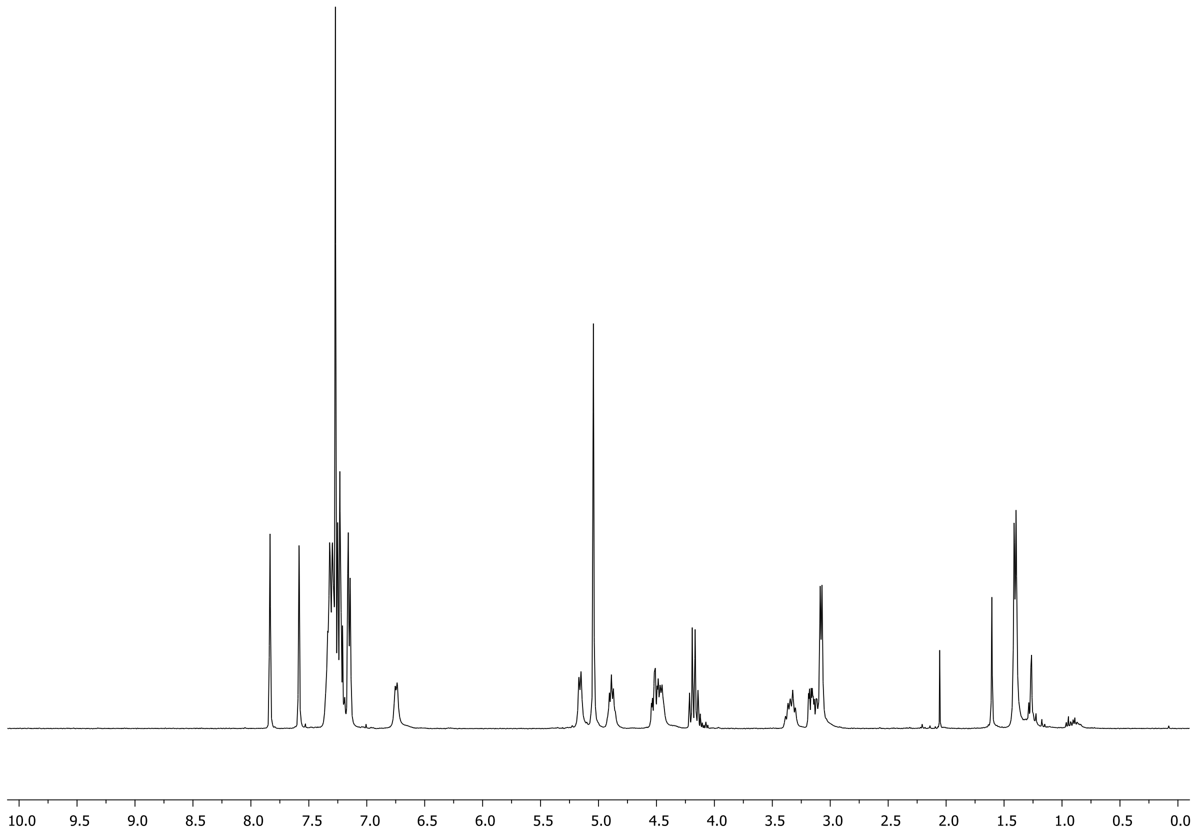

^{13}C-NMR of Z-L-Phe-L-AlaBni

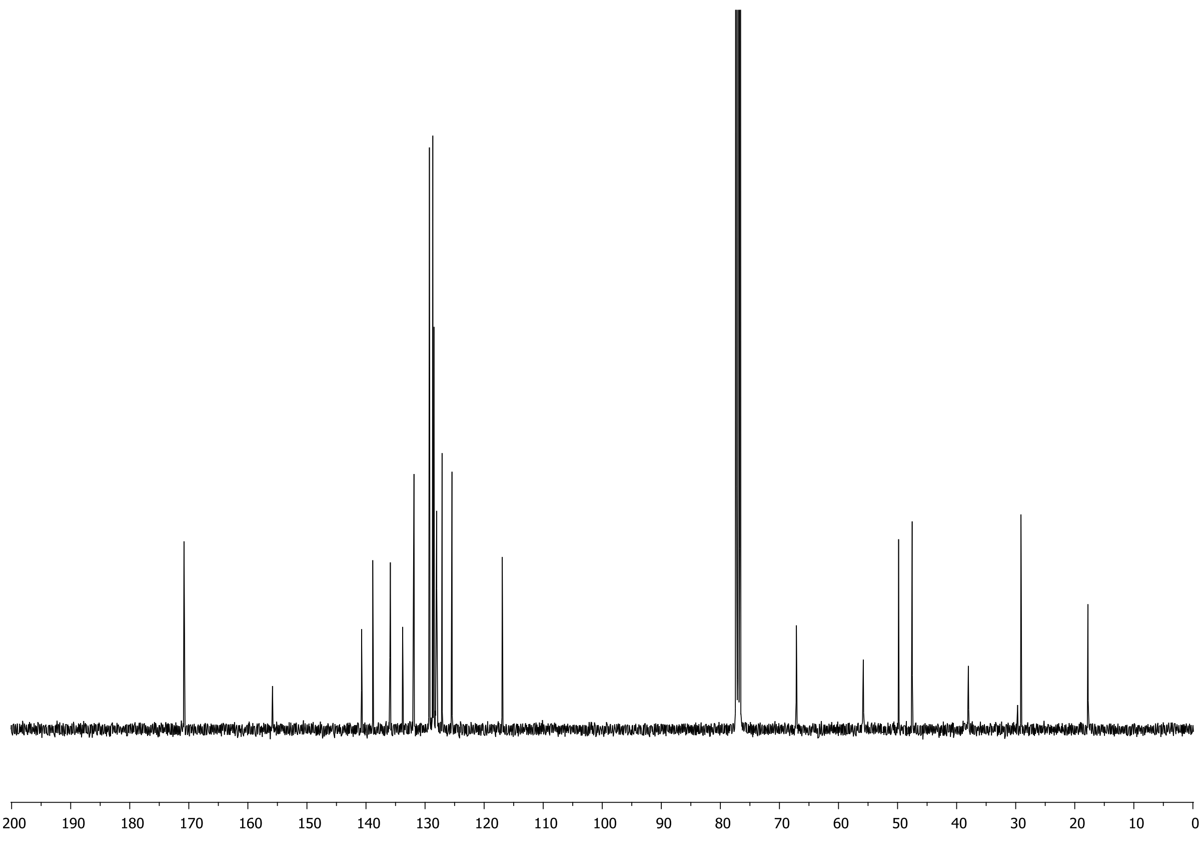

^{1}H-NMR of Z-(*R*)-Aib*-Aib$_4$-L-AlaBni 4

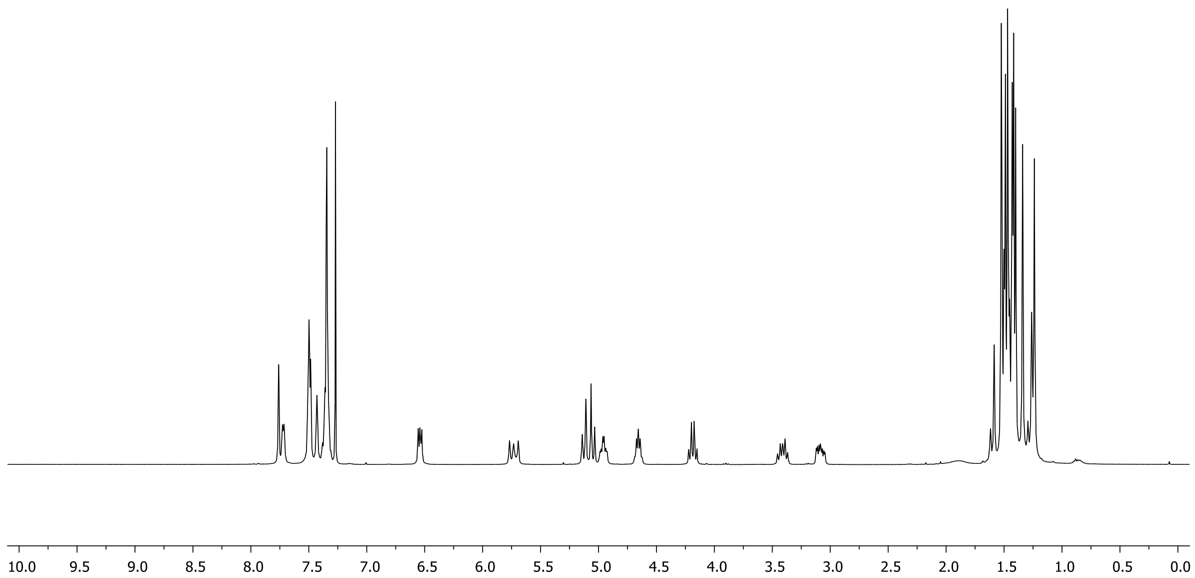

^{13}C-NMR of Z-(*R*)-Aib*-Aib$_4$-L-AlaBni 4

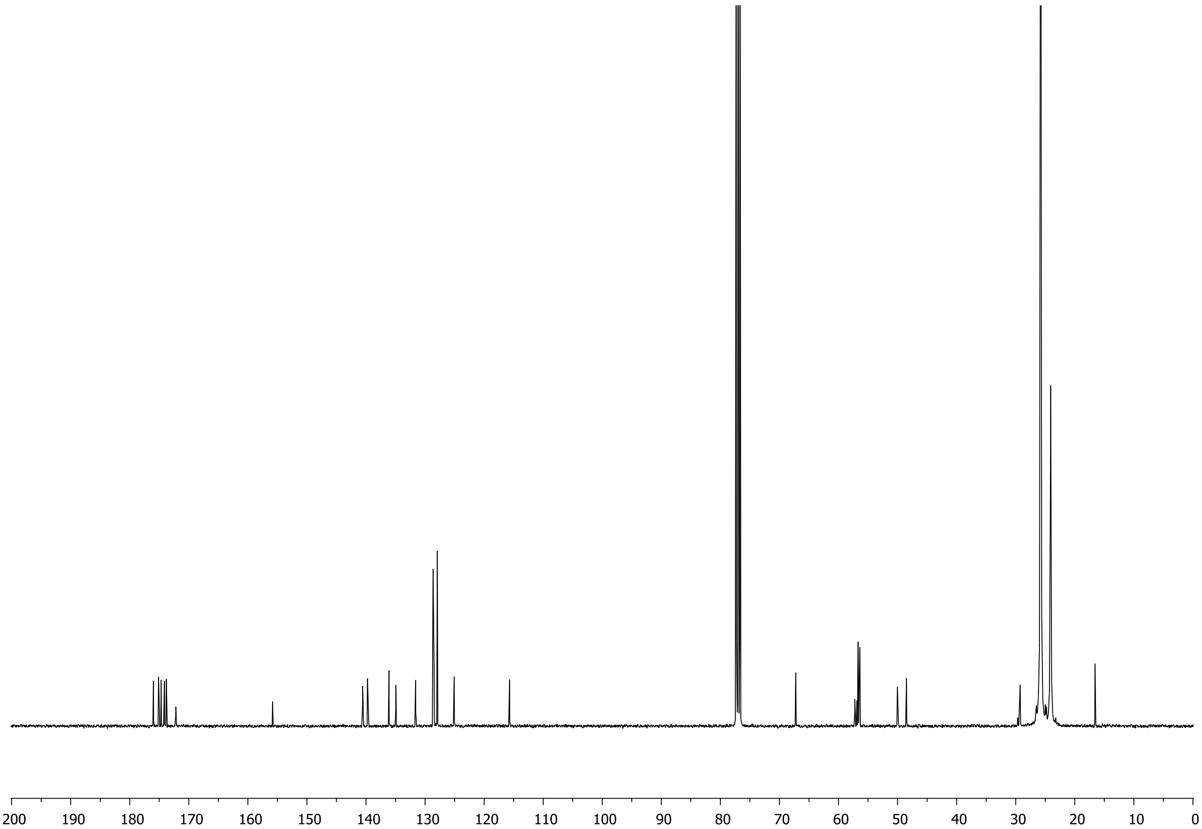

^{1}H-NMR of Z-Aib*-Aib$_4$-L-AlaNH^{i}Pr 5

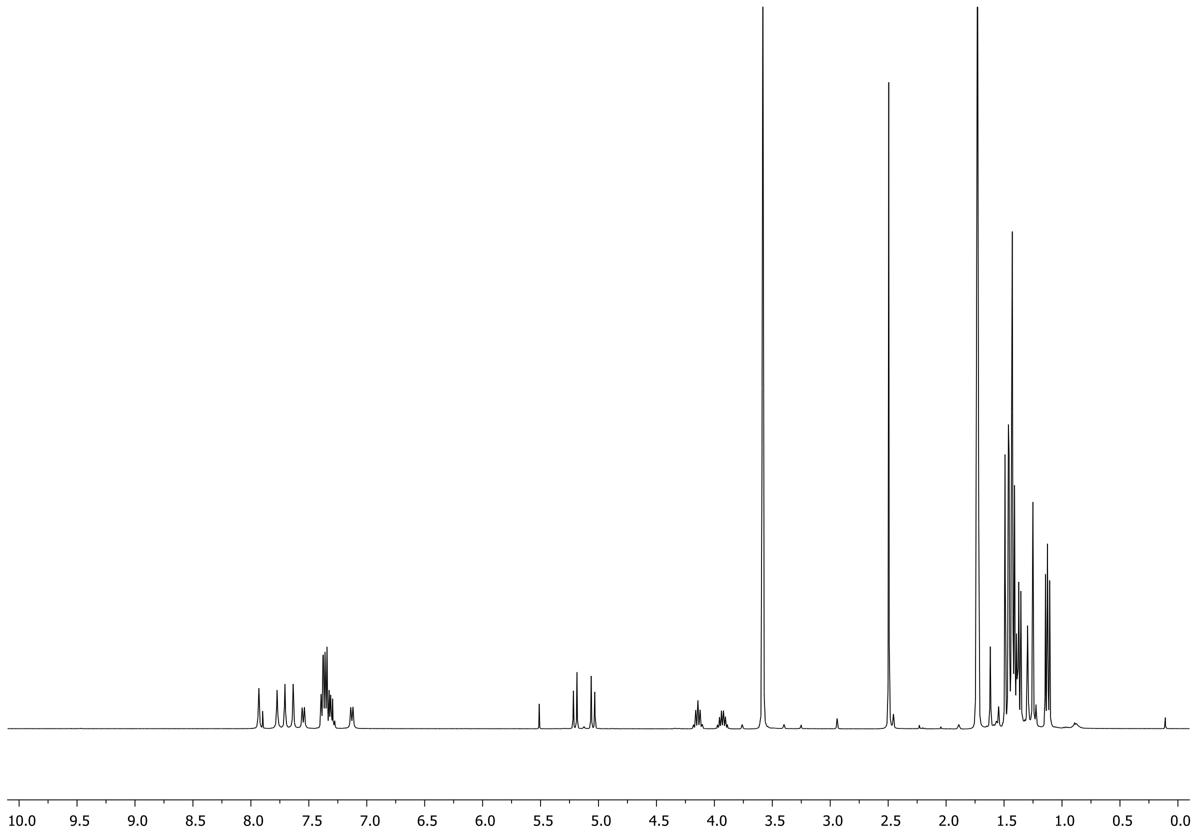

^{13}C-NMR of Z-Aib*-Aib$_4$-L-AlaNH^{i}Pr 5

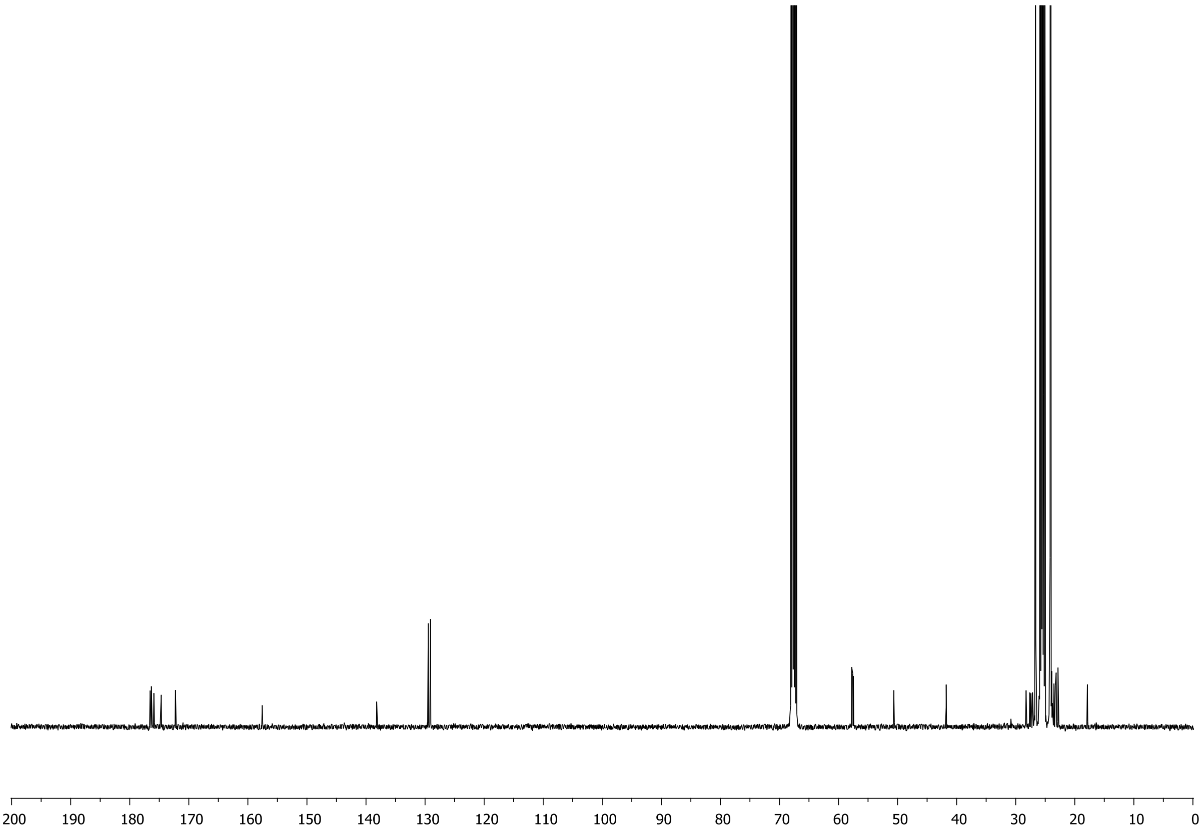

^{1}H-NMR of Z-L-Ala-NH-$(CH_2)_2$-N($(CH_2)_2$NHBoc$)_2$

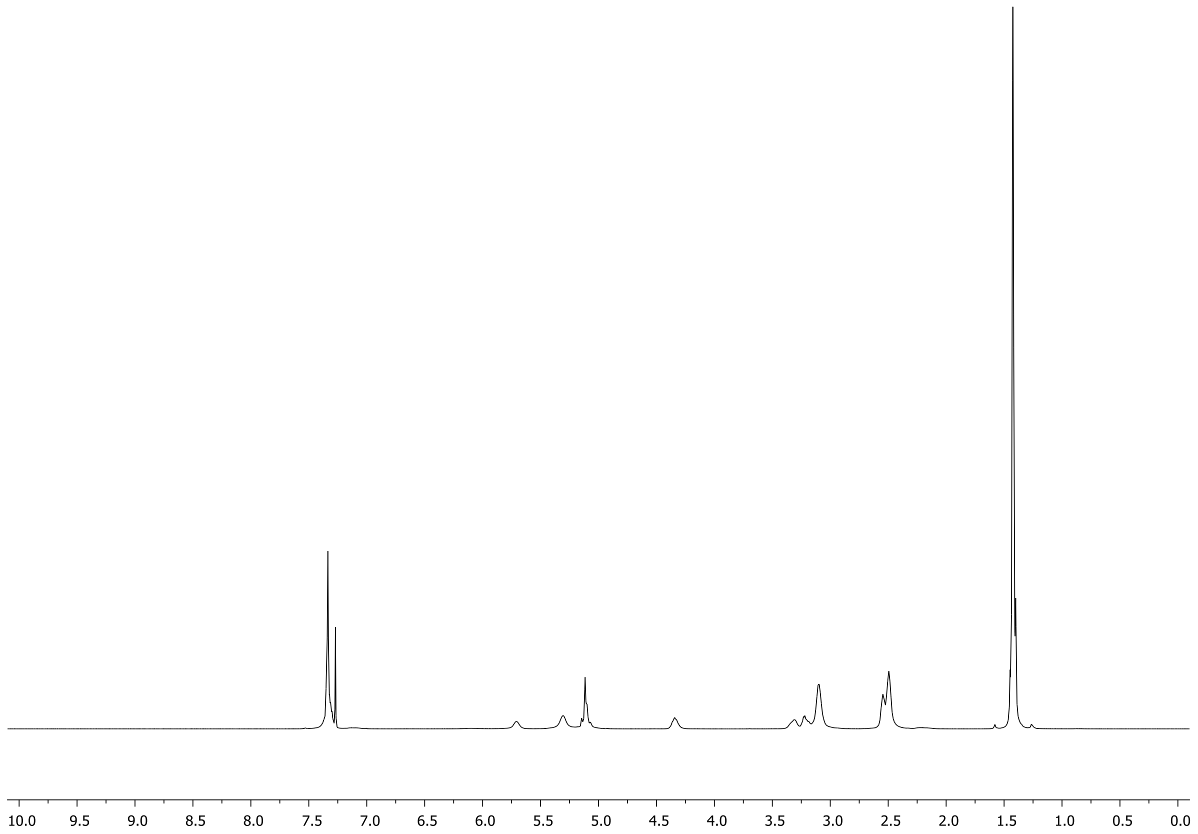

^{13}C-NMR of Z-L-Ala-NH-$(CH_2)_2$-N($(CH_2)_2$NHBoc$)_2$

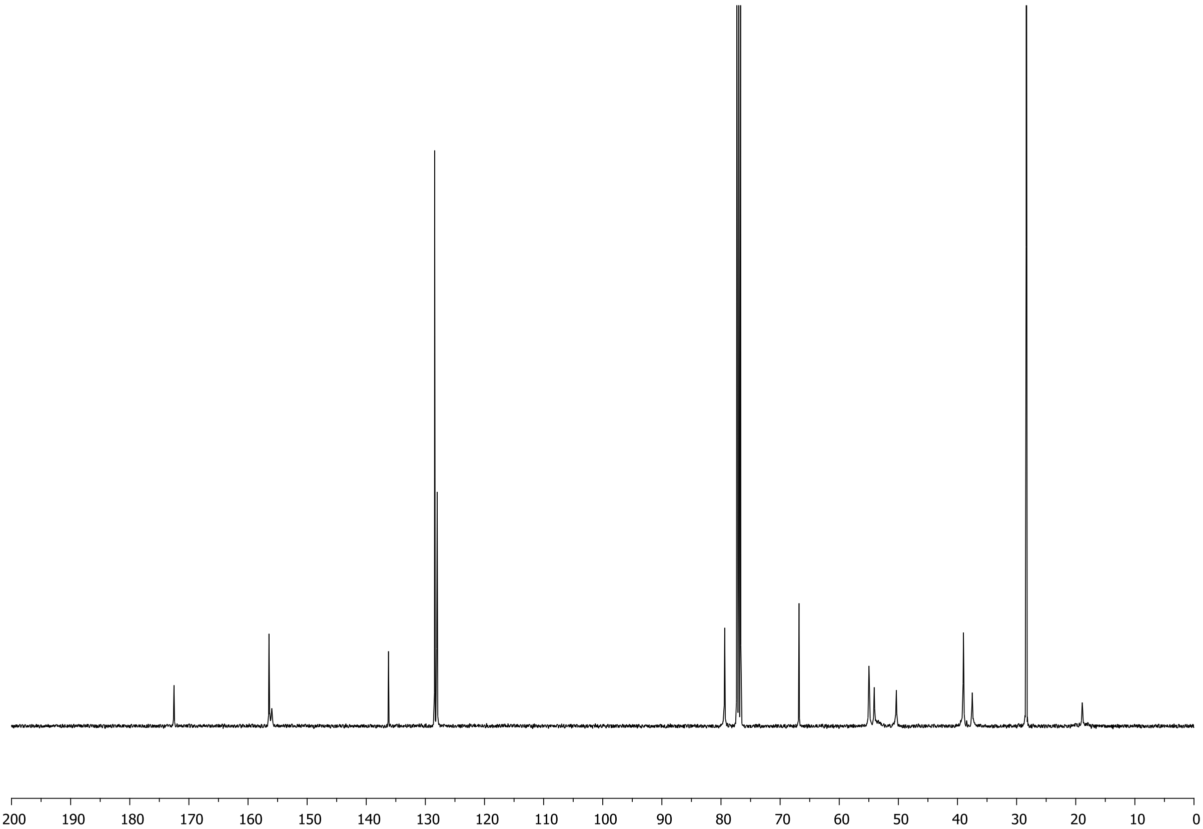

^{1}H-NMR of Z-(*R*)-Aib*-Aib$_4$-L-AlaNH-(CH$_2$)$_2$-N((CH$_2$)$_2$NHBoc)$_2$

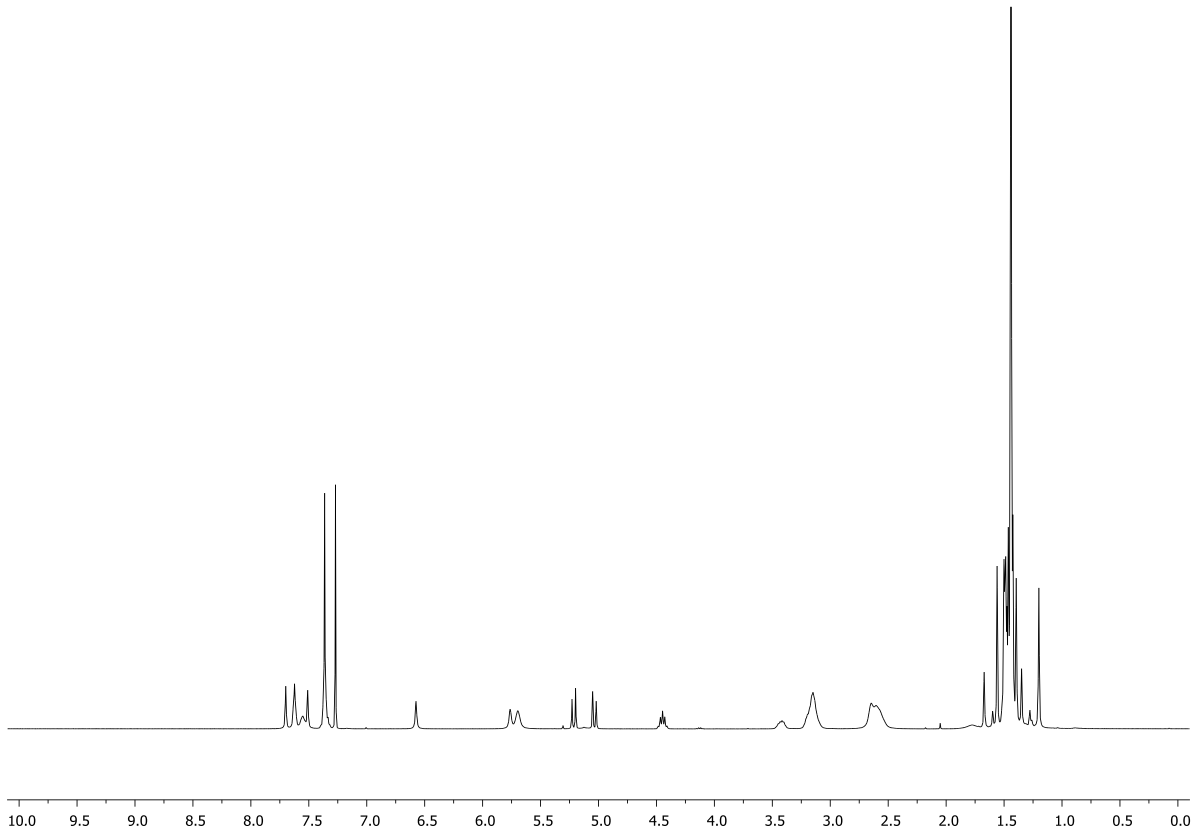

^{13}C-NMR of Z-(*R*)-Aib*-Aib$_4$-L-AlaNH-(CH$_2$)$_2$-N((CH$_2$)$_2$NHBoc)$_2$

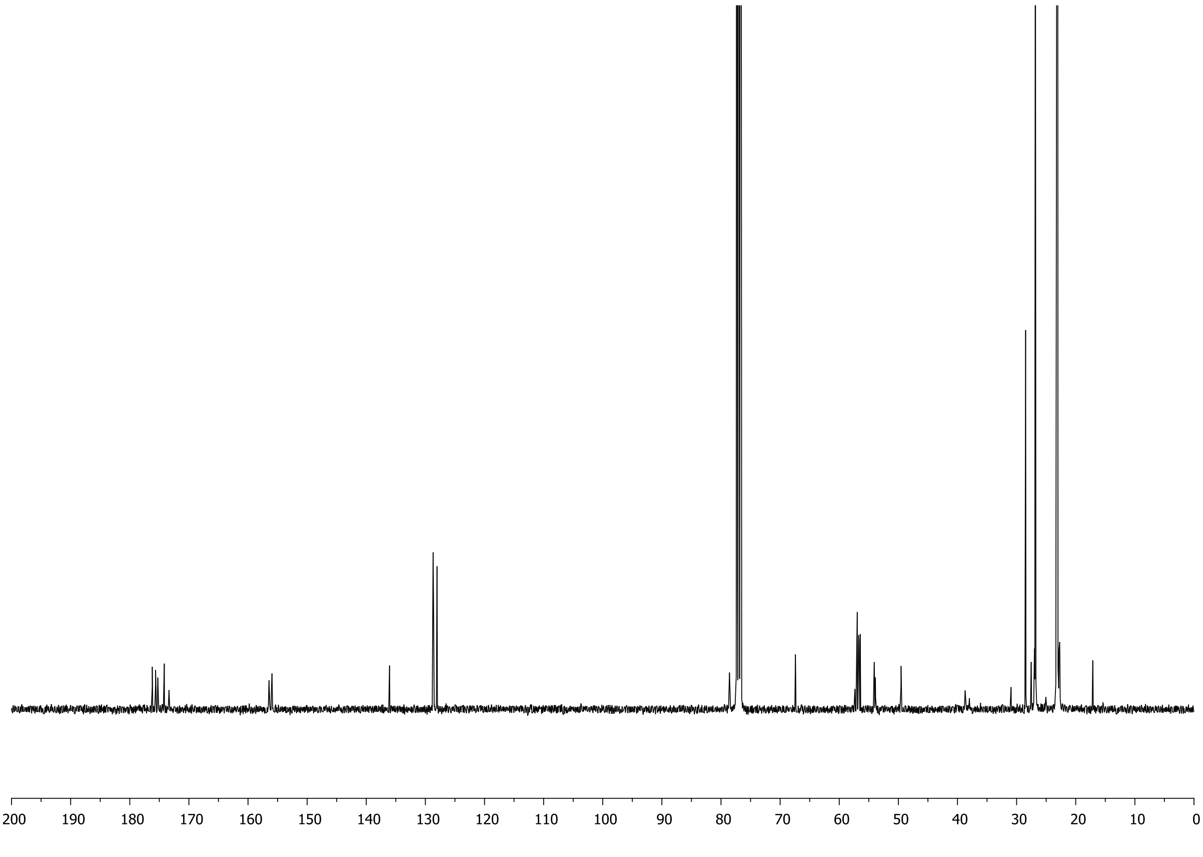

^{1}H-NMR of Z-(*R*)-Aib*-Aib$_4$-L-AlaNH-$(CH_2)_2$-N($(CH_2)_2NH_2$)$_2$ 8

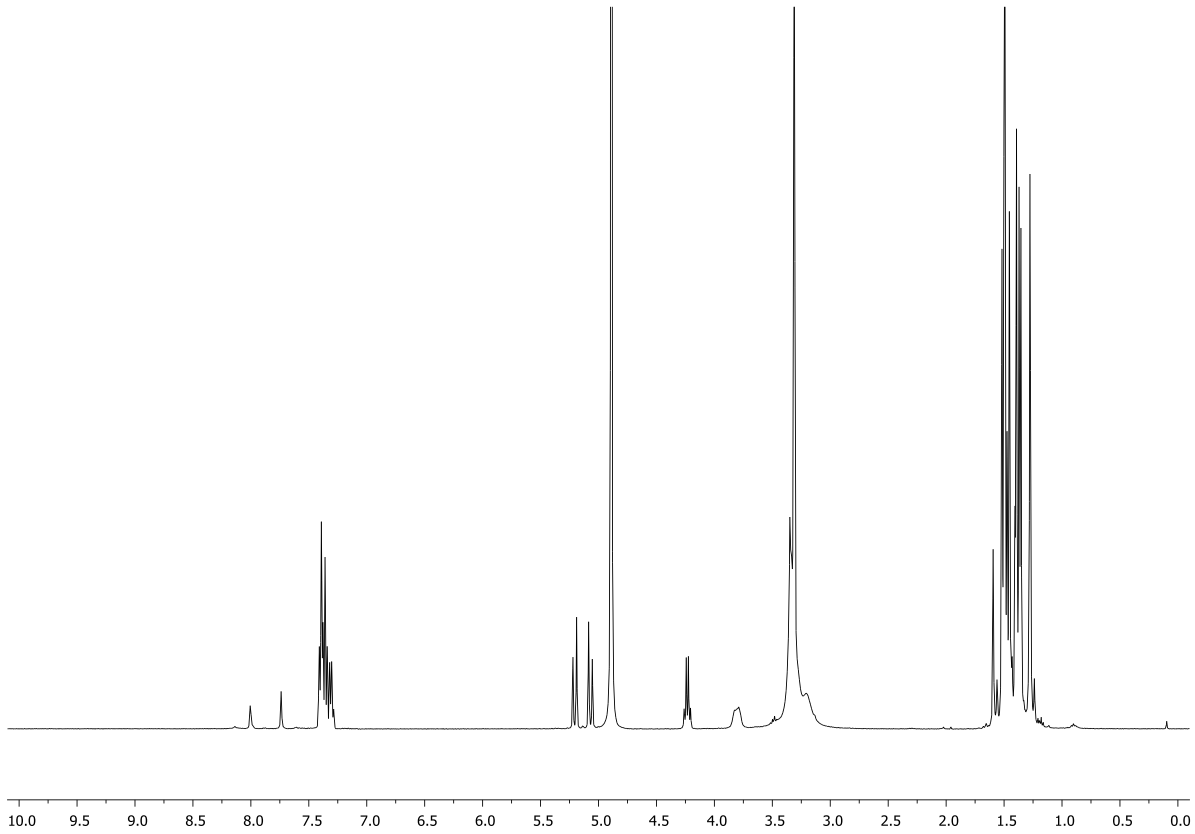

^{13}C-NMR of Z-(*R*)-Aib*-Aib$_4$-L-AlaNH-$(CH_2)_2$-N($(CH_2)_2NH_2$)$_2$ 8

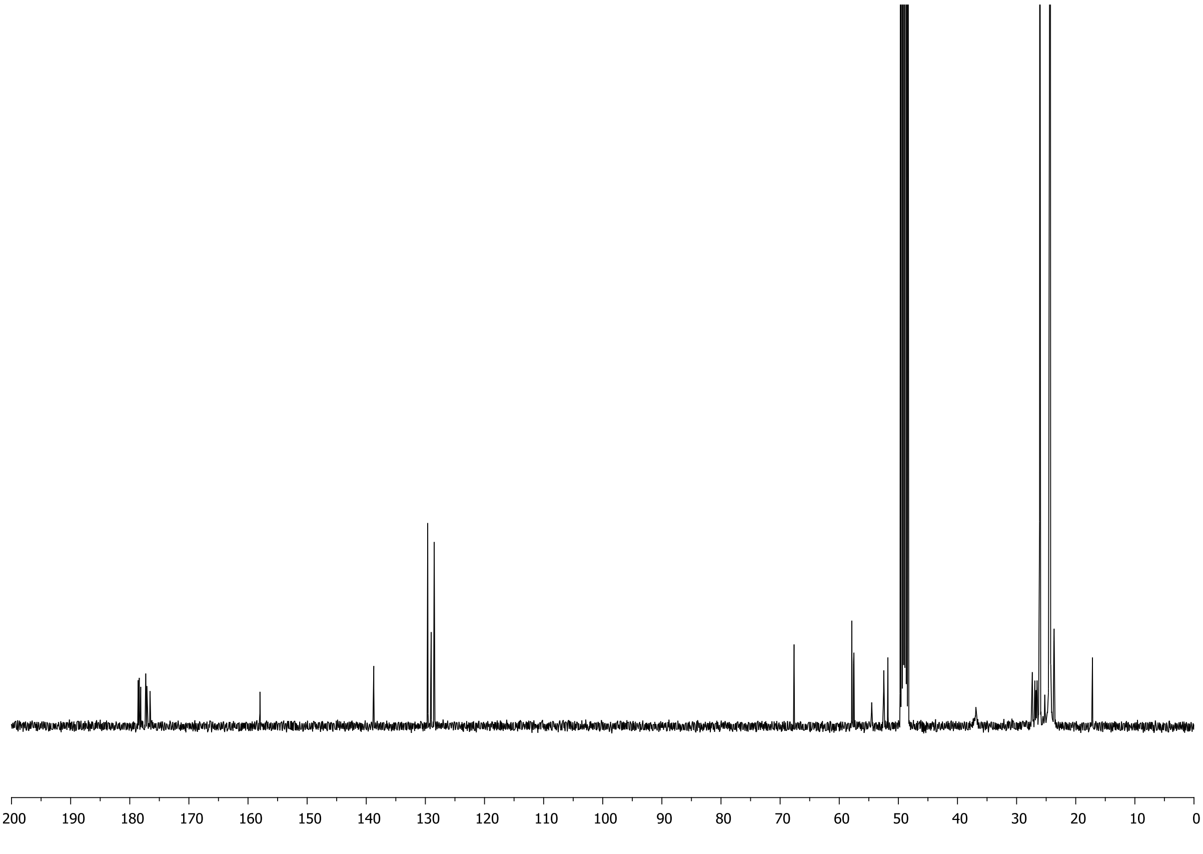

^{1}H-NMR of N_3Aib_4-L-AlaNH^{t}Bu

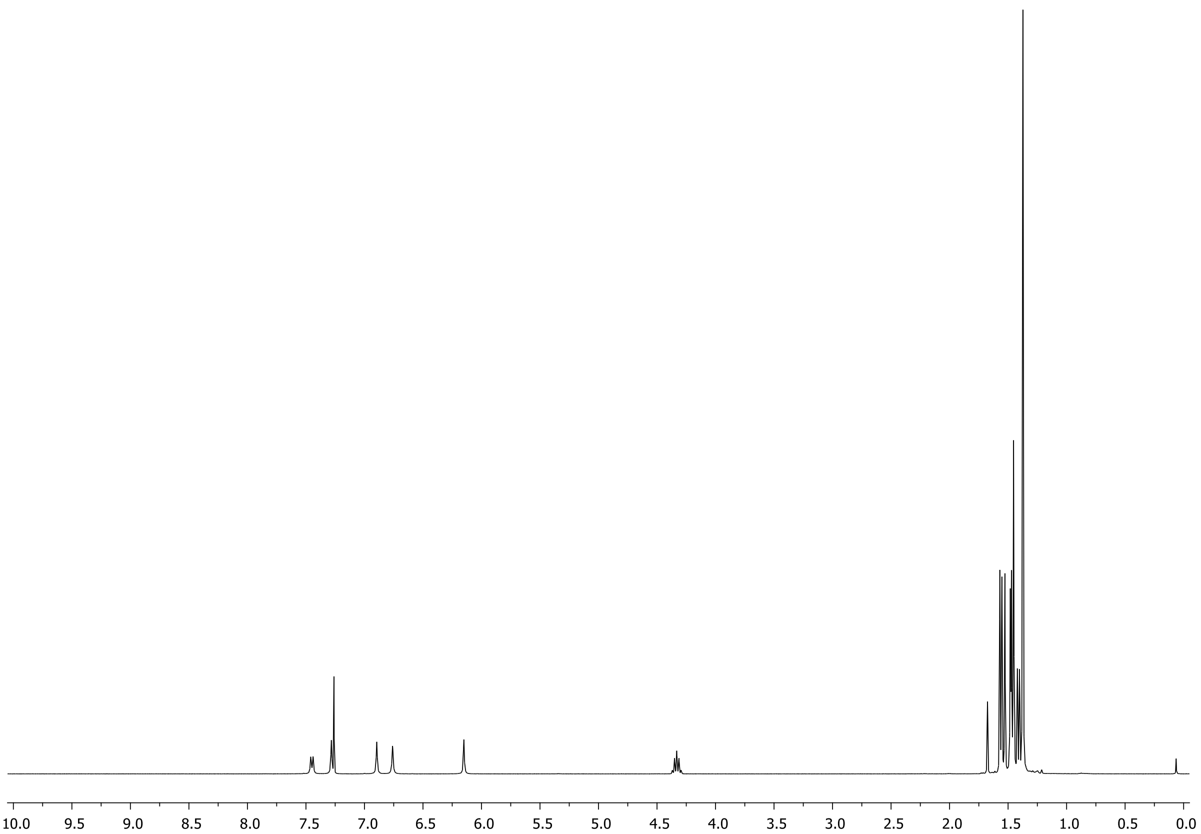

^{13}C-NMR of N_3Aib_4-L-AlaNH^{t}Bu

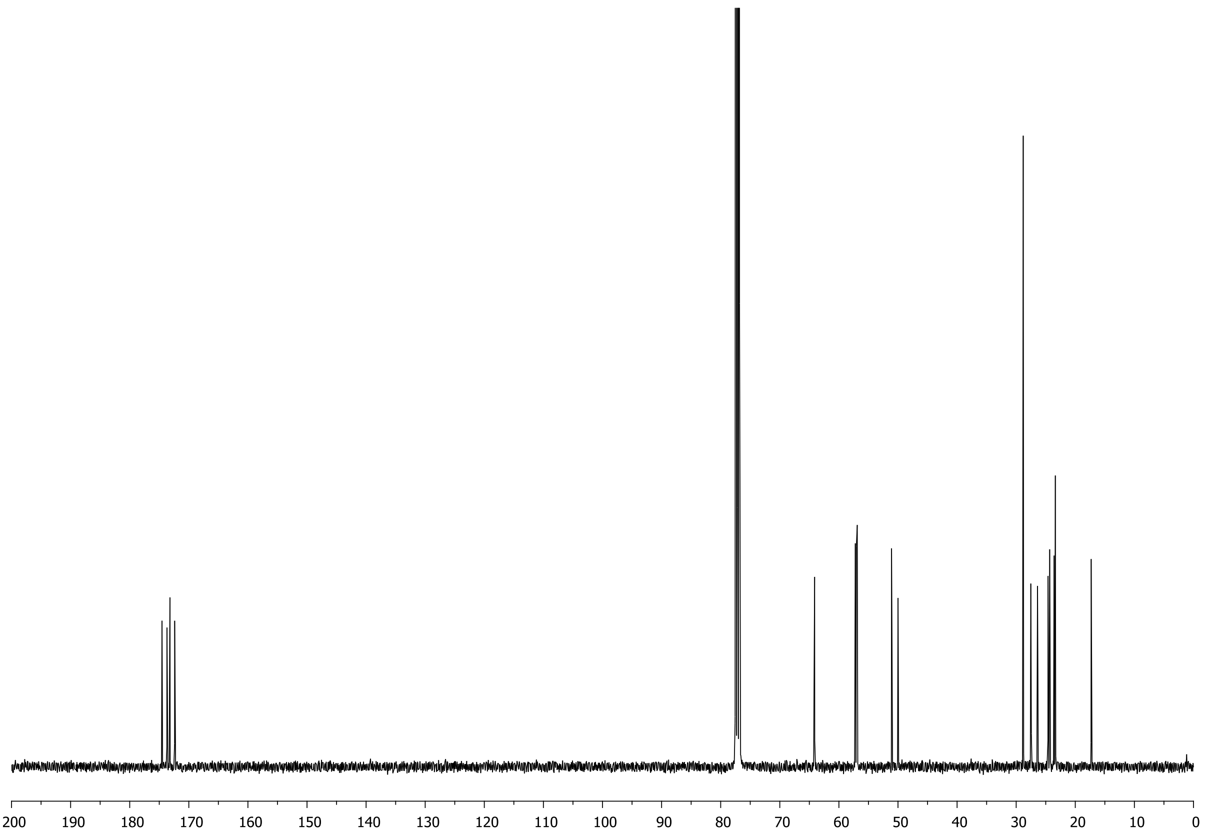

^{1}H-NMR of Z-GlyAib$_4$-L-AlaNH^{t}Bu 9h

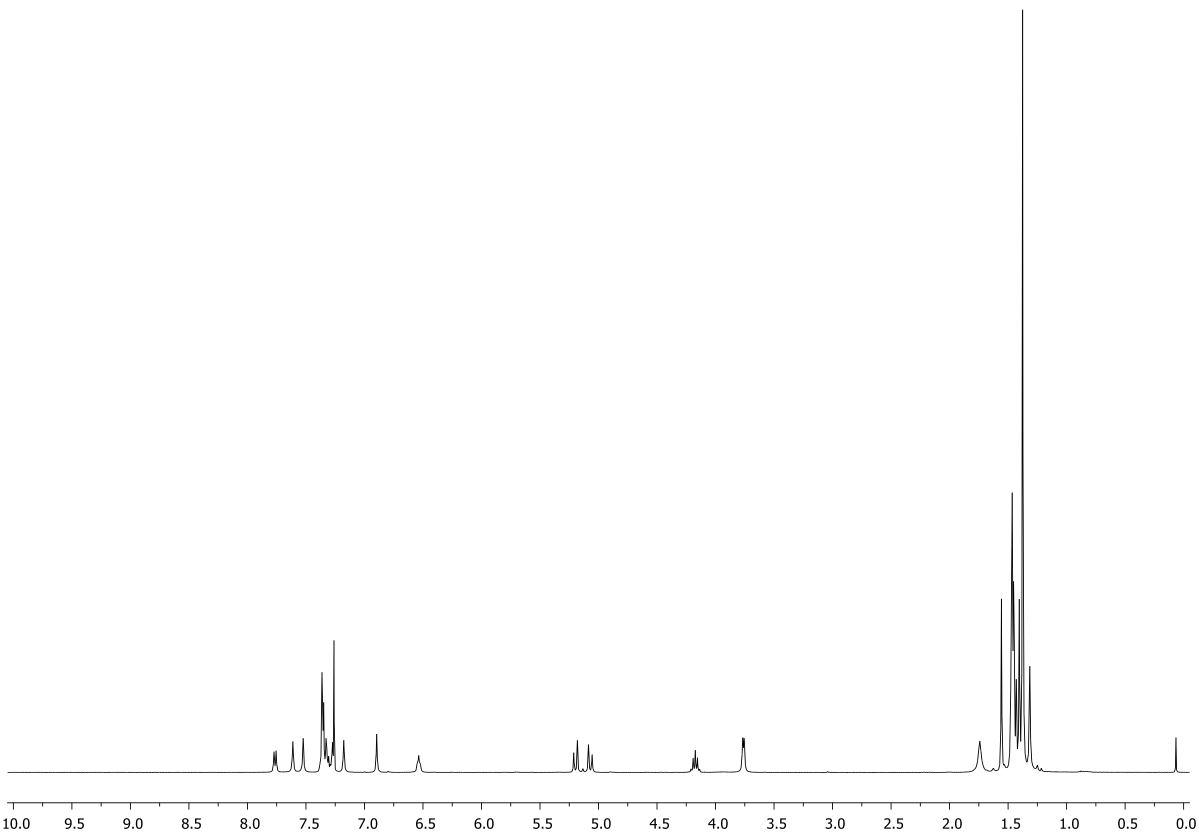

^{13}C-NMR of Z-GlyAib$_4$-L-AlaNH^{t}Bu 9h

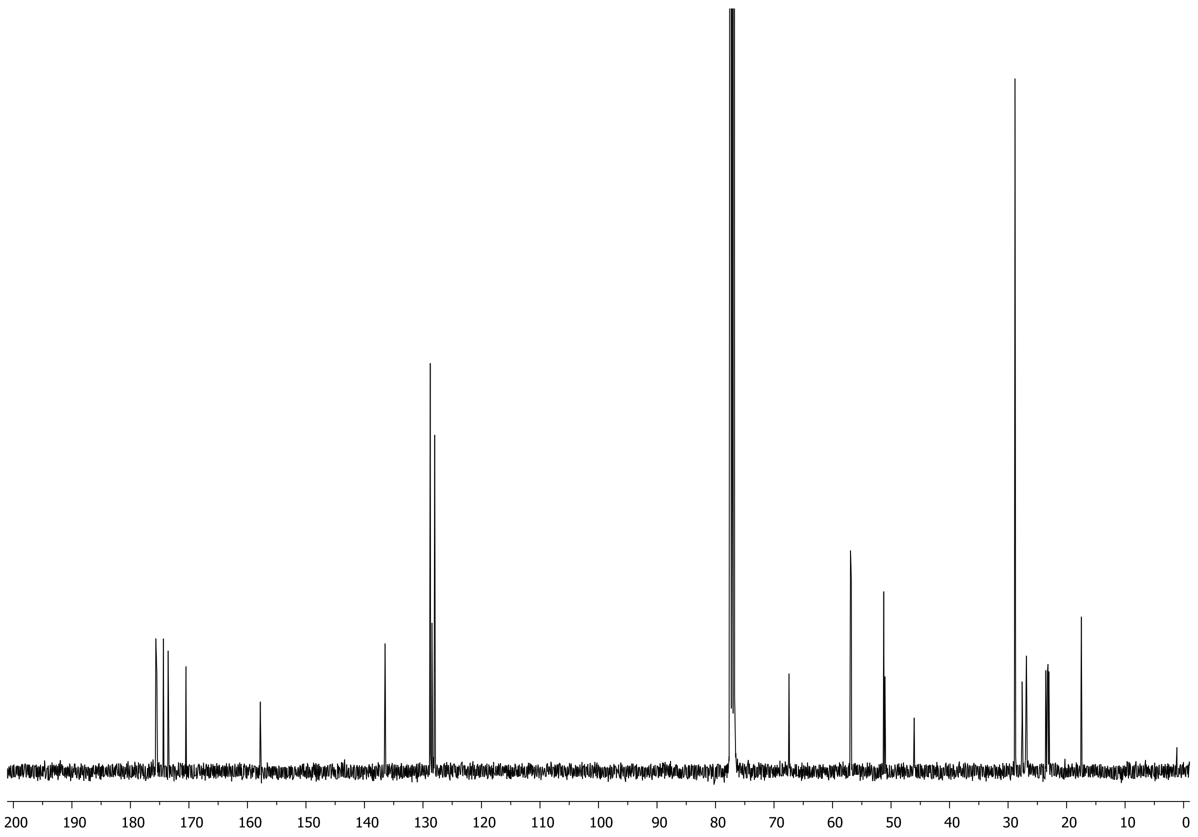

^{1}H-NMR of 1-(2-methyl-1-(piperidin-1-yl)propan-2-yl)thiourea-Aib$_4$-L-AlaNH^{t}Bu 9a

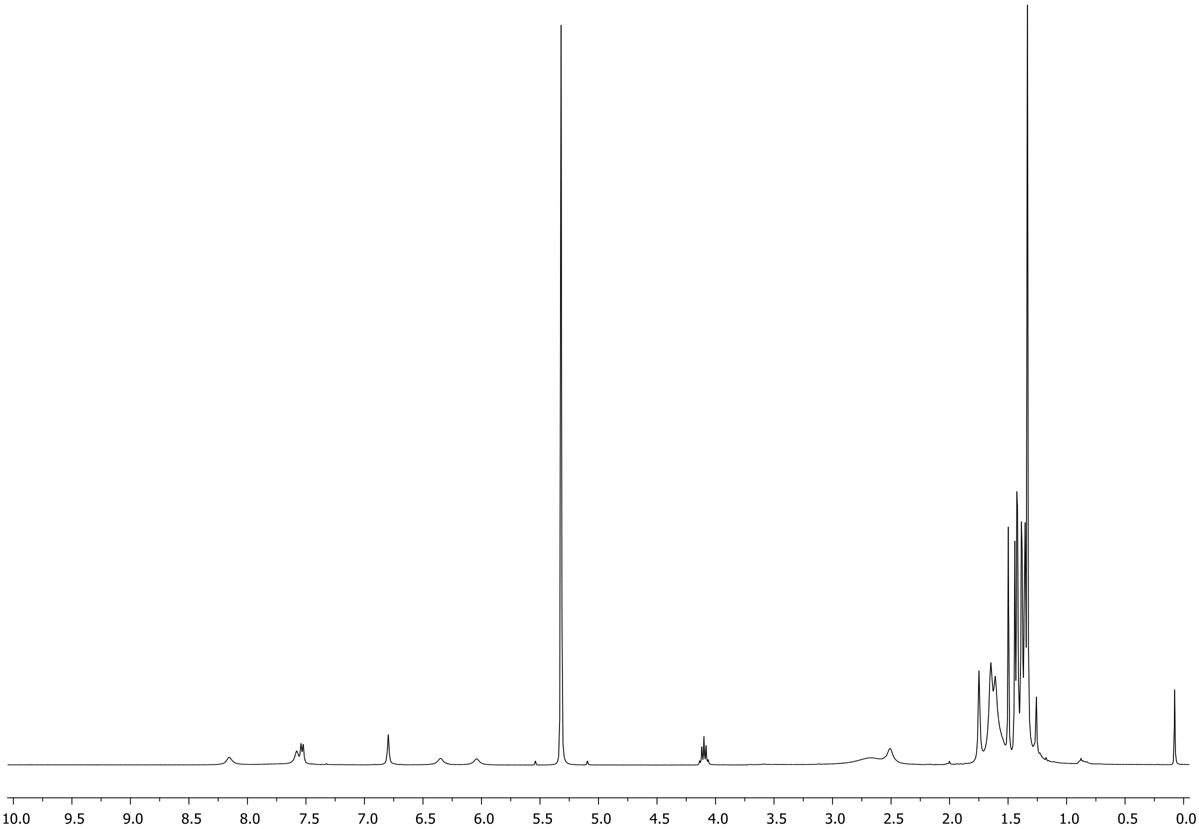

^{13}C-NMR of 1-(2-methyl-1-(piperidin-1-yl)propan-2-yl)thiourea-Aib$_4$-L-AlaNH^{t}Bu 9a

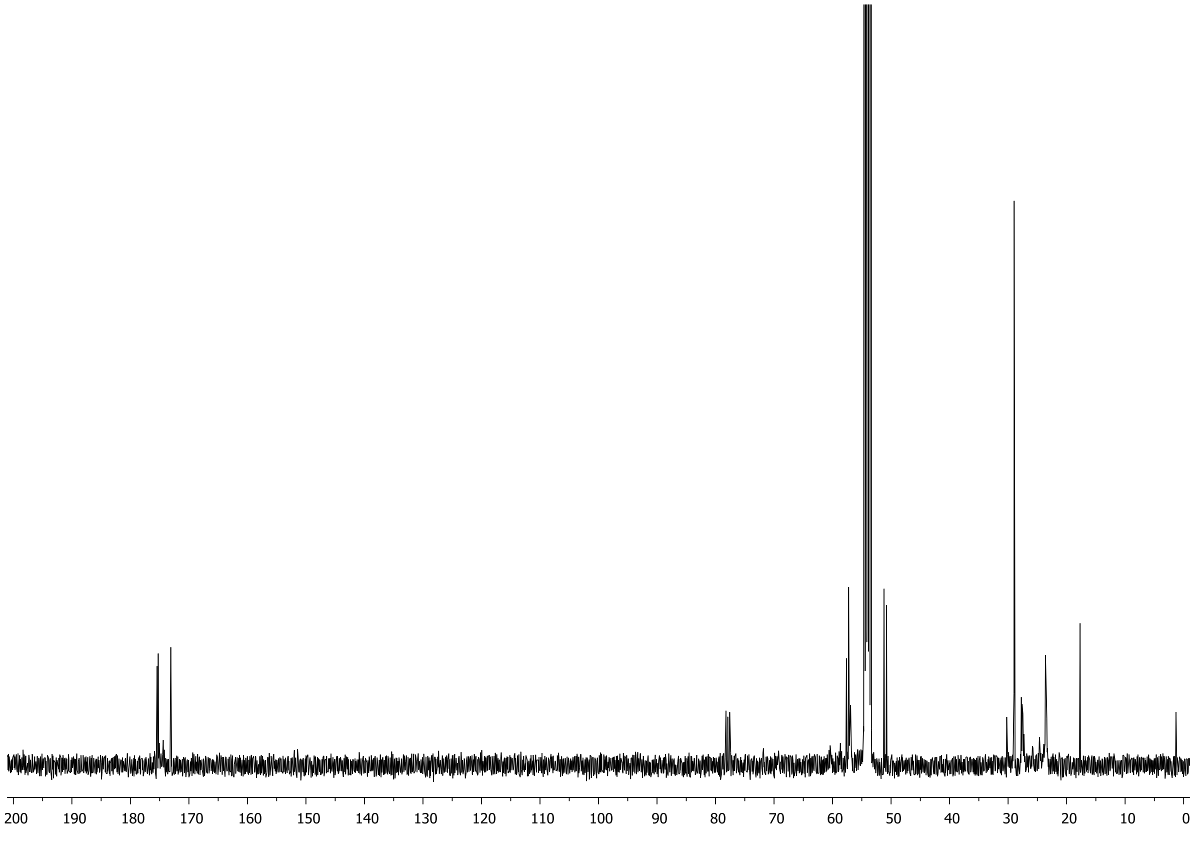

^{1}H-NMR of 1-(2-methyl-1-(piperidin-1-yl)propan-2-yl)thiourea-GlyAib$_4$-L-AlaNH^{t}Bu 9b

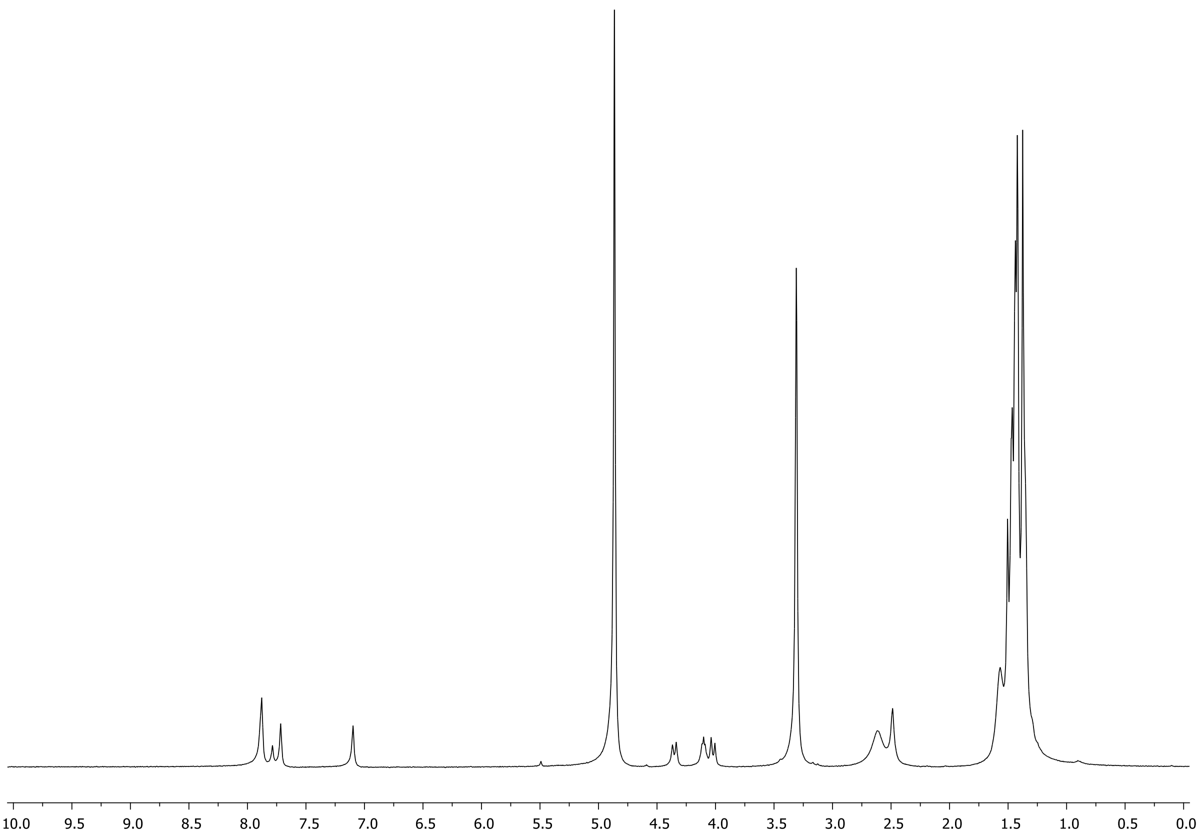

^{13}C-NMR of 1-(2-methyl-1-(piperidin-1-yl)propan-2-yl)thiourea-GlyAib$_4$-L-AlaNH^{t}Bu 9b

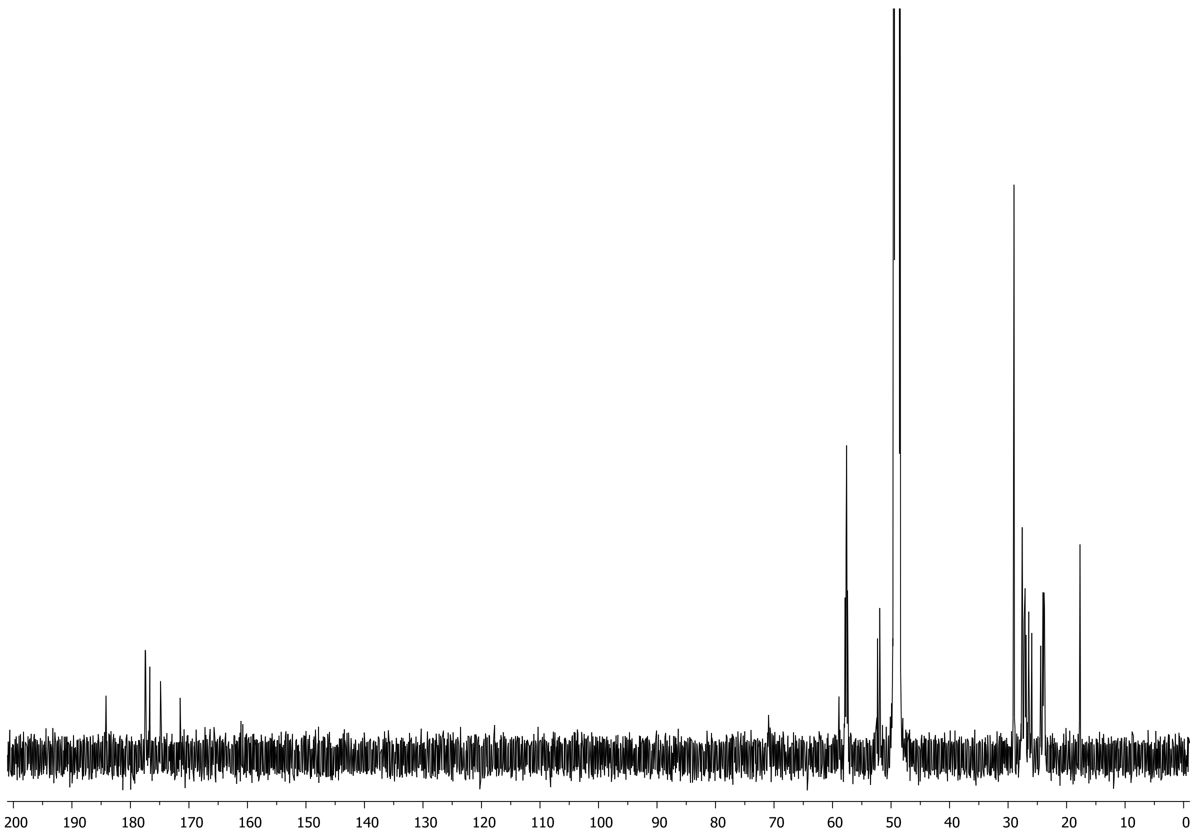

^{1}H-NMR of 1-(2-methyl-2-(piperidin-1-yl)propyl)thiourea-Aib$_4$-L-AlaNH^{t}Bu 9c

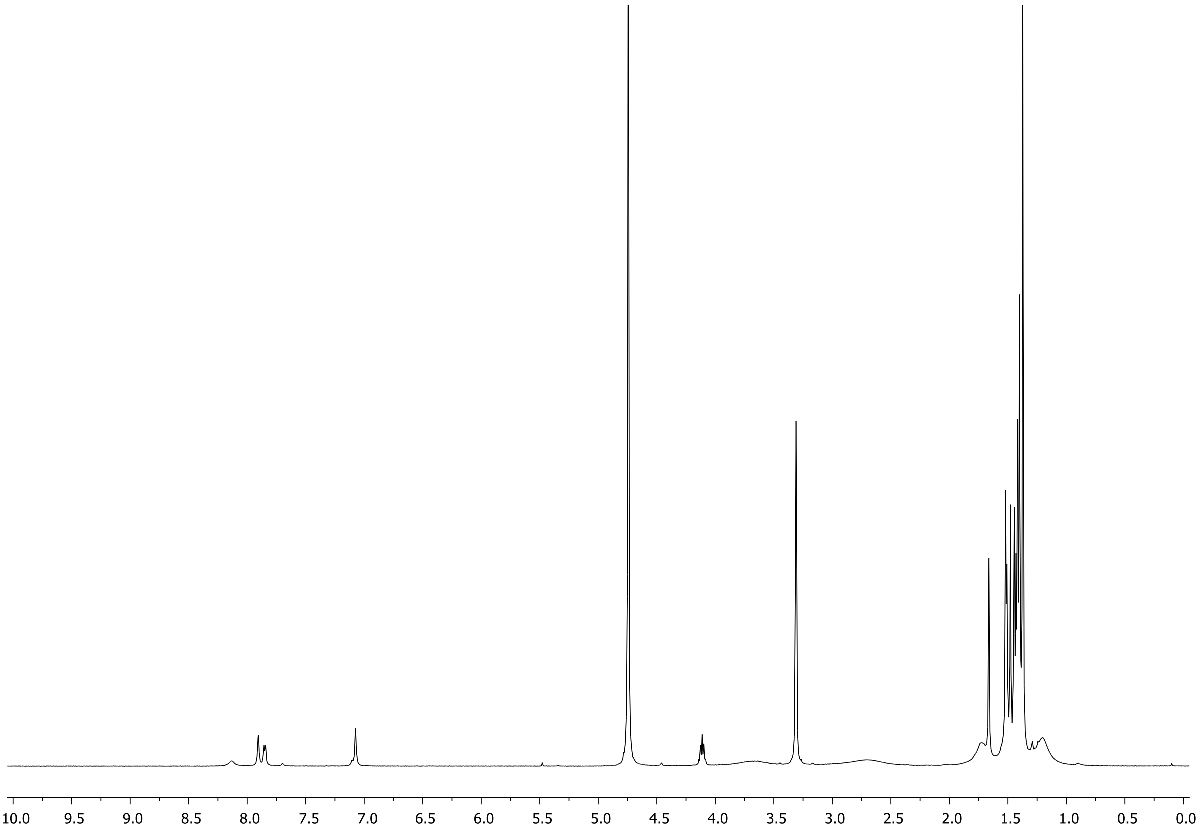

^{13}C-NMR of 1-(2-methyl-2-(piperidin-1-yl)propyl)thiourea-Aib$_4$-L-AlaNH^{t}Bu 9c

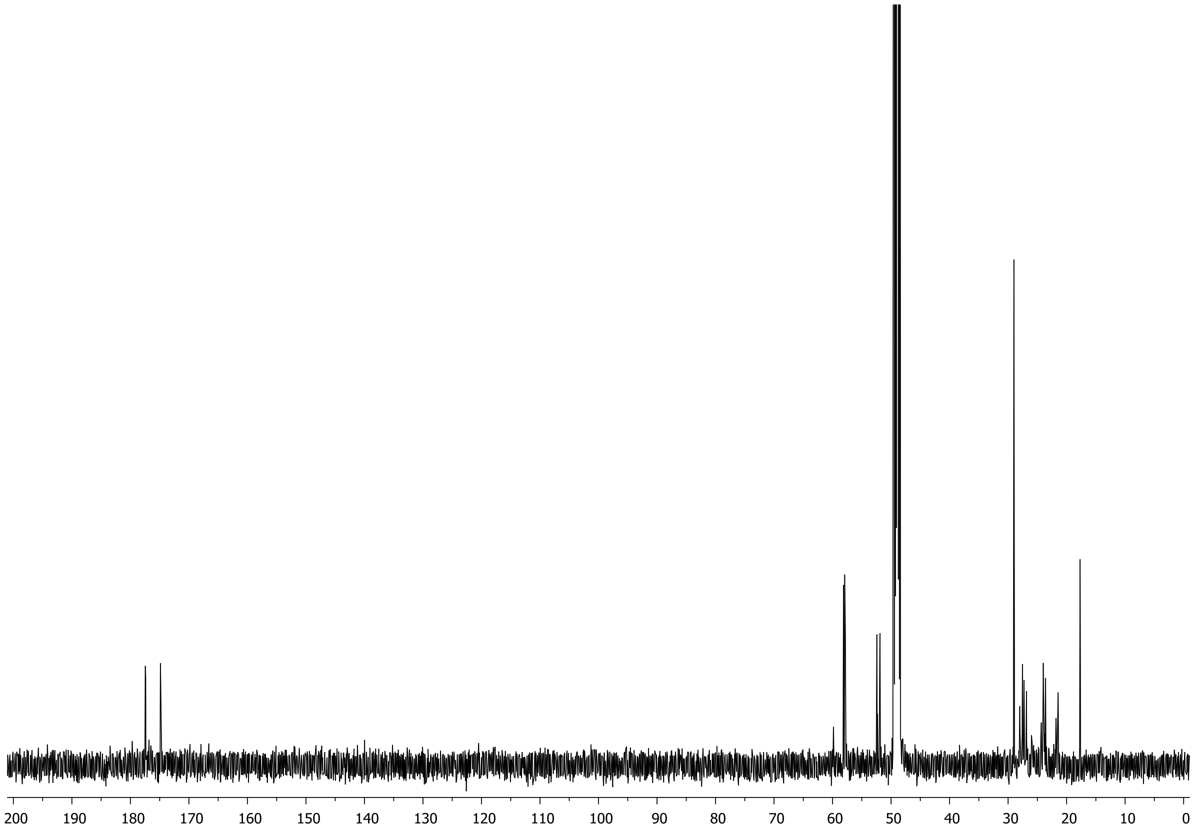

^{1}H-NMR of 1-(2-methyl-2-(piperidin-1-yl)propyl)thiourea-GlyAib$_4$-L-AlaNH^{t}Bu 9d

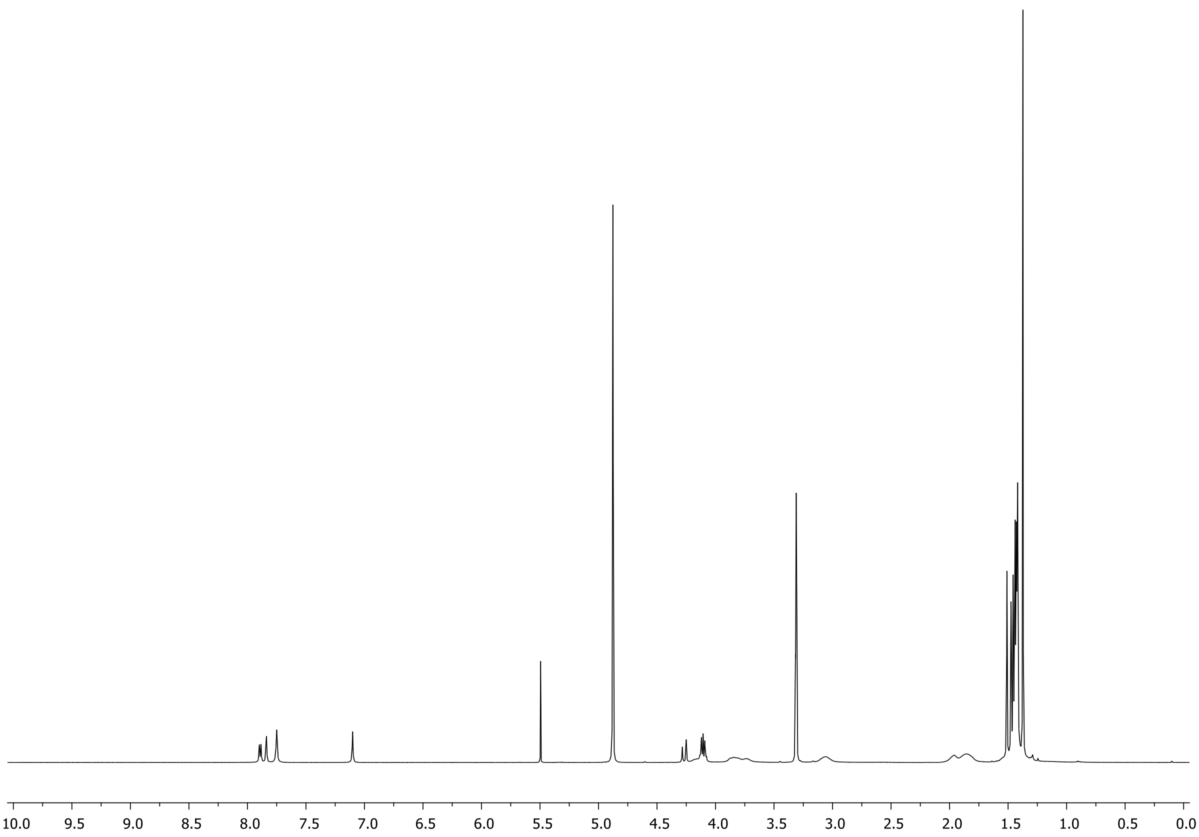

^{13}C-NMR of 1-(2-methyl-2-(piperidin-1-yl)propyl)thiourea-GlyAib$_4$-L-AlaNH^{t}Bu 9d

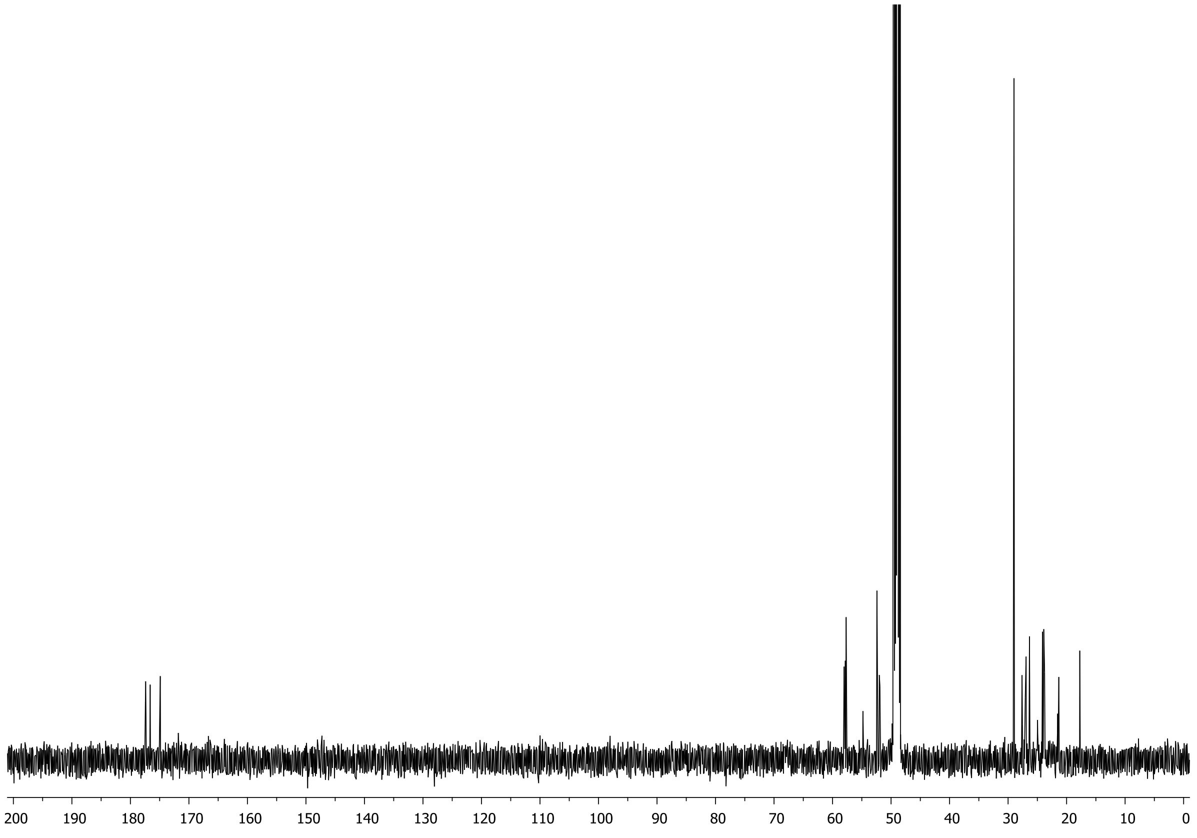

^{1}H-NMR of 1-(2-(piperidin-1-yl)ethyl)thiourea-Aib$_4$-L-AlaNH^{t}Bu 9e

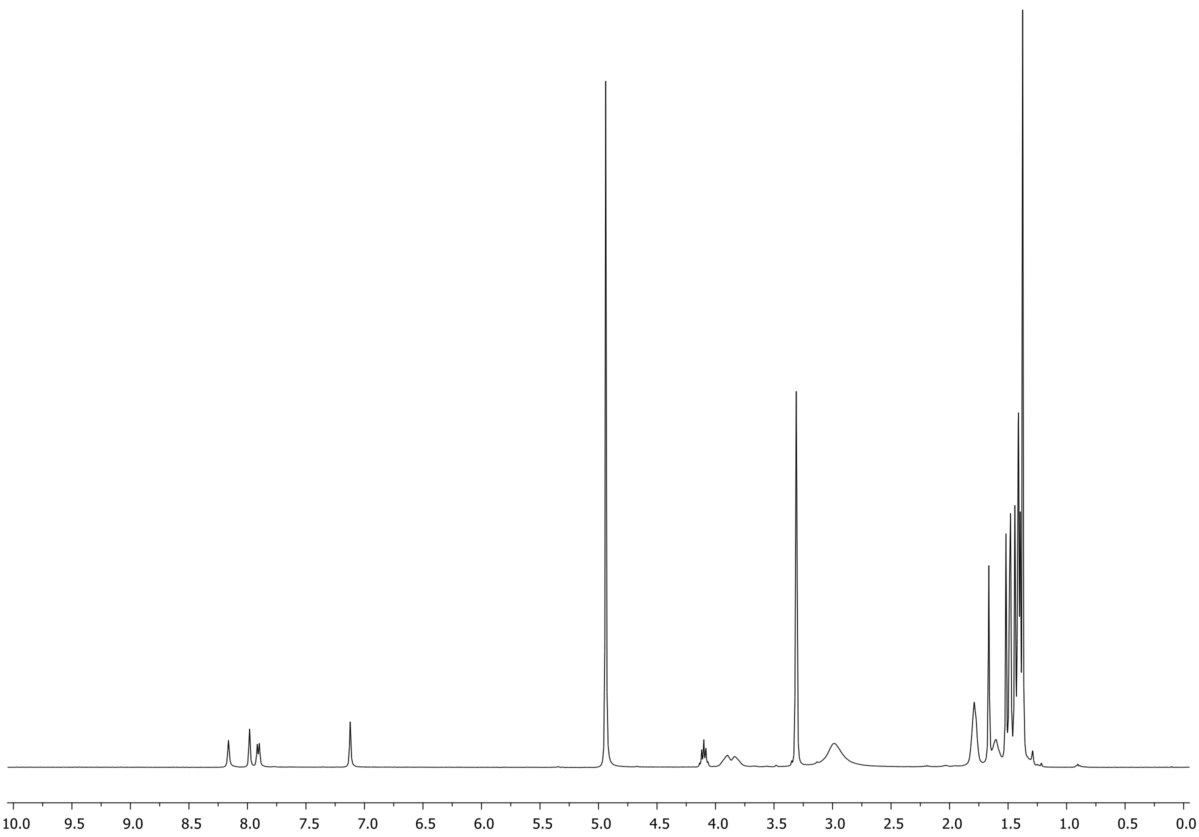

^{13}C-NMR of 1-(2-(piperidin-1-yl)ethyl)thiourea-Aib$_4$-L-AlaNH^{t}Bu 9e

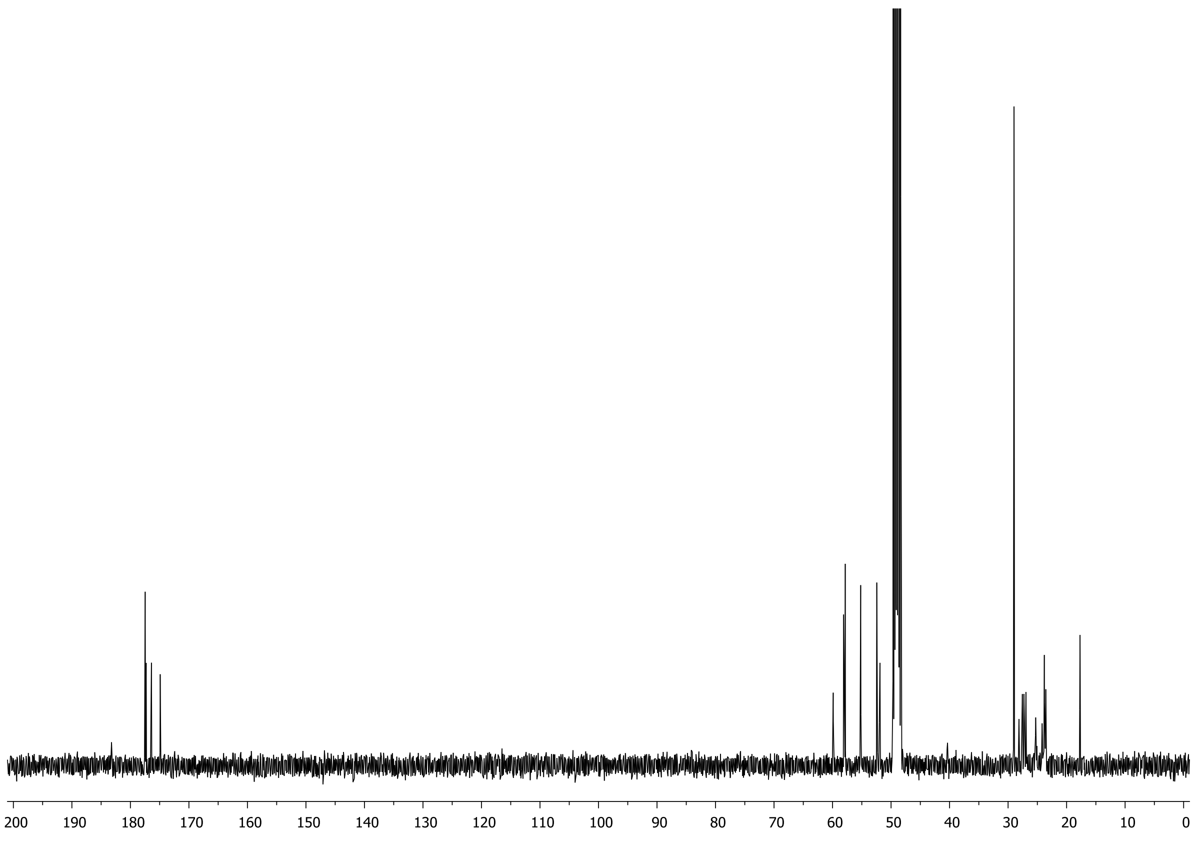

^{1}H-NMR of 1-(2-(piperidin-1-yl)ethyl)thiourea-GlyAib$_4$-L-AlaNH^{t}Bu 9f

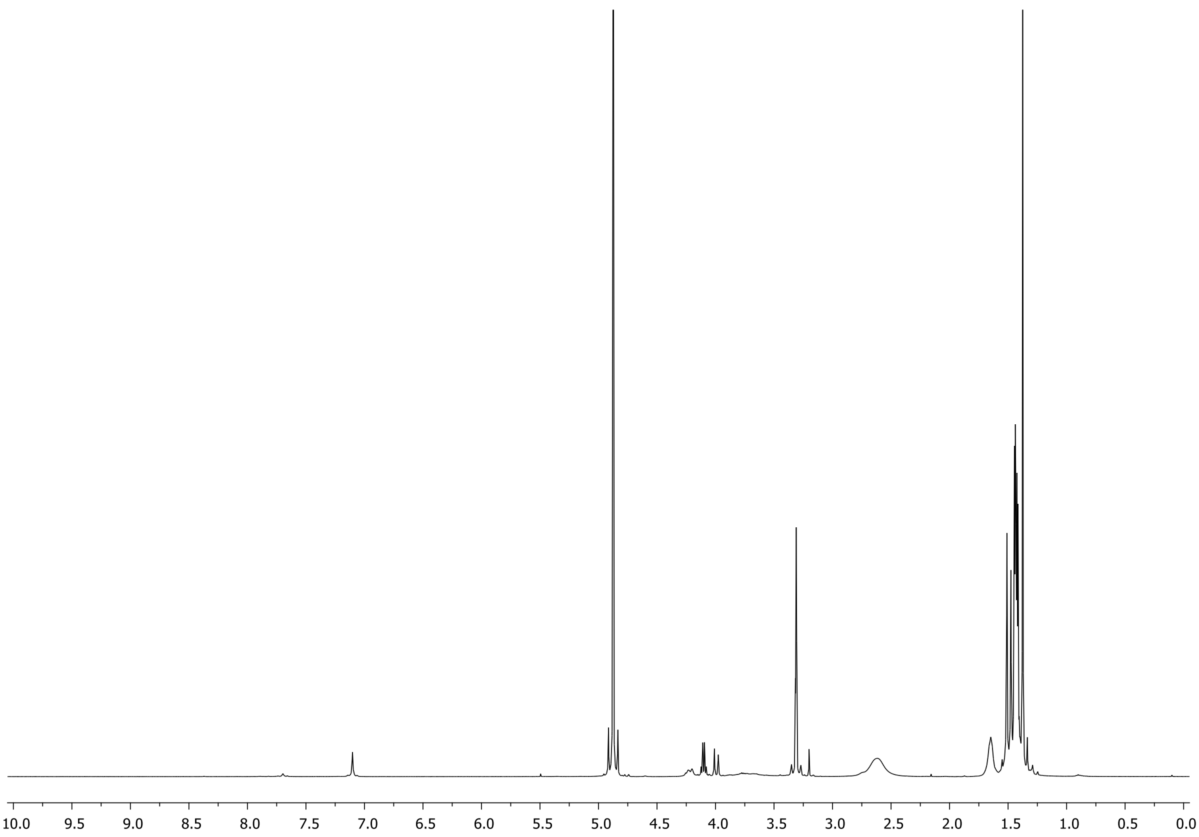

^{13}C-NMR of 1-(2-(piperidin-1-yl)ethyl)thiourea-GlyAib$_4$-L-AlaNH^{t}Bu 9f

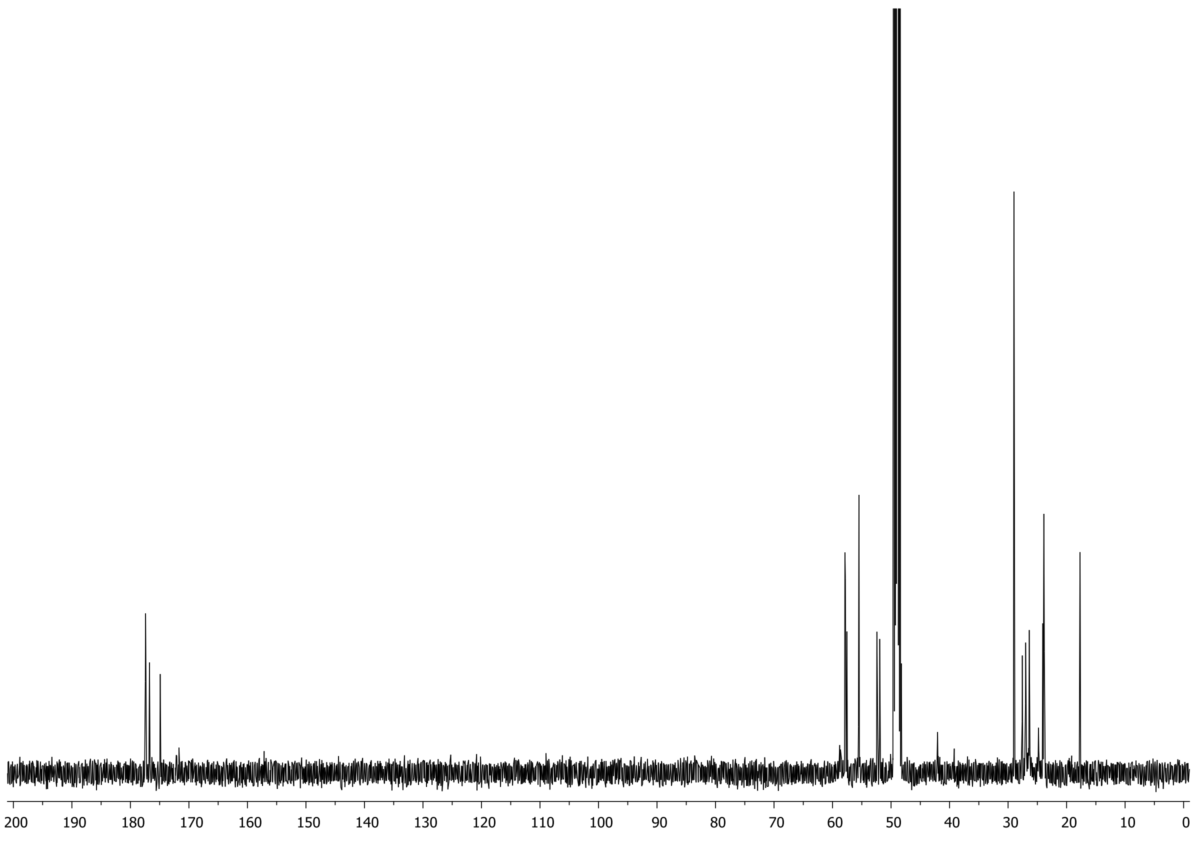

^{1}H-NMR of 1-(2-(piperidin-1-yl)ethyl)thiourea-Aib$_4$O^{t}Bu 9g

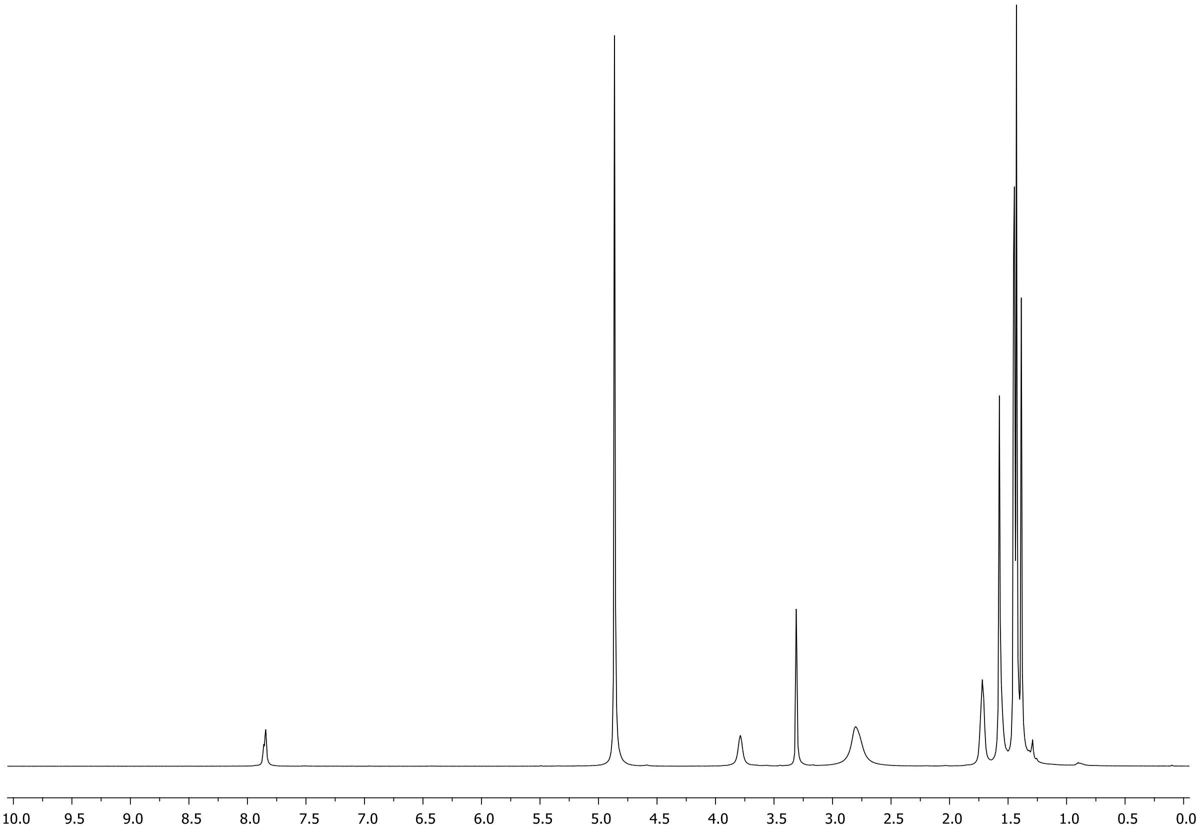

^{13}C-NMR of 1-(2-(piperidin-1-yl)ethyl)thiourea-Aib$_4$O^{t}Bu 9g

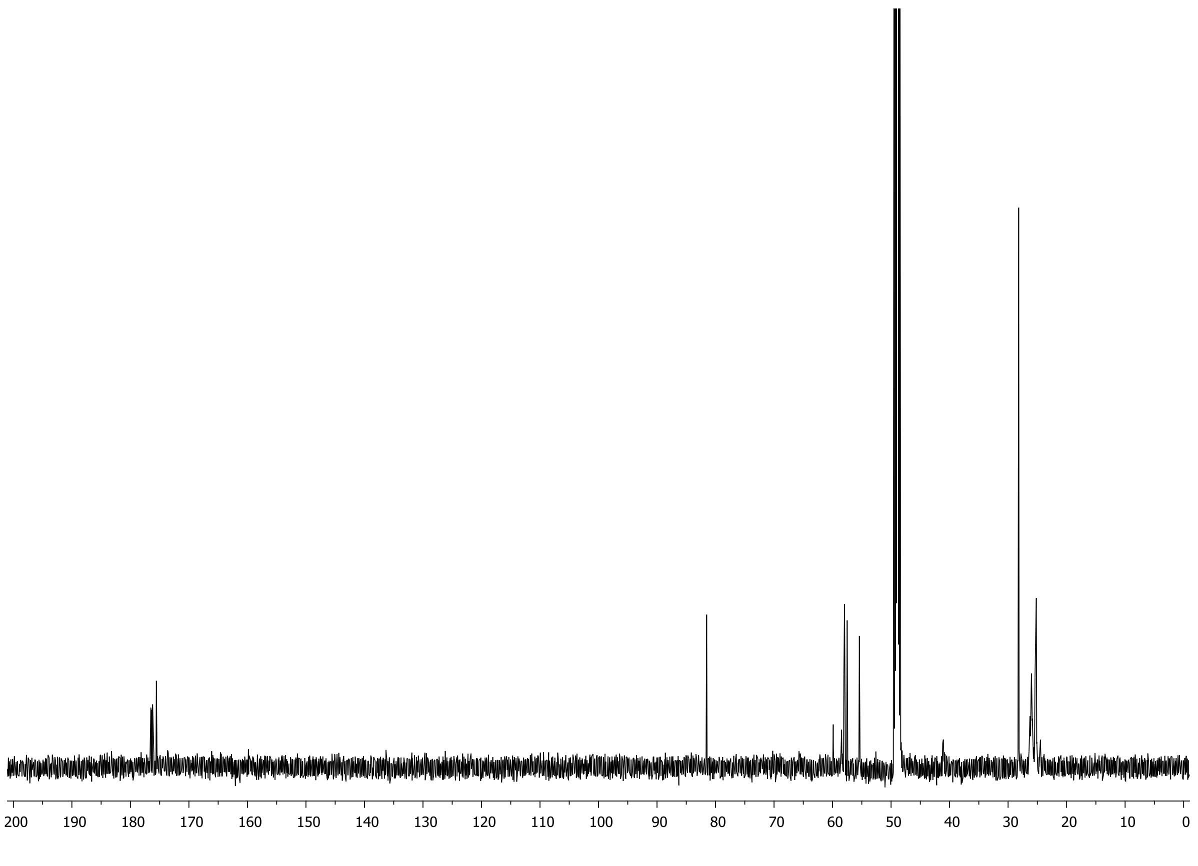

^{1}H-NMR of 1-(2-(piperidin-1-yl)ethyl)thiourea-GlyAib$_4$-L-AlaBni 11

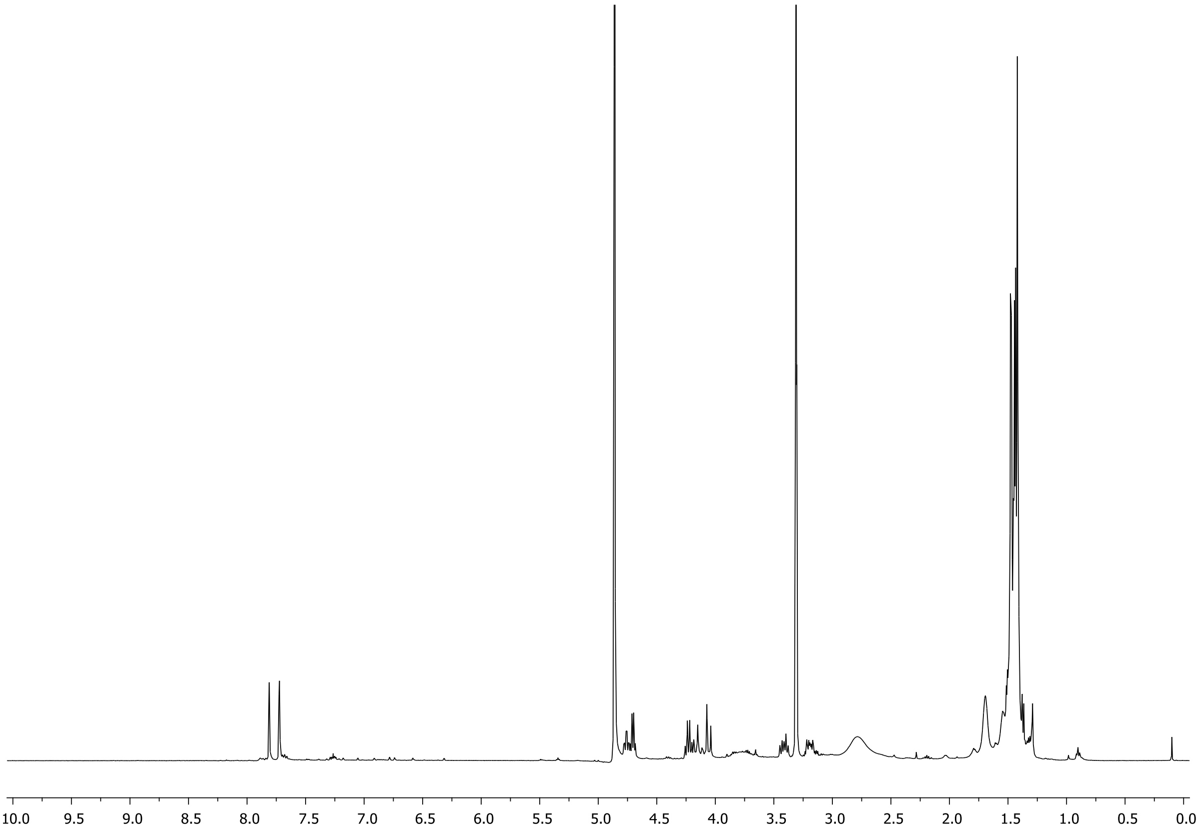

^{13}C-NMR of 1-(2-(piperidin-1-yl)ethyl)thiourea-GlyAib$_4$-L-AlaBni 11

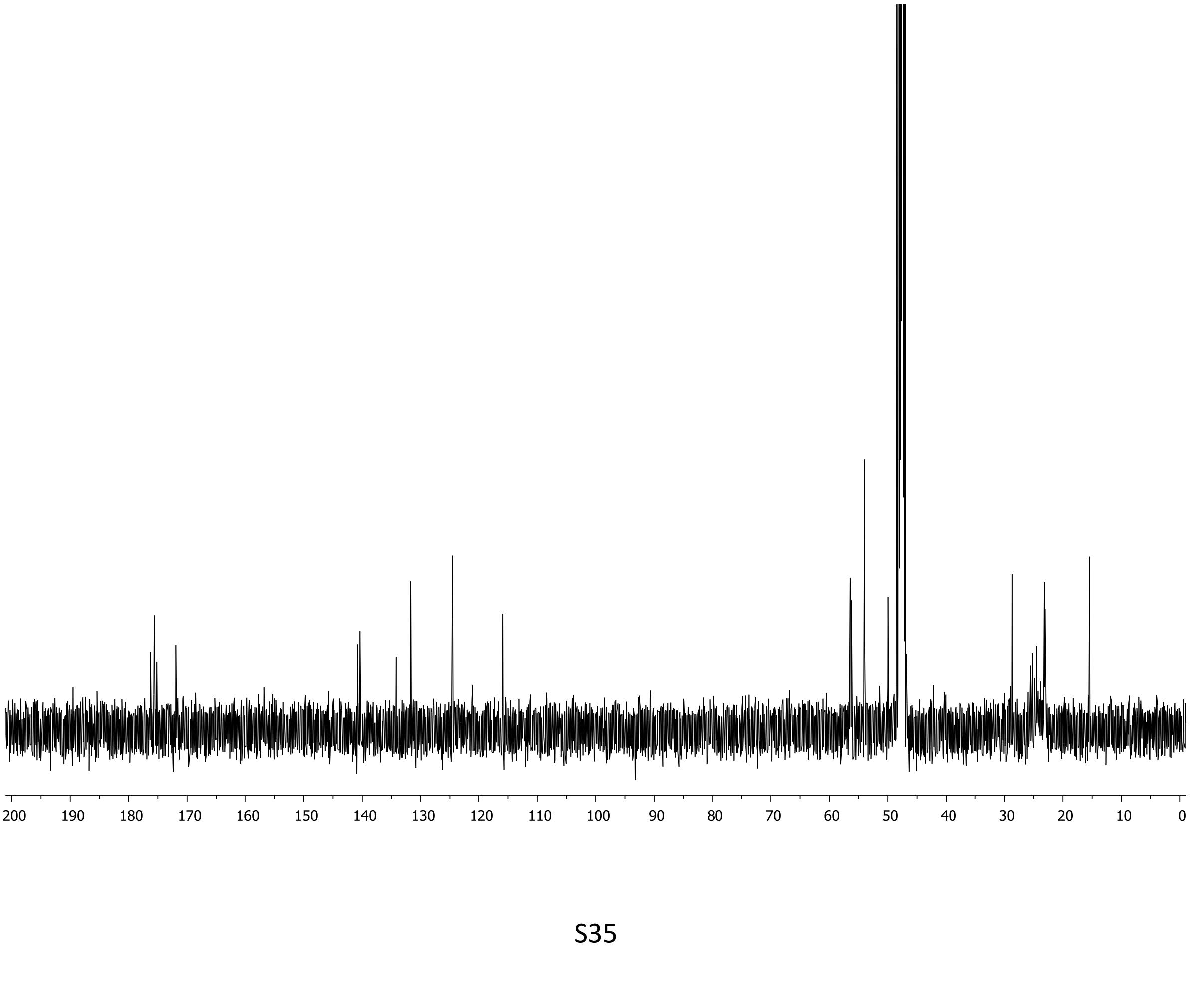

^{1}H-NMR of diethyl 2-(2-nitro-1-phenylethyl)malonate 10 (R = Et)

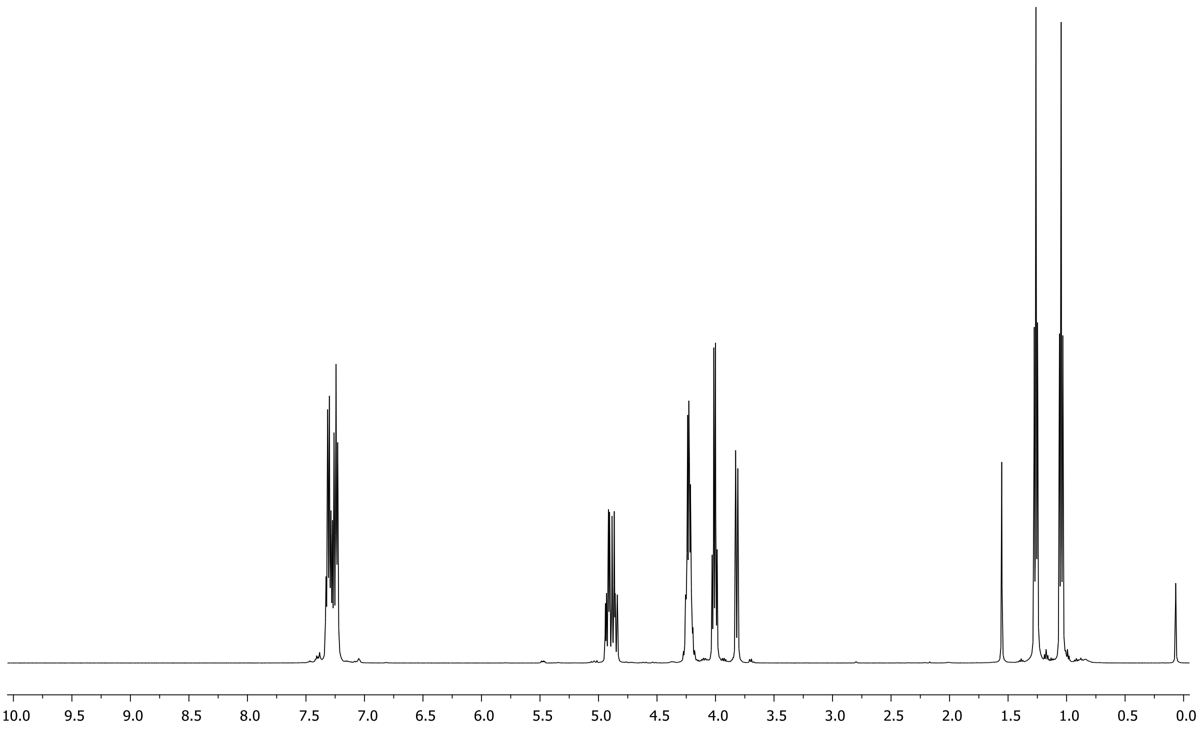

^{13}C-NMR of diethyl 2-(2-nitro-1-phenylethyl)malonate (R = Et)

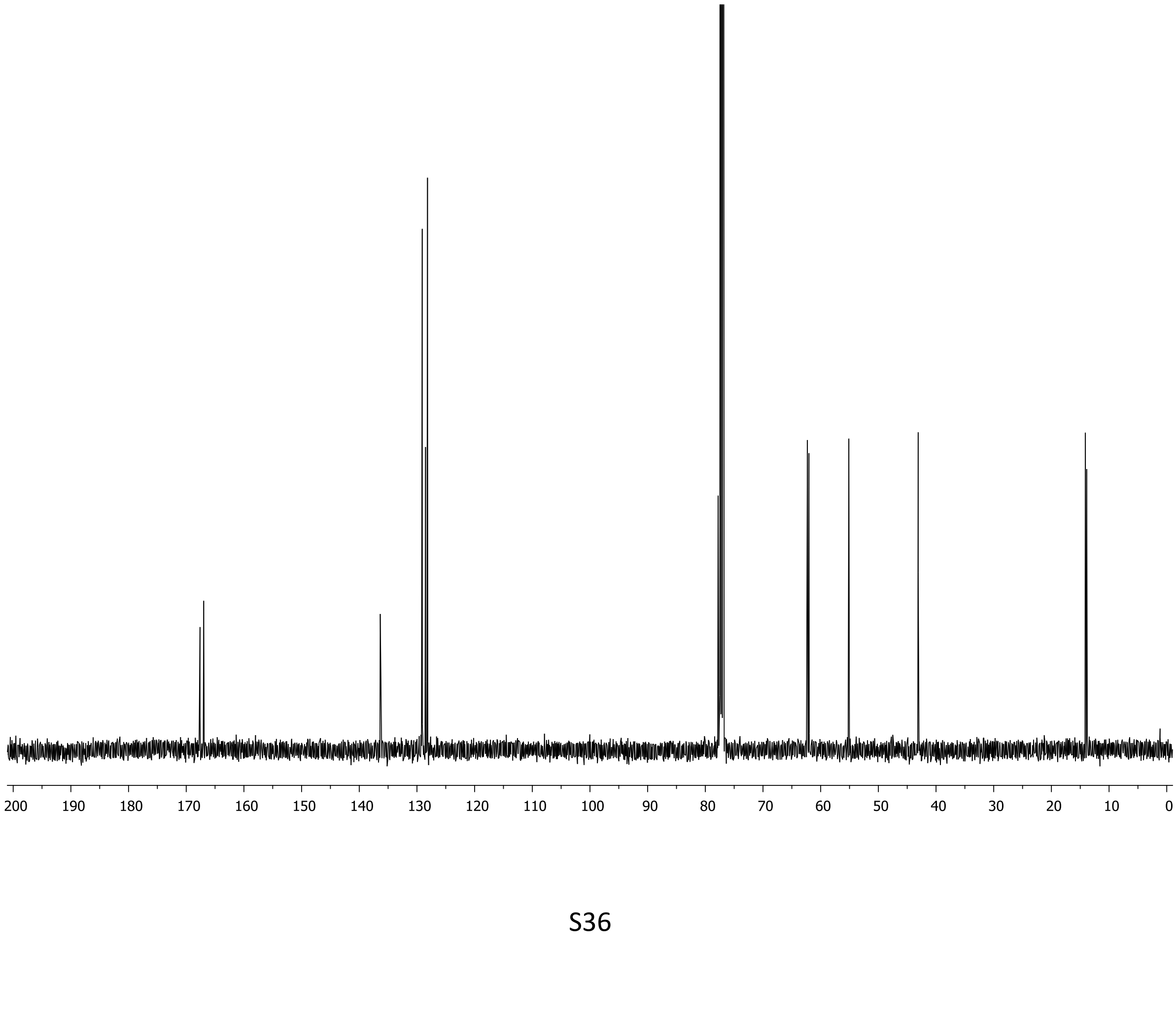

^{1}H-NMR of dimethyl 2-(2-nitro-1-phenylethyl)malonate (R = Me)

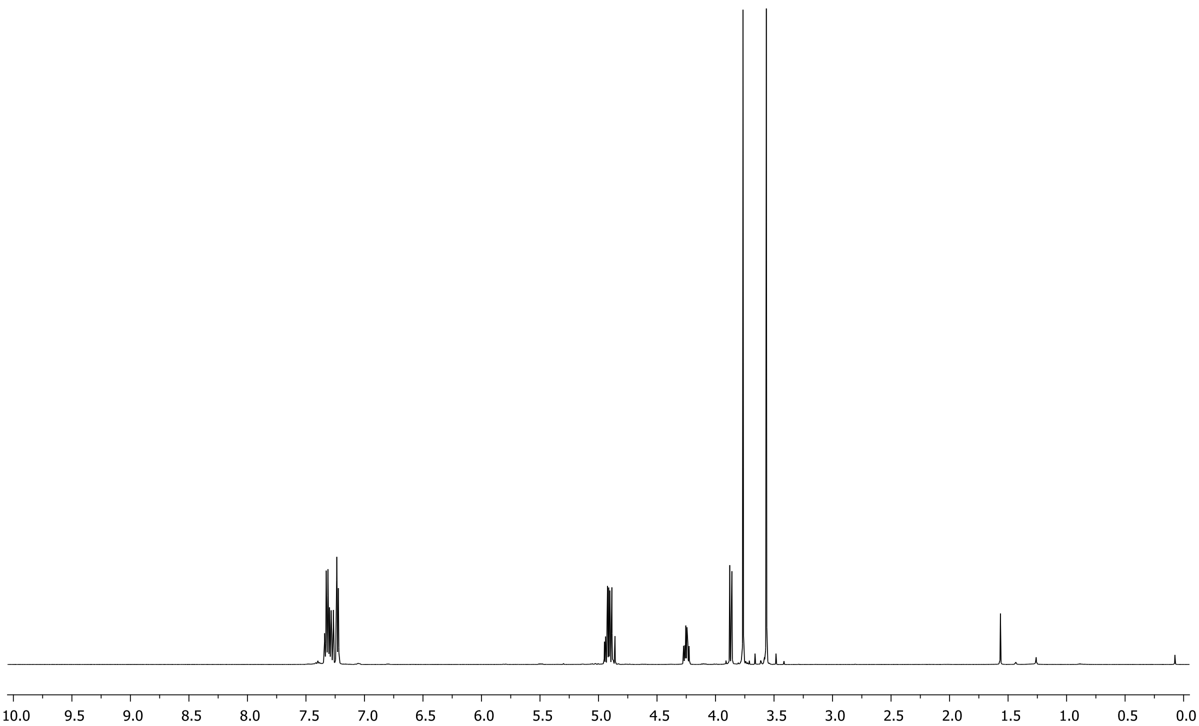

^{13}C-NMR of dimethyl 2-(2-nitro-1-phenylethyl)malonate (R = Me)

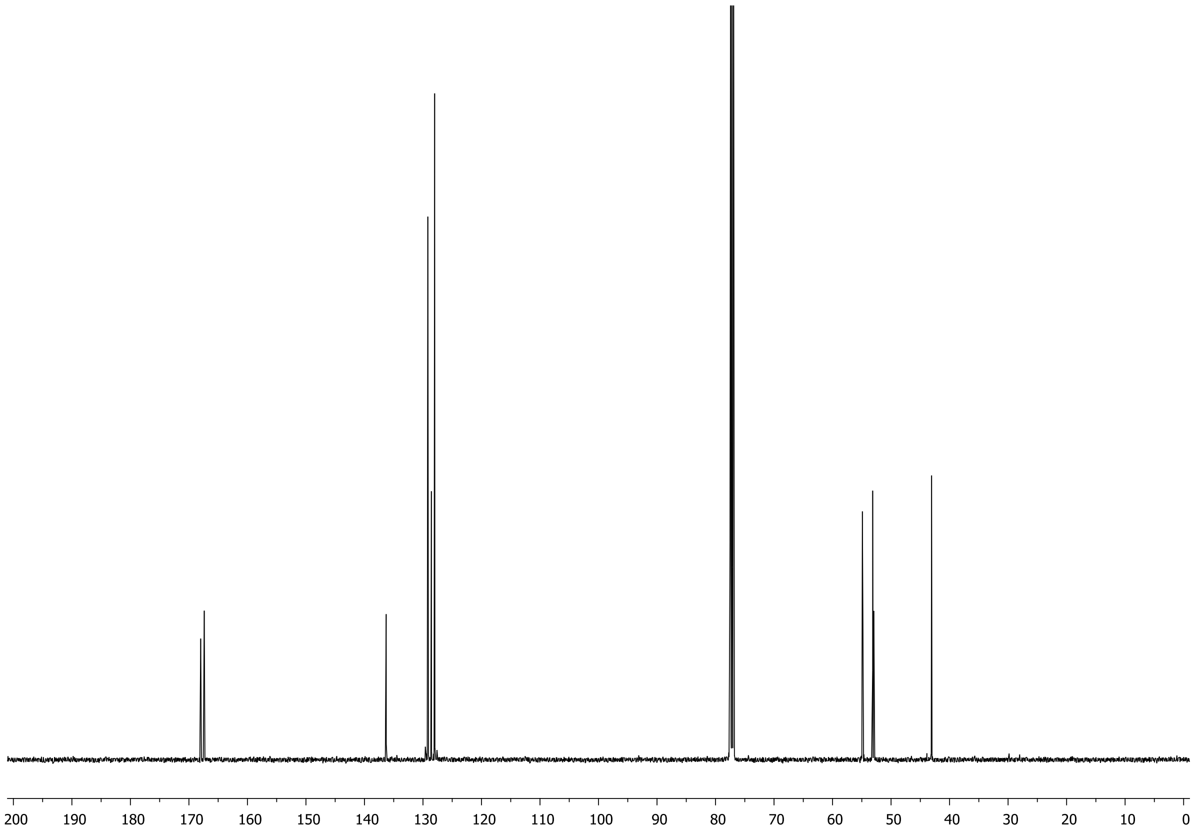

Conversion of 11 to 12 by irradiation at 365 nm with $^{i}PrNH_2$ in CD_2Cl_2 (monitored by ^1H-NMR)

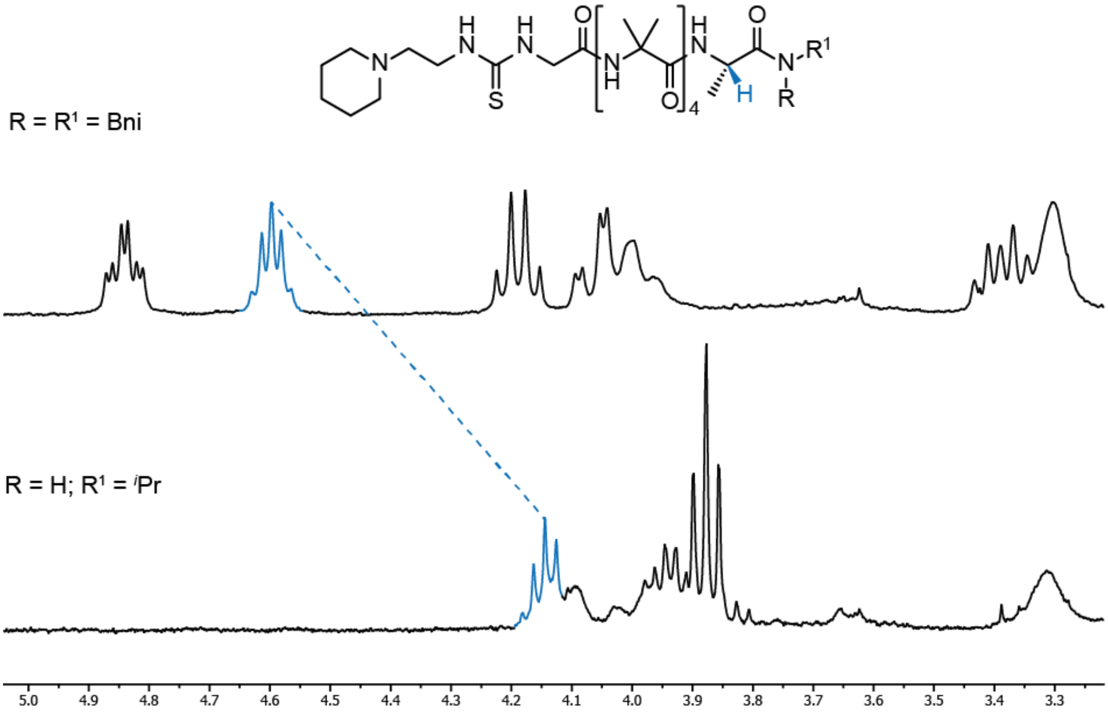

HPLC traces

Diethyl 2-(2-nitro-1-phenylethyl)malonate 10 (R = Et)

Racemate:

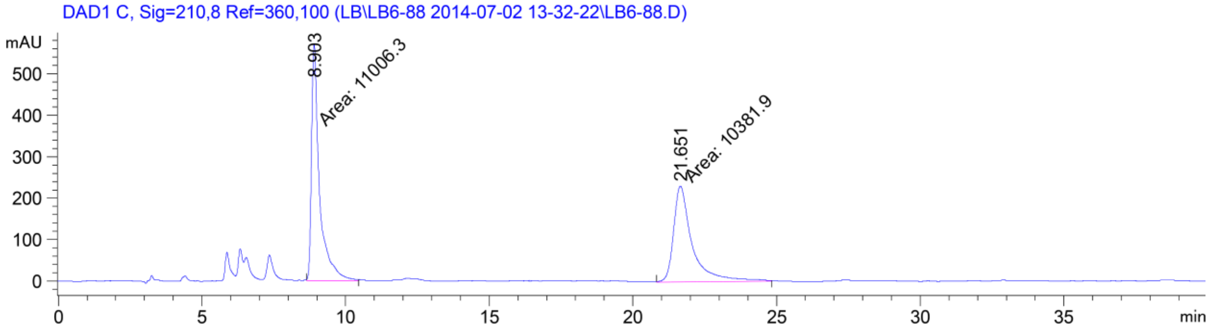

Signal 2: DAD1 C, Sig=210,8 Ref=360,100

Peak #	RetTime [min]	Type	Width [min]	Area [mAU*s]	Height [mAU]	Area %
1	8.903	MM	0.3221	1.10063e4	569.51636	51.4596
2	21.651	MM	0.7496	1.03819e4	230.83968	48.5404

Michael addition with catalyst 9f:

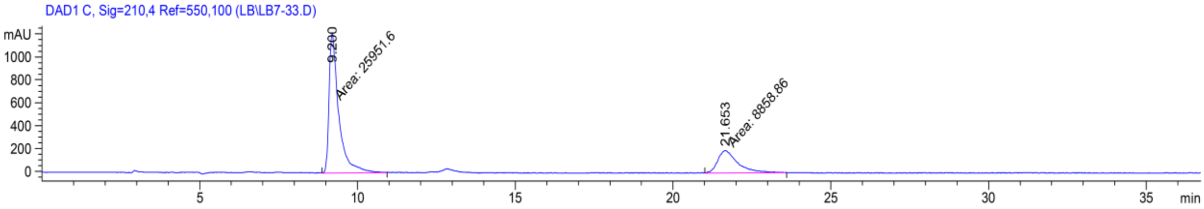

Signal 3: DAD1 C, Sig=210,4 Ref=550,100

Peak #	RetTime [min]	Type	Width [min]	Area [mAU*s]	Height [mAU]	Area %
1	9.200	MM	0.3543	2.59516e4	1220.71790	74.5511
2	21.653	MM	0.7467	8858.85742	197.73703	25.4489

Control Experiment:

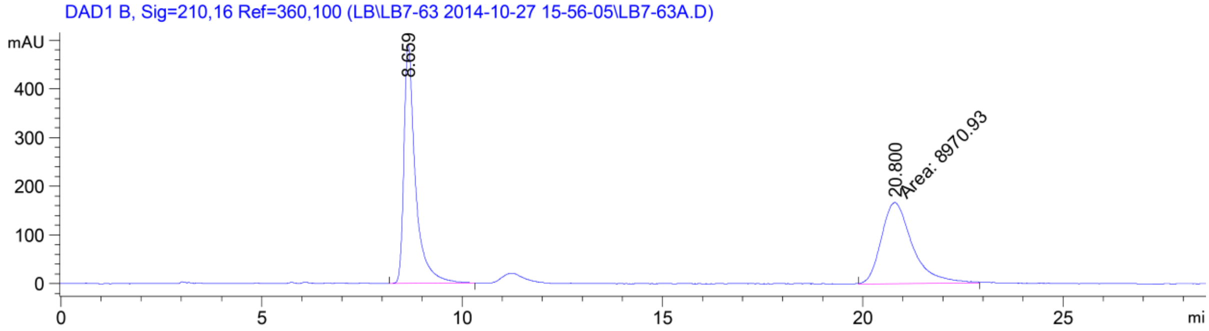

```
Signal 2: DAD1 B, Sig=210,16 Ref=360,100

Peak RetTime Type  Width     Area      Height     Area
  #   [min]        [min]   [mAU*s]     [mAU]       %
----|-------|----|-------|----------|----------|--------|
   1   8.659 VB    0.2910 9811.97266  491.25864  52.2389
   2  20.800 MM    0.8981 8970.92773  166.48714  47.7611
```

Dimethyl 2-(2-nitro-1-phenylethyl)malonate 10 (R = Me)

Michael addition with catalyst 9f:

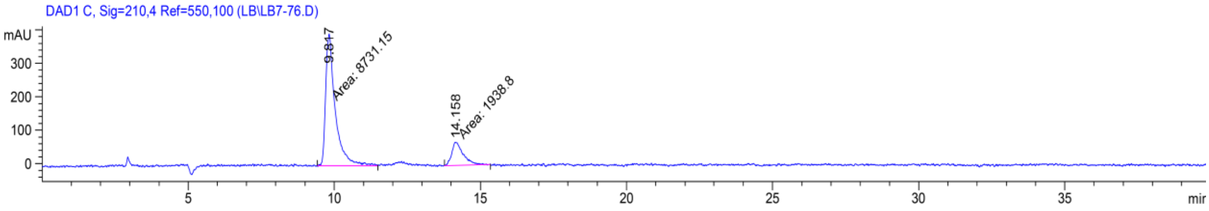


```
Signal 3: DAD1 C, Sig=210,4 Ref=550,100

Peak RetTime Type  Width     Area      Height     Area
  #   [min]        [min]   [mAU*s]     [mAU]       %
----|-------|----|-------|----------|----------|--------|
   1   9.817 MM    0.3687 8731.15039  394.64746  81.8294
   2  14.158 MM    0.4709 1938.79761   68.62613  18.1706
```

Photoswitch experiment (Michael addition with catalyst 11 – before irradiation):

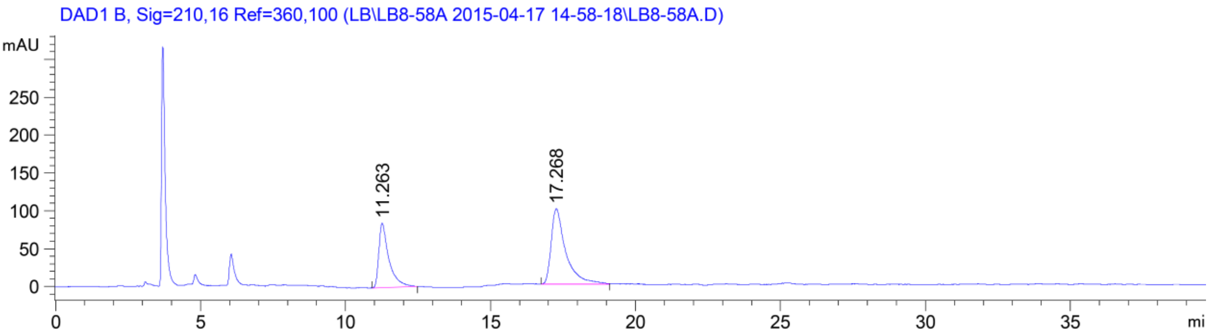


```
Signal 2: DAD1 B, Sig=210,16 Ref=360,100

Peak RetTime Type  Width     Area      Height     Area
  #   [min]        [min]   [mAU*s]     [mAU]       %
----|-------|----|-------|----------|----------|--------|
   1  11.263 BV    0.3440 2062.02710   85.29383  37.2815
   2  17.268 VV    0.4936 3468.93970   99.78661  62.7185
```

Photoswitch experiment (Michael addition with catalyst 12 – after irradiation):

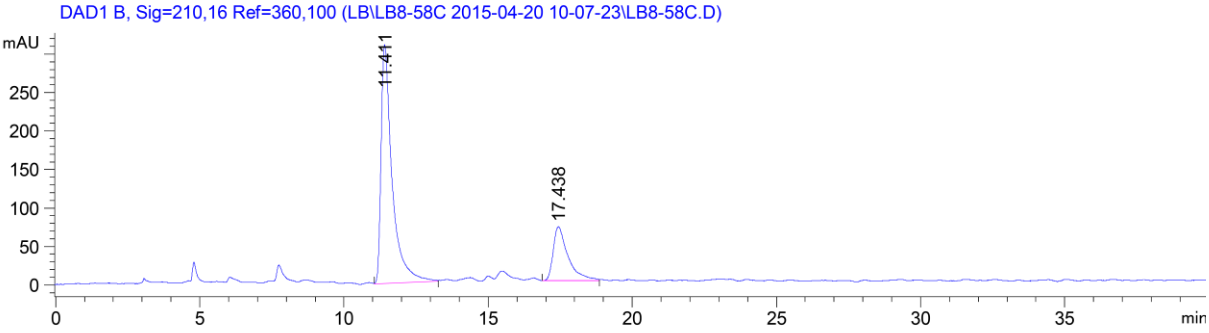

Signal 2: DAD1 B, Sig=210,16 Ref=360,100

Peak #	RetTime [min]	Type	Width [min]	Area [mAU*s]	Height [mAU]	Area %
1	11.411	VV	0.3910	8276.01660	310.46045	76.5418
2	17.438	BV	0.5080	2536.40479	70.46012	23.4582

References

1. J. Clayden, A. Castellanos, J. Solà and G. A. Morris, *Angew. Chem. Int. Ed.*, **2009**, *48*, 5962-5965
2. B. A. F. Le Bailly, J. Clayden, *Chem. Commun.*, **2014**, *50*, 7949-7952
3. Y. Hiroshi, O. Takashi, O. Tatsuo, Y. Kentaro, S. Naoko, N. Takeshi, O. Yoichi, K. Nozomu and K. Kyosuke, US Patent US5721246 (A), 1998
4. T. Ramdahl, US Patent US20130183235 A1, 2013
5. M. Lee, L. Zhang, Y. Park and H.-G. Park, *Tetrahedron* **2012**, *68*, 1452-1459
6. K. Wilckens, M.-A. Duhs, D. Lentz and C. Czekelius, *Eur. J. Org. Chem.* **2011**, 5441-5446
